# Dual Target Ligands with 4-*tert*-Butylphenoxy Scaffold as Histamine H_3_ Receptor Antagonists and Monoamine Oxidase B Inhibitors

**DOI:** 10.3390/ijms21103411

**Published:** 2020-05-12

**Authors:** Dorota Łażewska, Agnieszka Olejarz-Maciej, David Reiner, Maria Kaleta, Gniewomir Latacz, Małgorzata Zygmunt, Agata Doroz-Płonka, Tadeusz Karcz, Annika Frank, Holger Stark, Katarzyna Kieć-Kononowicz

**Affiliations:** 1 Department of Technology and Biotechnology of Drugs, Jagiellonian University Medical College, 9 Medyczna Str, 30-688 Kraków, Poland; agnieszka.olejarz@uj.edu.pl (A.O.-M.); maria.kaleta@uj.edu.pl (M.K.); glatacz@cm-uj.krakow.pl (G.L.); a.doroz-plonka@uj.edu.pl (A.D.-P.); t.karcz@uj.edu.pl (T.K.); 2 Institute of Pharmaceutical and Medicinal Chemistry, Heinrich Heine University Düsseldorf, Universitaetsstr. 1, 40225 Duesseldorf, Germany; david.reiner@hhu.de (D.R.); a.frank@hhu.de (A.F.); stark@hhu.de (H.S.); 3 Department of Pharmacodynamics, Jagiellonian University Medical College, 9 MedycznaStr, 30-688 Kraków, Poland; malgorzata.zygmunt@uj.edu.pl

**Keywords:** dual-target ligands, histamine H_3_ receptor, monoamine oxidase B, 4-*tert*-butylphenyl derivatives, antagonists, inhibitors, neurodegenerative disease, Parkinson’s disease

## Abstract

Dual target ligands are a promising concept for the treatment of Parkinson’s disease (PD). A combination of monoamine oxidase B (MAO B) inhibition with histamine H_3_ receptor (H_3_R) antagonism could have positive effects on dopamine regulation. Thus, a series of twenty-seven 4-*tert*-butylphenoxyalkoxyamines were designed as potential dual-target ligands for PD based on the structure of 1-(3-(4-*tert*-butylphenoxy)propyl)piperidine (**DL76**). Probed modifications included the introduction of different cyclic amines and elongation of the alkyl chain. Synthesized compounds were investigated for human H_3_R (hH_3_R) affinity and human MAO B (hMAO B) inhibitory activity. Most compounds showed good hH_3_R affinities with K_i_ values below 400 nM, and some of them showed potent inhibitory activity for hMAO B with IC_50_ values below 50 nM. However, the most balanced activity against both biological targets showed **DL76** (hH_3_R: K_i_ = 38 nM and hMAO B: IC_50_ = 48 nM). Thus, **DL76** was chosen for further studies, revealing the nontoxic nature of **DL76** in HEK293 and neuroblastoma SH-SY5Ycells. However, no neuroprotective effect was observed for **DL76** in hydrogen peroxide-treated neuroblastoma SH-SY5Y cells. Furthermore, in vivo studies showed antiparkinsonian activity of **DL76** in haloperidol-induced catalepsy (Cross Leg Position Test) at a dose of 50 mg/kg body weight.

## 1. Introduction

**Figure 1:**
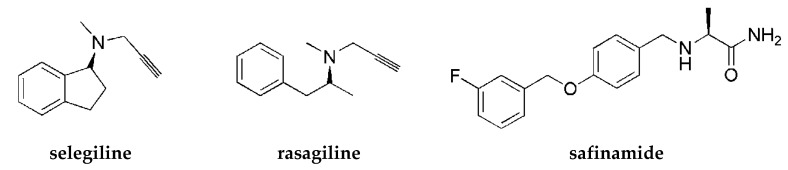
Structures of antiparkinsonian drugs—monoamine oxidase (MAO) B inhibitors.

Parkinson’s disease (PD) is a progressive neurodegenerative disorder characterized by motor problems. Although the entire pathology of PD is still unknown, several factors have been proposed to contribute to PD development, such as environmental toxins, neuroinflammation, genetic mutations, oxidative stress, or mitochondrial dysfunction [1]. Generally, PD is characterized by a severe lack of dopamine (DA) (80–90%) in *striatum* due to a progressive loss of dopaminergic neurons in the *substantia nigra* [1]. Current therapy for PD can only mitigate symptoms and slow the progress. However, there is no cure for the disease to date. Commonly administered drugs include levodopa (as a DA precursor), DA agonists (e.g., pramipexole and rotigotine), and monoamine oxidase (MAO) B inhibitors (e.g., selegiline, rasagiline, or safinamide; Figure 1).

MAO B plays a crucial role in the pathogenesis of PD. This enzyme belongs to the family of MAOs that catalyze the deamination of neurotransmitters (e.g., DA) and release reactive oxygen species as by-products. MAO B dominates in the human brain and deaminates β-phenylethylamine (PEA). PEA increases the synaptic levels of DA and blocks its reuptake into neurons. An increase in the activity of MAO B with age and some diseases as PD was observed in humans. Inhibitors of MAO B stop the activity of this enzyme and block the breakdown of DA. Moreover, MAO B inhibitors show neuroprotection and reduce oxidative stress [2]. Thus, MAO B inhibition is an important factor in the search for effective drugs in the treatment of PD. However, due to the multifactorial etiology of PD, it is thought that ligands acting on several targets at the same time (so-called Multi-Target-Directed Ligands (MTDL)) will be more effective in treatment than a one-target compound [3]. Thus, for improving the pharmacotherapy of PD, it is important to find MAO B inhibitors with combined activity at other targets.

**Figure 2:**
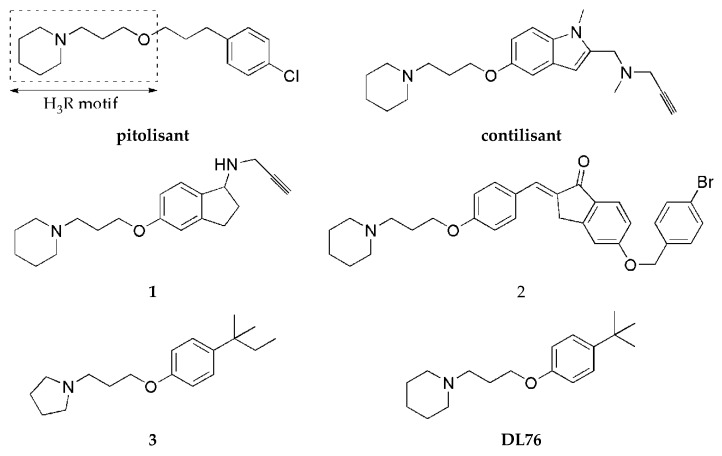
Structures of pitolisant and histamine H_3_ receptor ligands with MAO B inhibitory activity.

Histamine H_3_ receptors (H_3_Rs) are widely distributed in the human brain and dominantly in areas connected with cognition (such as the striatum, cortex, or hippocampus). H_3_Rs influence the release not only of histamine itself but also of other neurotransmitters, such as DA or acetylcholine [4], and increase the level of mentioned neurotransmitters in the synaptic cleft. Numerous pharmacological studies show that blocking H_3_Rs provides beneficial effects in the treatment of various neurological diseases such as narcolepsy, neurodegenerative diseases (e.g., Alzheimer’s disease and PD), attention deficit hyperactivity disorder, epilepsy, obesity, or neuropathic pain [5]. For years, many scientific centers and pharmaceutical companies have been involved in the search for active ligands of these receptors. Intensive synthetic work has led to a large number of structurally diverse compounds. Some of them have reached clinical trials, but so far, only one (pitolisant (Wakix^®^); Figure 2) has entered into the market as an orphan drug for narcolepsy [6].

One strategy to obtain MTDL is to combine two or more pharmacophores into a single molecule. Pharmacophores can be connected by linkers, attached directly (fused), or merged [7]. A propargylamine moiety is known to be important for MAO B inhibition [8], and it is present in the marketed drugs selegiline and rasagiline. The piperidinepropoxy motif is a part of many potent H_3_R ligands, e.g., pitolisant (Figure 2). The idea to combine MAO B inhibition with H_3_R antagonism is quite new. In 2017, the first of such compounds, contilisant (Figure 2), was described by Bautista-Aguilera et al. [9]. Contilisant not only proved to be a H_3_R antagonist (K_i_ = 11 nM) and human MAO B (hMAO B) inhibitor (IC_50_ = 78 nM) but also showed moderate inhibition of cholinesterases (AChE IC_50_ = 530 nM; BuChE IC_50_ = 1690 nM). Further, this idea was continued by Lutsenko et al. [10] with the fused rasagiline derivative **1** as a dual-target ligand (DTL) with high hH_3_R affinity (K_i_ = 6.7 nM) and good hMAO B inhibitory activity (IC_50_ = 256 nM) (Figure 2). Moreover, Affini et al. [11] described indanone derivatives as DTL for PD (compound **2**; hH_3_R K_i_ = 6.5 nM; hMAO B IC_50_ = 276 nM; Figure 2). Recently, we have described a new group of DTL hMAO B inhibitors, *tert*-amylphenoxy derivatives [12]. These compounds showed also affinity for hH_3_R (e.g., compound **3**; Figure 2). In contrast to the previously described DTL (contilisant, **1** and **2**), some of them showed an inhibitory activity for hMAO B that was higher than their affinity for hH_3_R (**3**: hH_3_R K_i_ = 63 nM; hMAO B IC_50_ = 4.5 nM).

To continue this work, we synthesized a series of 4-*tert*-butylphenoxy derivatives as analogues of histamine H_3_R ligand **DL76** (hH_3_R K_i_ = 22 nM in CHO K1 cells [13]; Figure 2) which showed also good inhibitory activity for hMAO B with an IC_50_ of 48 nM. Encouraged by these results, we designed a series of novel 4-*tert*-butylphenoxy derivatives. Designed structural modifications included the following:

- exchange of piperidine moiety for other cyclic amines (pyrrolidine, substituted piperidine, or azepane)
- elongation of alkyl chain from three up to six atoms.

All compounds were evaluated for their affinity towards hH_3_R and inhibition of hMAO B. Furthermore, we selected one of compounds for antiparkinsonian activity tests in vivo (in haloperidol-induced catalepsy) and neuroprotection studies in vitro (in neuroblastoma SH-SY5Y cells). Moreover, the toxicity of this compound in HEK293 cells and neuroblastoma SH-SY5Y cells was evaluated.

## 2. Results and Discussion

### 2.1. Synthesis of Compounds

**Scheme 1:**
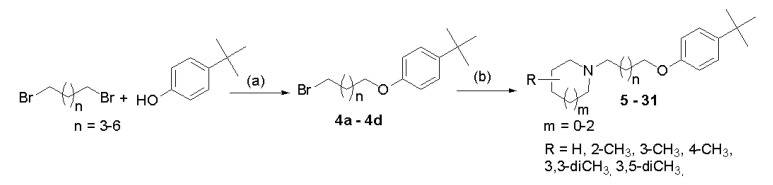
General synthetic pathway of synthesized compounds **5**–**31**. Reagents and conditions: (**a**) sodium propanolate (0.05 mol Na in 50 mL), room temperature, 15′; 60 °C 3 h; reflux 3 h; 69–78%; (**b**) (i) amine, K_2_CO_3_, KI, EtOH:H_2_O (21:4), and reflux 20 h and (ii) oxalic acid, EtOH, room temperature, 1 h; (Et)_2_O; crystallization from EtOH, 5–53%.

Compounds were synthesized as shown in Scheme 1. Briefly, proper 4-*tert*-butylphenoxyalkyl bromides (**4a**–**4d**) were obtained by nucleophilic substitution of 4-*tert*-butylphenol with α,ω-dibromoalkanes in freshly prepared sodium propanolate as described previously [14]. Then, the bromides **4a**–**4d** were refluxed with corresponding amines in the mixture of ethanol–water (21:4) with powdered potassium carbonate and a catalytic amount of potassium iodide. The purified free bases were converted into hydrogen oxalates. Structures and purity of novel compounds **5–31** were confirmed by spectral analyses (^1^H and ^13^C NMR spectra; see Supplementary Materials S1), mass spectrometry (MS), and elemental analysis.

### 2.2. Human Histamine H_3_ Receptor Affinity

The affinity of compounds (**5**–**31**) for H_3_R was evaluated in a radioligand-binding assay in HEK293 cells stably expressing hH_3_R [14]. [^3^H]*N*^α^-methylhistamine was used as a radioligand. Results are presented as K_i_ values in Table 1. The compounds showed variable affinities for hH_3_R ranging from good (K_i_ < 100 nM) to weak (K_i_ > 1500 nM), depending on the kind of the introduced cyclic amine moiety and alkyl chain length. Analyzing the influence of a cyclic amine moiety on the affinity for hH_3_R, it was noticed that derivatives of piperidine (**DL76**, **6**–**8**), 3-methylpiperidine (**12**–**15**), and azepane (**28**–**31**) were the most active. On the other hand, 3,3-dimethylpiperidine derivatives (**16**–**19**) showed weaker affinities (K_i_ > 1600 nM). Unfortunately, none of the synthesized compounds (**5**–**31**) showed higher hH_3_R affinity than the lead structure **DL76** (K_i_ = 38 nM). The most potent was compound **9** with a K_i_ of 69 nM.

### 2.3. Human Monoamine Oxidase B Inhibitory Activity

#### 2.3.1. Screening and Determination of IC_50_

The inhibitory activity of the compounds against hMAO B was evaluated using Amplex Red^®^ Monoamine Oxidase kits [15]. Rasagiline, pargyline, and safinamide were used as reference inhibitors. Following the initial screening of compounds at 1 μM concentration, IC_50_ values were determined for those which exhibited inhibitory activity greater than 50% (Table 1). Sixteen compounds out of the thirty-one showed a percentage of inhibition between 10% and 41%. Results of IC_50_ determinations indicated the influence of both an amine moiety and a length of alkyl chain on hMAO B inhibition. Except the series of 3,5-dimethylpiperidine and 4-methylpiperidine derivatives, the inhibitory activity of the compounds was more pronounced with a shorter (three or four) alkyl linker. Thus, an elongation of alkyl chain caused a drop of activity. The highest activity was observed for the compounds with the propylene linker (**DL76**, **9**, **12, 16**, **20**, **24**, and **28**). This observation is simillar to our previous finding [12] concerning 4-*tert*-amylphenoxy derivatives as DTL ligands. Exchange of a cyclic amine group (piperidine in **DL76**) for other moieties (pyrrolidine, substituted piperidine, or azepane) caused variable influences: increased (pyrrolidine-**5** or 2-methylpiperidine-**9**), maintained (azepane-**28**), or decreased activity (3-methylpiperidine-**12**, 3,3-dimethylpiperidine-**16**, 3,5-dimethylpiepridine-**20**, or 4-methylpiperidine-**24**). The most potent inhibitor **5** (IC_50_ = 2.7 nM) showed higher activity than the reference compounds rasagiline (IC_50_ = 15 nM) and safinamide (IC_50_ = 7.7 nM).

#### 2.3.2. Reversibility Studies

**Figure 3:**
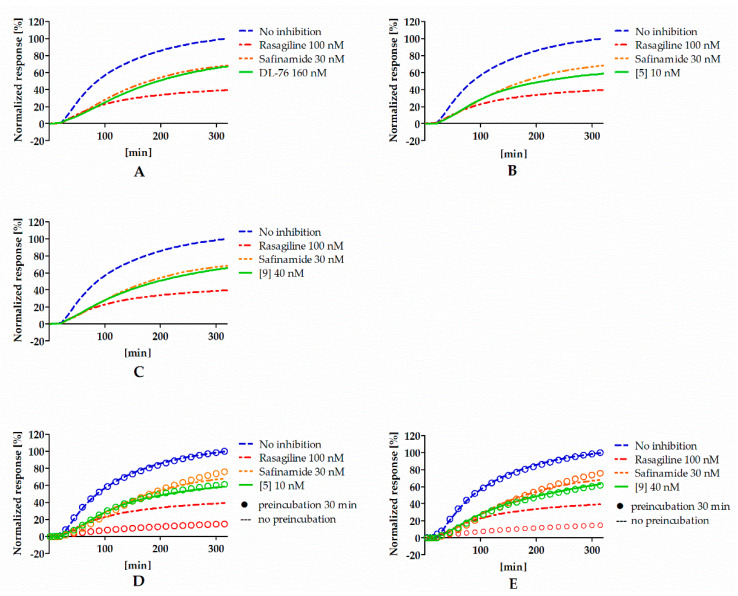
Reversibility of **DL76** (**A**), **5** (**B**), **9** (**C**), and reference inhibitors without the preincubation and comparison of reversibility of **5** (**D**), **9** (**E**), and reference inhibitors that were (rings) or were not (lines) preincubated with hMAOB. Concentrations of compounds correspond to their IC_80_ values.

To investigate the type of inhibition (reversible or irreversible) of hMAO B by 4-*tert*-butylphenoxy derivatives, experiments were performed with selected compounds (**DL76, 5**, and **9**) and conducted as described previously [12]. Rasagiline (irreversible inhibitor) and safinamide (reversible inhibitor) were used as standards. Results from the experiment are shown in Figure 3A–C. All tested compounds presented a signal similar to the reversible reference inhibitor safinamide so they were considered as reversible. However, the signal for rasagiline was slightly higher than expected for the IC_80_ concentration. High signal for rasagiline can be explained by the fact that irreversible inhibitors need more time to create the covalent bond with the enzyme. The used protocol for the reversibility testing did not contain the preincubation of enzyme with inhibitors. Lack of preincubation could also change the observed reversibility curves for reversible inhibitors because they could present different affinity towards the free enzyme and the enzyme that was bound to the substrate [17]. Thus, the experiment was modified and the reversibiblity of investigated inhibitors with and without the preincubation with the enzyme was performed (see Materials and Methods). Results from the experiments after the modification are shown in Figure 3D,E. Safinamide, **5**, and **9** did not show differences between experiments with and without the preincubation, suggesting that the inhibitors did not bind covalently to the enzyme. On the other hand, rasagiline (irreversible inhibitor) showed higher inhibition when preincubated with hMAOB.

#### 2.3.3. Kinetic Studies

**Figure 4:**
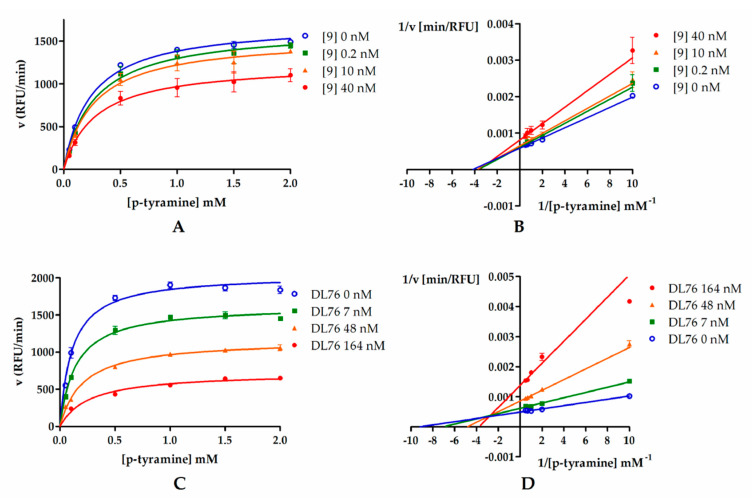
Michaelis–Menten curves for compound **9** (**A**) and **DL76** (**C**): Inhibitors were tested in three concentrations (0.2 nM, 10 nM, and 40 nM for **9** and 7 nM, 48 nM, and 164 nM for **DL76**) representing their IC_20_, IC_50_, and IC_80_, respectively. Substrate (p-tyramine) was used in six concentrations: 0.05 mM, 0.1 mM, 0.5 mM, 1 mM, 1.5 mM, and 2 mM. (**B**,**D**) Double-reciprocal (Lineweaver–Burk) plots from enzyme kinetic studies.

In order to determine the mode of hMAO B inhibition, compounds **9** and **DL76** were chosen and used in three concentrations corresponding to their IC_20_, IC_50_, and IC_80_ values (0.2 nM, 10 nM, and 40 nM for **9** and 7 nM, 48 nM, and 164 nM for **DL76**). Results from the kinetic experiment were used for determination of Michaelis–Menten curves (Figure 4A,C). Later, data were transformed using the Lineweaver–Burk equation to double reciprocal plot (lines from different concentrations of both compound **9** and **DL76** converged to the left of *y*-axis and above *x*-axis; Figure 4B,D). From Michaelis–Menten curves, V_max_ and K_M_ values were fitted (Table 2). For both compounds, V_max_ values decreased curvilinearly and K_M_ values increased curvilinearly with the increased inhibitor concentrations. The behavior of these parameters suggested the mixed mode inhibition of **9** and **DL76** [17,18]. This observation was further confirmed by a value of calculated constant α (α = 1.6 for **9** and α = 4.6 for **DL76**) by GraphPad Prism software from the nonlinear regression (see Supplementary Materials S2). According to Copeland [18,19], α > 1 is characteristic for the mixed mode of inhibition where an inhibitor is able to bind to both the free enzyme and the enzyme–substrate complex unequally and its affinity for binding to the free enzyme is higher (see Table S1, Supplementary Materials S2).

### 2.4. Human Monoamine Oxidase A Inhibitory Activity

Selected compounds (**DL76**, **6, 9, 10**, **12**, **16**, and **28**) were screened for hMAO A inhibition using Amplex Red^®^ Monoamine Oxidase kits [15]. Tested compounds showed a percentage of inihibition <25% at a concentration of 10 μM. Results indicated very weak or no inhibition of hMAO A by these compounds.

### 2.5. Toxicity and Neuroprotection Studies In Vitro

**Figure 5:**
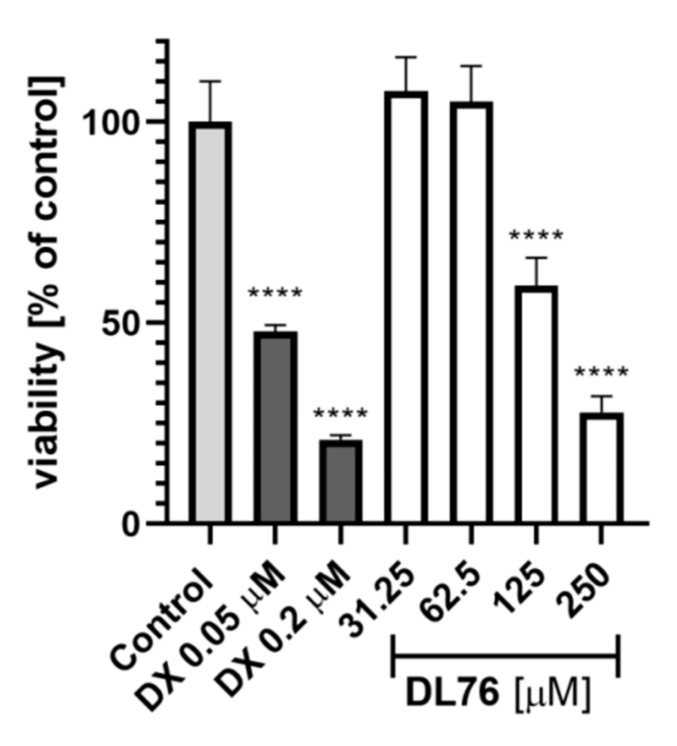
The effect of **DL76** and doxorubicin (DX) on HEK-293 viability: Statistical significance (**** *p* < 0.0001) was analyzed by GraphPadPrism™ 8 software using one-way ANOVA and Bonferroni’s multiple comparison posttest in comparison with control (1% DMSO in cell culture medium).

**Figure 6:**
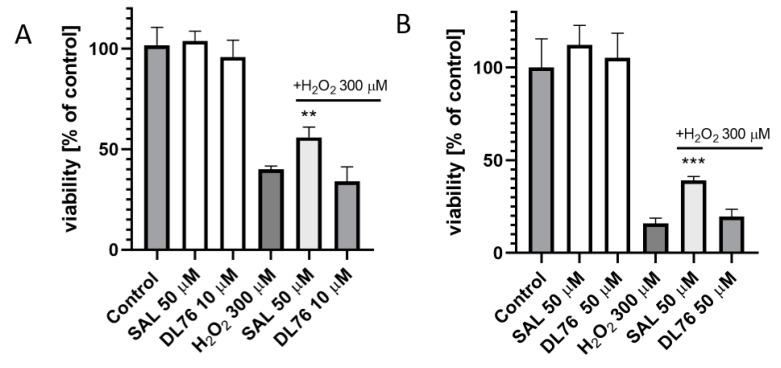
The effect of salsolinol (SAL) at 50 µM and **DL76** at 10 µM (**A**) or at 50 µM (**B**) on SH-SY5Y neuroblastoma cells viability damaged by 300 μM of H_2_O_2_ after 24 h of incubation: Statistical significance was set at *** *p* < 0.001 and ** *p* < 0.01 by GraphPadPrism™ 8 software using one-way ANOVA and Bonferroni’s multiple comparison posttest in comparison with the positive control H_2_O_2_ (300 μM).

For toxicity and neuroprotection studies, **DL76** was chosen as the most promising DTL. The human embryonic kidney (HEK-293) cell line was used for the estimation of safety of **DL76**. As shown in Figure 5, the statistically significant decrease of HEK-293 cell viability in the presence of **DL76** was observed only at the highest doses of 125 µM and 250 µM. The used reference cytostatic drug doxorubicin (DX) decreased cell viability <50% under the same conditions at very low concentrations of 0.2 and 0.05 µM. The neuroprotection effect of **DL76** was investigated in vitro in the model of neuronal damage. Oxidative damage was induced by very high toxic levels of hydrogen peroxide (H_2_O_2_; 300 µM), the popular cell model for PD research, in neuroblastoma SH-SY5Y cell line. Compound **DL76** was tested at two doses, 10 μM and 50 µM, within two independent experiments. Salsolinol (SAL) was used at a concentration of 50 µM as the reference compound with the proven neuroprotection activity [20]. **DL76** displayed no increase of cell survival either at 10 µM (Figure 6A) or 50 µM (Figure 6B), whereas SAL statistically significant increased cells viability in both experiments. However, the safety of **DL76** was confirmed here, as no toxic effect against SH-SY5Y cells was observed at both used concentrations 10 and 50 µM (Figure 6A,B).

### 2.6. Antiparkinsonian Activity in Haloperidol-Induced Catalepsy in Wistar Rats

Haloperidol (dopamine D_2_ antagonist)-induced catalepsy in rodents is a popular model for screening of antiparkinsonian activity of compounds [21]. Caused symptoms in animals are similar to the inability of PD patients to initiate movements. We used this test to preliminary evaluate antiparkinsonian activity of **DL76**. Haloperidol (0.63 mg/kg; s.c.) was administered to induce catalepsy in Wistar rats; 5 min later, **DL76** was added at doses of 25 or 50 mg/kg (i.p.). The reversal of catalepsy was tested for 6 min (3 times in 3 min intervals) after 1 h of haloperidol administration. As the reference compound was used **MSX-3**, the adenosine A_2A_ receptor antagonist with confirmed ability to reverse catalepsy mediated by D_1_ and D_2_ receptor antagonists [22]. To characterize the antiparkinsonian activity of **DL76**, two tests were performed: bar test and crossed-leg position test.

#### 2.6.1. Bar Test

Haloperidol-induced catalepsy was not reduced by **DL-76** administrated at a dose of 25 mg/kg body weight (Table 3). After administration of **DL-76** at a dose of 50 mg/kg body weight, low antiparkinsonian activity in the bar test was observed. The compound reduced the duration of catalepsy by 36.4% compared to the control group, which was obtained after administration of haloperidol (16.4 versus 25.8). This effect was weaker than the anti-Parkisonian activity of **MSX-3.**

#### 2.6.2. Crossed-Leg Position Test

The strongest, statistically significant antiparkinsonian activity was demonstrated by **DL-76** at a dose of 50 mg/kg body weight, which, compared to the control group, reduced the duration of haloperidol-induced catalepsy by 99.7% (0.053 versus 19.25) (Table 4). This effect was comparable to the antiparkinsonian activity of **MSX-3**.

The weaker, antiparkinsonian activity was demonstrated by **DL-76** administered at a dose of 25 mg/kg body weight. **DL76**, in comparison to the control group, reduced the duration of haloperidol-induced catalepsy by 54.5% (8.75 versus 19.25) (Table 4), but the effect was not statistically significant.

## 3. Materials and Methods

### 3.1. Chemistry

All reagents were purchased from commercial suppliers Alfa Aesar (Karlsruhe, Germany) or Sigma Aldrich (Darmstadt, Germany) and were used without further purification. TLC data were obtained with Merck silica gel 60F_254_ aluminum sheets with UV light detection and evaluation with Dragendorff’s reagent (solvent systems: methylene chloride: methanol 9:1 or methylene chloride). Melting points (m.p.) were determined on a MEL-TEMP II (LD Inc., Long Beach, CA, USA) melting point apparatus and are uncorrected. Mass spectra (LC/MS) were performed on Waters TQ Detector (Water Corporation., Milford, CT, USA) mass spectrometer. Retention times (t_R_) are given in minutes. The UPLC/MS purity of all final compounds was determined (%). All compounds (except **14**, **19**, and **26**) showed purity above 96%. ^1^H NMR and ^13^C NMR spectra were recorded in DMSO-*d*_6_ on a Mercury 300 MHz PFG spectrometer (Varian, Palo Alto, CA, USA), Avance III HD 400 MHz spectrometer (Bruker, Billerica, MA, USA), or FT-NMR 500 MHz spectrometer (Jeol Ltd., Akishima, Tokyo, Japan). Chemical shifts were expressed in parts per million (ppm) using the solvent signal as an internal standard. Data are reported in the following order: multiplicity (br, broad; d, dublet; m, multiplet; quin, quintet; s, singlet; and t, triplet), approximate coupling constants *J* expressed in Hertz (Hz), and number of protons. Elemental analyses were measured on analyzer Vario EL III 2 (Elementar, Langenselbold, Germany) and are within ±0.5% of the theoretical values (except carbon for **7** (±0.6%) and **28** (±0.7%).

4-*tert*-Butylphenoxyalkyl bromides (**4a**–**4d**) were synthesized according to the method described previously [14]. All of them are known and reported in Chemical Abstract Database:

1-(3-bromopropoxy)-4-*tert*-butylbenzene(**4a**):CAS3245-63-4; 1-(4-bromobutoxy)-4-*tert*-butylbenzene (**4b**): CAS53669-73-1;
1-(5-bromopentyloxy)-4-tert-butylbenzene (**4c**): CAS53669-74-2;
1-(6-bromohexyloxy)-4-tert-butylbenzene (**4d**): CAS53669-73-3.

Designed compounds **5**–**31** were synthesized according to the procedure described previously [12]. Briefly, to a proper 4-*tert*-butylphenylalkoxy bromide (2.5 mmol) in the mixture of C_2_H_5_OH (52.5 mL) and H_2_O (10 mL) and in the presence of K_2_CO_3_ (7.5 mmol) with the catalytic amount of KI was added a proper amine (5 mmol), and the solution was refluxed from 10 to 48 h. Then, C_2_H_5_OH was evaporated, and to the residue were added 50 mL of CH_2_Cl_2_ and 40 mL of water. The organic solution was washed with 50 mL of 1% HCl, evaporated to dryness, dissolved in 3% HCl followed by washing with (C_2_H_5_)_2_O, and neutralized with 5% NaOH. The final product was extracted with CH_2_Cl_2_, dried over Na_2_SO_4_, and evaporated. The oily residue was transformed into oxalic acid salt in absolute C_2_H_5_OH and precipitated (C_2_H_5_)_2_O. The solid was crystallized from C_2_H_5_OH.

*1-(3-(4-tert-Butylphenoxy)propyl)pyrrolidine hydrogen oxalate* (**5**). White solid, yield 27%, m.p. 147–149 °C, C_17_H_27_NO x C_2_H_2_O_4_ (MW = 351.43). ^1^H NMR (400 MHz, DMSO-*d*_6_) δ: 7.29 (d, *J* = 8.61 Hz, 2H), 6.86 (d, *J* = 8.61 Hz, 2H), 4.01 (t, *J* = 6.06 Hz, 2H), 3.20–3.31 (m, 6H), 2.05–2.14 (m, 2H), 1.93 (br s, 4H), 1.25 (s, 9H). ^13^C NMR (126 MHz, DMSO-*d*_6_) δ: 165.3, 156.5, 143.4, 126.5, 114.4, 65.3, 53.5, 51.9, 34.2, 31.8, 25.9, 23.2.LC-MS: purity 100% t_R_ = 5.27, (ESI) *m*/*z* [M + H]^+^ 262.24. Anal. calcd. for C_19_H_29_NO_5_: C, 64.93; H, 8.32; N, 3.99%. Found: C, 64.90; H, 8.35; N, 3.93%.

*1-(4-(4-tert-Butylphenoxy)butyl)piperidine hydrogen oxalate* (**6**). White solid, yield 52%, m.p. 150–152 °C, C_19_H_31_NO x C_2_H_2_O_4_ (MW = 379.48). ^1^H NMR (400 MHz, DMSO-*d*_6_) δ: 7.28 (d, *J* = 9.00 Hz, 2H), 6.85 (d, *J* = 8.61 Hz, 2H), 3.95 (t, *J* = 5.87 Hz, 2H), 2.95–3.31 (m, 6H), 1.64–1.88 (m, 8H), 1.52 (br. s., 2H), 1.25 (s, 9H). ^13^C NMR (101 MHz, DMSO-*d*_6_) δ: 165.2, 156.7, 143.2, 126.5, 114.4, 67.2, 56.1, 52.4, 40.9, 34.2, 31.8, 26.5, 23.0, 22.0, 20.8.LC-MS: purity 100% t_R_ = 5.69, (ESI) *m*/*z* [M + H]^+^ 290.22. Anal. calcd. for C_21_H_33_NO_5_: C, 66.46; H, 8.76; N, 3.69%. Found: C, 66.45; H, 8.48; N, 3.62%.

*1-(5-(4-tert-Butylphenoxy)pentyl)piperidine hydrogen oxalate* (**7**). White solid, yield 42%, m.p. 170–172 °C, C_20_H_33_NO x C_2_H_2_O_4_ (MW = 393.51). ^1^H NMR (400 MHz, DMSO-*d*_6_) δ: 7.28 (d, *J* = 8.61 Hz, 2H), 6.84 (d, *J* = 8.61 Hz, 2H), 3.93 (t, *J* = 6.06 Hz, 1H), 2.89–3.39 (m, 6H), 1.72 (br. s., 8H), 1.52 (br. s., 2H), 1.37–1.48 (m, 2H), 1.25 (s, 9H). ^13^C NMR (101 MHz, DMSO-*d*_6_) δ: 165.1, 156.8, 143.0, 126.5, 114.4, 67.5, 56.3, 52.4, 40.9, 34.2, 31.8, 28.7, 23.4, 23.3, 23.0, 22.0. LC-MS: purity 98.98% t_R_ = 5.98, (ESI) *m*/*z* [M + H]^+^ 304.18. Anal. calcd. for C_22_H_35_NO_5_: C, 67.14; H, 8.96; N, 3.56%. Found: C, 66.50; H, 8.71; N, 3.42%.

*1-(6-(4-tert-Butylphenoxy)hexyl)piperidine hydrogen oxalate* (**8**). White solid, yield 16%, m.p. 156–158 °C, C_21_H_35_NO x C_2_H_2_O_4_ (MW = 407.54). ^1^H NMR (400 MHz, DMSO-*d*_6_) δ: 7.27 (d, *J* = 9.00 Hz, 2H), 6.83 (d, *J* = 8.61 Hz, 2H), 3.92 (t, *J* = 6.26 Hz, 2H), 2.86–3.21 (m, 6H), 1.59–1.87 (m, 8H), 1.38–1.56 (m, 4H), 1.34 (d, *J* = 7.04 Hz, 2H), 1.25 (s, 9H). ^13^C NMR (101 MHz, DMSO-*d*_6_) δ: 165.3, 156.9, 143.0, 126.5, 114.3, 67.6, 56.3, 52.4, 40.9, 34.2, 31.8, 29.0, 26.4, 25.6, 23.6, 22.9, 22.0.LC-MS: purity 96.38% t_R_ = 6.39, (ESI) *m*/*z* [M + H]^+^ 318.20. Anal. calcd. for C_23_H_37_NO_5_: C, 67.78; H, 9.15; N, 3.44%. Found: C, 67.26; H, 9.13; N, 3.42%.

*1-(3-(4-tert-Butylphenoxy)propyl)-2-methylpiperidine hydrogen oxalate* (**9**). White solid, yield 19%, m.p. 114–116 °C, C_19_H_31_NO x C_2_H_2_O_4_ (MW = 379.48). ^1^H NMR (400 MHz, DMSO-*d*_6_) δ: 7.30 (d, *J* = 8.61 Hz, 2H), 6.86 (d, *J* = 9.00 Hz, 2H), 3.93–4.12 (m, 2H), 3.18–3.44 (m, 3H), 3.06–3.17 (m, 1H), 2.92–3.05 (m, 1H), 2.01–2.20 (m, 2H), 1.78–1.91 (m, 1H), 1.53–1.76 (m, 4H), 1.38–1.52 (m, 1H), 1.28 (d, *J* = 6.65 Hz, 3H), 1.25 (s, 9H).^13^C NMR (101 MHz, DMSO-*d*_6_) δ: 165.2, 156.5, 143.4, 126.5, 114.5, 65.4, 57.3, 49.3, 40.9, 34.2, 31.8, 23.4, 22.8. LC-MS: purity 100% t_R_ = 5.68, (ESI) *m*/*z* [M + H]^+^ 290.22. Anal. calcd. for C_21_H_33_NO_5_: C, 66.46; H, 8.76; N, 3.69%. Found: C, 66.29; H, 8.80; N, 3.64%.

*1-(4-(4-tert-Butylphenoxy)butyl)-2-methylpiperidine hydrogen oxalate* (**10**). White solid, yield 34%, m.p. 107–110 °C, C_20_H_33_NO x C_2_H_2_O_4_ (MW = 393.51). ^1^H NMR (400 MHz, DMSO-*d*_6_) δ: 7.29 (d, *J* = 9.00 Hz, 2H), 6.85 (d, *J* = 9.00 Hz, 2H), 3.95 (m, 2H), 3.27 (br.s., 2H), 3.08–3.19 (m, 1H), 2.91–3.08 (m, 2H), 1.68–1.87 (m, 7H), 1.39–1.67 (m, 3H), 1.20 – 1.33 (m, 12H).^13^C NMR (101 MHz, DMSO-*d*_6_) δ: 165.2, 156.7, 143.2, 126.5, 114.4, 67.3, 51.6, 34.2, 31.8, 26.5, 22.7, 20.2.LC-MS: purity 100% t_R_ = 5.94, (ESI) *m/z* [M + H]^+^ 304.24. Anal. calcd. for C_22_H_35_NO_5_: C, 67.14; H, 8.96; N, 3.56%. Found: C, 67.00; H, 8.95; N, 3.52%.

*1-(5-(4-tert-Butylphenoxy)pentyl)-2-methylpiperidine hydrogen oxalate* (**11**). White solid, yield 11%, m.p. 96–99 °C, C_21_H_35_NO x C_2_H_2_O_4_ (MW = 407.54). ^1^H NMR (400 MHz, DMSO-*d*_6_) δ: 7.28 (d, *J* = 6.81 Hz, 2H), 6.84 (d, *J* = 9.00 Hz, 2H), 3.94 (t, *J* = 6.26 Hz, 2H), 3.26 (br.s., 2H), 3.03–3.13 (m, 1H), 2.91–3.02 (m, 2H), 1.56–1.86 (m, 9H), 1.40–1.50 (m, 3H), 1.23–1.30 (m, 12H).^13^C NMR (101 MHz, DMSO-*d*_6_) δ: 165.2, 156.8, 143.0, 126.5, 114.4, 67.5, 51.7, 34.2, 31.8, 28.7, 23.4, 22.9.LC-MS: purity 96.49% t_R =_ 6.23, (ESI) *m*/*z* [M + H]^+^318.27. Anal. calcd. for C_23_H_37_NO_5_: C, 67.78; H, 9.15; N, 3.44%. Found: C, 67.50; H, 8.97; N, 3.41%.

*1-(3-(4-tert-Butylphenoxy)propyl)-3-methylpiperidine hydrogen oxalate* (**12**). White solid, yield 29%, m.p. 141–143 °C, C_19_H_31_NO x C_2_H_2_O_4_ (MW = 379.48). ^1^H NMR (500 MHz, DMSO-*d*_6_) δ: 7.24 (d, *J* = 8.88 Hz, 2H), 6.80 (d, *J* = 8.59 Hz, 2H), 3.95 (t, *J* = 5.87 Hz, 2H), 3.24–3.43 (m, 2H), 3.07 (t, *J* = 7.59 Hz, 2H), 2.63–2.77 (m, 1H), 2.37–2.45 (m, 1H), 2.00–2.16 (m, 2H), 1.78–1.90 (m, 1H), 1.61–1.77 (m, 3H), 1.20 (s, 9H), 0.94–1.10 (m, 1H), 0.85 (d, *J* = 6.59 Hz, 3H). ^13^C NMR (126 MHz, DMSO-*d*_6_) δ: 165.4, 156.6, 143.4, 126.6, 114.5, 65.5, 58.0, 54.0, 52.1, 34.3, 31.9, 30.6, 29.0, 24.1, 22.8, 19.1. LC-MS: purity 98.04% t_R_^=^ 5.75, (ESI) *m*/*z* [M + H]^+^ 290.22. Anal. calcd. for C_21_H_33_NO_5_: C, 66.46; H, 8.76; N, 3.69%. Found: C, 66.44; H, 8.42; N, 3.65%.

*1-(4-(4-tert-Butylphenoxy)butyl)-3-methylpiperidine hydrogen oxalate* (**13**). White solid, yield 53%, m.p. 148–150 °C, C_20_H_33_NO x C_2_H_2_O_4_ (MW = 393.51). ^1^H NMR (400 MHz, DMSO-*d*_6_) δ: 7.28 (d, *J* = 8.61 Hz, 2H), 6.85 (d, *J* = 8.61 Hz, 2H), 3.95 (t, *J* = 6.06 Hz, 2H), 3.24–3.45 (m, 2H), 2.94–3.11 (m, 2H), 2.67–2.78 (m, 2H), 1.62–1.97 (m, 8H), 1.25 (s, 9H), 1.06 (m, 1H), 0.89 (d, *J* = 6.65 Hz, 3H).^13^C NMR (101 MHz, DMSO-*d*_6_) δ: 165.2, 156.7, 143.1, 126.5, 114.4, 67.2, 57.9, 56.1, 51.9, 40.9, 34.2, 31.8, 30.6, 28.9, 26.5, 22.6, 20.8, 19.1.LC-MS: purity 99.46% t_R_ = 6.13, (ESI) *m*/*z* [M + H]^+^ 304.24. Anal. calcd. for C_22_H_35_NO_5_: C, 67.14; H, 8.96; N, 3.56%. Found: C, 66.86; H, 8.62; N, 3.51%.

*1-(5-(4-tert-Butylphenoxy)pentyl)-3-methylpiperidine hydrogen oxalate* (**14**). White solid, yield 8%, m.p. 179–181 °C, C_21_H_35_NO x C_2_H_2_O_4_ (MW = 407.54). ^1^H NMR (400 MHz, DMSO-*d*_6_) δ: 7.28 (d, *J* = 8.22 Hz, 2H), 6.84 (d, *J* = 7.83 Hz, 2H), 3.94 (br. s., 2H), 3.19–3.48 (m, 2H), 2.97 (br. s., 4H), 1.57–1.99 (m, 8H), 1.35–1.54 (m, 2H), 1.26 (br. s., 9H), 1.06 (d, *J* = 9.39 Hz, 1H), 0.69–0.97 (m, 3H). ^13^C NMR (101 MHz, DMSO-*d*_6_) δ: 165.0, 156.8, 143.1, 126.5, 114.4, 67.5, 58.0, 56.4, 52.0, 41.0, 34.2, 31.8, 30.6, 28.7, 23.5, 23.3, 19.1.LC-MS: purity 93.40% t_R_ = 6.40, (ESI) *m*/*z* [M + H]^+^ 318.20. Anal. calcd. for C_23_H_37_NO_5_: C, 67.78; H, 9.15; N, 3.44%. Found: C, 67.44; H, 8.90; N, 3.43%.

*1-(6-(4-tert-Butylphenoxy)hexyl)-3-methylpiperidine hydrogen oxalate* (**15**). White solid, yield 7%, m.p. 140–142 °C, C_22_H_37_NO x C_2_H_2_O_4_ (MW = 421.56). ^1^H NMR (400 MHz, DMSO-*d*_6_) δ: 7.27 (d, *J* = 8.61 Hz, 2H), 6.83 (d, *J* = 9.00 Hz, 2H), 3.92 (t, *J* = 6.46 Hz, 2H), 3.23–3.43 (m, 2H), 2.88–3.04 (m, 2H), 2.68–2.76 (m, 2H), 1.57–1.95 (m, 8H), 1.43 (quin, *J* = 7.34 Hz, 2H), 1.33 (quin, *J* = 7.04 Hz, 2H), 1.25 (s, 9H), 0.97–1.14 (m, 1H), 0.89 (d, *J* = 6.65 Hz, 3H). ^13^C NMR (101 MHz, DMSO-*d*_6_) δ: 165.2, 156.9, 143.0, 126.5, 114.3, 67.6, 57.8, 56.3, 51.9, 40.9, 34.2, 31.8, 30.6, 29.0, 28.9, 26.4, 25.6, 23.6, 22.6, 19.1. LC-MS: purity 100% t_R_ = 6.75, (ESI) *m*/*z* [M + H]^+^ 332.22. Anal. calcd. for C_24_H_39_NO_5_: C, 68.37; H, 9.32; N, 3.32%. Found: C, 68.14; H, 9.20; N, 3.28%.

*1-(3-(4-tert-Butylphenoxy)propyl)-3,3-dimethylpiperidine hydrogen oxalate* (**16**). White solid, yield 44%, m.p. 183–186 °C, C_20_H_33_NO x C_2_H_2_O_4_ (MW = 393.51). ^1^H NMR (400 MHz, DMSO-*d*_6_) δ: 7.30 (d, *J* = 8.61 Hz, 2H), 6.85 (d, *J* = 8.61 Hz, 2H), 3.99 (t, *J* = 5.67 Hz, 2H), 2.79–3.30 (m, 6H), 2.10 (br. s., 2H), 1.74 (br. s., 2H), 1.36 (br. s., 2H), 1.25 (s, 9H), 1.00 (s, 6H).^13^C NMR (101 MHz, DMSO-*d*_6_) δ: 164.7, 156.5, 143.4, 126.5, 114.4, 65.6, 62.1, 55.0, 52.8, 40.9, 35.3, 34.2, 31.8, 30.9, 24.2, 20.2. LC-MS: purity 100% t_R_ = 6.02, (ESI) *m*/*z* [M + H]^+^ 304.24. Anal. calcd. for C_22_H_35_NO_5_: C, 67.14; H, 8.96; N, 3.56%. Found: C, 67.19; H, 8.50; N, 3.51%.

*1-(4-(4-tert-Butylphenoxy)butyl)-3,3-dimethylpiperidine hydrogen oxalate* (**17**). White solid, yield 23%, m.p. 140–142 °C, C_21_H_35_NO x C_2_H_2_O_4_ (MW = 407.54).^1^H NMR (400 MHz, DMSO-*d*_6_) δ: 7.28 (d, *J* = 8.61 Hz, 2H), 6.85 (d, *J* = 8.61 Hz, 2H), 3.95 (t, *J* = 5.87 Hz, 2H), 2.91–3.20 (m, 4H), 2.80 (br. s., 2H), 1.63–1.90 (m, 6H), 1.37 (d, *J* = 4.70 Hz, 2H), 1.25 (s, 9H), 0.99 (s, 6H).^13^C NMR (101 MHz, DMSO-*d*_6_) δ: 165.0, 156.7, 143.1, 126.5, 114.4, 67.2, 61.8, 57.1, 52.5, 40.9, 35.2, 34.2, 31.8, 30.9, 26.6, 20.8, 20.1.LC-MS: purity 100% t_R_ = 6.29, (ESI) *m*/*z* [M + H]^+^318.27. Anal. calcd. for C_23_H_37_NO_5_: C, 67.78; H, 9.15; N, 3.44%. Found: C, 67.57; H, 9.03; N, 3.39%.

*1-(5-(4-tert-Butylphenoxy)pentyl)-3,3-dimethylpiperidine hydrogen oxalate* (**18**). White solid, yield 40%, m.p. 150–153 °C, C_22_H_37_NO x C_2_H_2_O_4_ (MW = 421.56). ^1^H NMR (400 MHz, DMSO-*d*_6_) δ: 7.28 (d, *J* = 8.61 Hz, 2H), 6.84 (d, *J* = 9.00 Hz, 2H), 3.93 (t, *J* = 6.26 Hz, 2H), 2.88–3.24 (m, 4H), 2.81 (br. s., 2H), 1.61–1.83 (m, 6H), 1.30–1.45 (m, 4H), 1.25 (s, 9H), 0.99 (s, 6H). ^13^C NMR (101 MHz, DMSO-*d*_6_) δ: 165.0, 156.8, 143.0, 126.5, 114.4, 67.4, 61.8, 57.3, 52.5, 40.9, 35.2, 34.2, 31.8, 30.9, 28.7, 23.4, 23.3, 20.0.LC-MS: purity 96.27% t_R_ = 6.58, (ESI) *m*/*z* [M + H]^+^ 332.29. Anal. calcd. for C_24_H_39_NO_5_: C, 68.37; H, 9.32; N, 3.32%. Found: C, 67.94; H, 8.99; N, 3.24%.

*1-(6-(4-tert-Butylphenoxy)hexyl)-3,3-dimethylpiperidine hydrogen oxalate* (**19**). White solid, yield 21%, m.p. 122–125 °C, C_23_H_39_NO x C_2_H_2_O_4_ (MW = 435.59). ^1^H NMR (400 MHz, DMSO-*d*_6_) δ: 7.27 (d, *J* = 8.61 Hz, 2H), 6.83 (d, *J* = 8.61 Hz, 2H), 3.83–4.01 (m, *J* = 6.46, 6.46 Hz, 2H), 2.75–3.13 (m, 6H), 1.57–1.83 (m, 6H), 1.29–1.54 (m, 6H), 1.25 (s, 9H), 0.99 (s, 6H). ^13^C NMR (101 MHz, DMSO-*d*_6_) δ: 165.1, 156.9, 143.0, 126.5, 114.3, 67.6, 61.7, 57.3, 52.4, 40.9, 35.2, 34.2, 31.8, 30.9, 29.0, 26.4, 25.6, 23.5, 20.0. LC-MS: purity 94.18% t_R_ = 6.97, (ESI) *m*/*z* [M + H]^+^ 346.25. Anal. calcd. for C_25_H_41_NO_5_: C, 68.93; H, 9.46; N, 3.22%. Found: C, 68.80; H, 9.26; N, 3.22%.

*1-(3-(4-tert-Butylphenoxy)propyl)-3,5-dimethylpiperidine hydrogen oxalate* (**20**). White solid, yield 43%, m.p. 143–145 °C, C_20_H_33_NO x C_2_H_2_O_4_ (MW = 393.51). ^1^H NMR (400 MHz, DMSO-*d*_6_) δ: 7.29 (d, *J* = 9.00 Hz, 2H), 6.85 (d, *J* = 8.61 Hz, 2H), 4.00 (t, *J* = 5.67 Hz, 2H), 3.34 (d, *J* = 10.17 Hz, 2H), 2.97–3.23 (m, 2H), 2.40 (t, *J* = 11.93 Hz, 2H), 2.02–2.23 (m, 2H), 1.91 (d, *J* = 3.52 Hz, 2H), 1.73 (d, *J* = 12.91 Hz, 1H), 1.33–1.49 (m, 1H), 1.25 (s, 9H), 0.99 (d, *J* = 6.65 Hz, 6H). ^13^C NMR (101 MHz, DMSO-*d*_6_) δ: 165.0, 156.5, 143.4, 126.5, 114.4, 65.5, 57.6, 53.9, 34.2, 31.8, 28.8, 24.1, 18.9.LC-MS: purity 15.62% t_R_ = 6.08, (ESI) *m*/*z* [M + H]^+^ 304.24 and purity 84.38% t_R_ = 6.15, (ESI) *m*/*z* [M + H]^+^ 304.24. Anal. calcd. for C_22_H_35_NO_5_: C, 67.14; H, 8.96; N, 3.56%. Found: C, 67.03; H, 9.14; N, 3.52%.

*1-(4-(4-tert-Butylphenoxy)butyl)-3,5-dimethylpiperidine hydrogen oxalate* (**21**). White solid, yield 39%, m.p. 170–172 °C, C_21_H_35_NO x C_2_H_2_O_4_ (MW = 407.54). ^1^H NMR (400 MHz, DMSO-*d*_6_) δ: 7.29 (d, *J* = 8.61 Hz, 2H), 6.85 (d, *J* = 8.61 Hz, 2H), 3.96 (t, *J* = 6.06 Hz, 2H), 3.31 (d, *J* = 9.78 Hz, 2H), 2.90–3.09 (m, 2H), 2.37 (t, *J* = 11.93 Hz, 2H), 1.61–2.02 (m, 7H), 1.13–1.44 (s, 10H), 0.87–1.04 (m, 6H). ^13^C NMR (101 MHz, DMSO-d_6_) δ:165.0, 156.7, 143.2, 126.5, 114.4, 67.2, 57.4, 56.1, 34.2, 31.8, 28.7, 26.5, 20.8, 18.9. LC-MS: purity 11.21% t_R_ = 6.31, (ESI) *m*/*z* [M + H]^+^ 318.46 and purity 88.79% t_R_ = 6.37, (ESI) *m*/*z* [M + H]^+^ 318.46. Anal. calcd. for C_23_H_37_NO_5_: C, 67.78; H, 9.15; N, 3.44%. Found: C, 68.14; H, 8.87; N, 3.40%.

*1-(5-(4-tert-Butylphenoxy)pentyl)-3,5- dimethylpiperidine hydrogen oxalate* (**22**). White solid, yield 31%, m.p. 175–178 °C, C_22_H_37_NO x C_2_H_2_O_4_ (MW = 421.56). ^1^H NMR (400 MHz, DMSO-*d*_6_) δ: 7.28 (d, *J* = 8.61 Hz, 2H), 6.84 (d, *J* = 8.61 Hz, 2H), 3.94 (t, *J* = 6.06 Hz, 2H), 3.31 (d, *J* = 10.56 Hz, 2H), 2.80–3.06 (m, 2H), 2.36 (t, *J* = 11.74 Hz, 2H), 1.59–2.12 (m, 7H), 1.15–1.52 (m, 12H), 0.83–0.99 (m, 6H). ^13^C NMR (101 MHz, DMSO-*d*_6_) δ: 164.9, 156.8, 143.1, 126.5, 114.4, 67.4, 57.5, 34.2, 31.8, 28.7, 23.5, 23.3, 18.9.LC-MS: purity 6.64% t_R_ = 6.73, (ESI) *m*/*z* [M + H]^+^ 332.29 and purity 93.36% t_R_ = 6.78, (ESI) *m*/*z* [M + H]^+^ 332.29. Anal. calcd. for C_24_H_39_NO_5_: C, 68.37; H, 9.32; N, 3.32%. Found: C, 68.25; H, 9.06; N, 3.30%.

*1-(6-(4-tert-Butylphenoxy)hexyl)-3,5- dimethylpiperidine hydrogen oxalate* (**23**). White solid, yield 43%, m.p. 143–145 °C, C_23_H_39_NO x C_2_H_2_O_4_ (MW = 345.56). ^1^H NMR (400 MHz, DMSO-*d*_6_) δ: 7.28 (d, *J* = 8.61 Hz, 2H), 6.84 (d, *J* = 8.61 Hz, 2H), 3.93 (t, *J* = 6.26 Hz, 2H), 3.30 (d, *J* = 10.17 Hz, 2H), 2.81–3.04 (m, 2H), 2.36 (t, *J* = 11.93 Hz, 2H), 1.88 (br. s., 2H), 1.60–1.78 (m, 5H), 1.20–1.53 (m, 14H), 0.89 (d, *J* = 6.65 Hz, 6H). ^13^C NMR (101 MHz, DMSO-*d*_6_) δ: 164.9, 156.9, 143.0, 126.5, 114.4, 67.6, 57.5, 34.2, 31.8, 28.9, 26.4, 25.6, 23.7, 18.9.LC-MS: purity 5.30% t_R_ = 6.96, (ESI) *m*/*z* [M + H]^+^ 346.31 and purity 94.70% t_R_ = 7.00, (ESI) *m*/*z* [M + H]^+^ 346.31. Anal. calcd. for C_25_H_41_NO_5_: C, 68.93; H, 9.49; N, 3.22%. Found: C, 68.74; H, 9.64; N, 3.19%.

*1-(3-(4-tert-Butylphenoxy)propyl)-4-methylpiperidine hydrogen oxalate* (**24**). White solid, yield 29%, m.p. 158–160 °C, C_19_H_31_NO x C_2_H_2_O_4_ (MW = 379.48). ^1^H NMR (500 MHz, DMSO-*d*_6_) δ: 7.25 (d, *J* = 8.88 Hz, 2H), 6.81 (d, *J* = 8.59 Hz, 2H), 3.95 (t, *J* = 6.01 Hz, 8H), 3.35 (d, *J* = 11.74 Hz, 2H), 3.02–3.15 (m, 2H), 2.82 (t, *J* = 11.60 Hz, 2H), 2.00–2.11 (m, 2H), 1.72 (d, *J* = 13.17 Hz, 2H), 1.57 (d, *J* = 6.01 Hz, 1H), 1.29–1.43 (m, 2H), 1.20 (s, 9H), 0.88 (d, *J* = 6.59 Hz, 3H). ^13^C NMR (126 MHz, DMSO-*d*_6_) δ: 165.2, 156.5, 143.4, 126.6, 114.5, 65.5, 53.8, 53.7, 52.2, 52.1, 52.1, 52.1, 52.1, 34.3, 31.9, 31.2, 31.2, 31.1, 31.1, 31.1, 31.1, 31.0, 28.5, 24.2, 21.4, 21.4.LC-MS: purity 98.17% t_R_ = 5.84, (ESI) *m*/*z* [M + H]^+^ 290.22. Anal. calcd. for C_21_H_33_NO_5_: C, 66.46; H, 8.76; N, 3.69%. Found: C, 65.97; H, 8.46; N, 3.63%.

*1-(4-(4-tert-Butylphenoxy)butyl)-4-methylpiperidine hydrogen oxalate* (**25**). White solid, yield 47%, m.p. 155–157 °C, C_20_H_33_NO x C_2_H_2_O_4_ (MW = 393.51). ^1^H NMR (400 MHz, DMSO-*d*_6_) δ: 7.28 (d, *J* = 9.00 Hz, 2H), 6.85 (d, *J* = 9.00 Hz, 2H), 3.95 (t, *J* = 5.87 Hz, 2H), 3.37 (d, *J* = 11.74 Hz, 2H), 2.97–3.13 (m, 2H), 2.84 (t, *J* = 11.54 Hz, 2H), 1.67–1.90 (m, 6H), 1.61 (d, *J* = 5.87 Hz, 1H), 1.33–1.50 (m, 2H), 1.25 (s, 9H), 0.91 (d, *J* = 6.65 Hz, 3H). ^13^C NMR (101 MHz, DMSO-*d*_6_) δ: 165.2, 156.7, 143.1, 126.5, 114.4, 67.2, 55.8, 51.8, 40.9, 34.2, 31.8, 30.9, 28.5, 26.5, 21.3, 20.9. LC-MS: purity 99.02% t_R_ = 6.07, (ESI) *m*/*z* [M + H]^+^ 304.24. Anal. calcd. for C_22_H_35_NO_5_: C, 67.14; H, 8.96; N, 3.56%. Found: C, 67.41; H, 8.48; N, 3.56%.

*1-(5-(4-tert-Butylphenoxy)pentyl)-4-methylpiperidine hydrogen oxalate* (**26**). White solid, yield 8%, m.p. 167–170 °C, C_21_H_35_NO x C_2_H_2_O_4_ (MW = 407.54). ^1^H NMR (400 MHz, DMSO-*d*_6_) δ: 7.28 (d, *J* = 8.61 Hz, 2H), 6.84 (d, *J* = 8.61 Hz, 2H), 3.94 (t, *J* = 6.26 Hz, 2H), 3.31–3.41 (m, 2H), 2.93–3.03 (m, 2H), 2.77–2.87 (m, 2H), 1.64–1.82 (m, 6H), 1.54–1.64 (m, 1H), 1.32–1.48 (m, 4H), 1.20–1.31 (m, 9H), 0.92 (d, *J* = 6.26 Hz, 3H). ^13^C NMR (101 MHz, DMSO-*d*_6_) δ: 165.0, 156.8, 143.0, 126.5, 114.4, 67.4, 40.9, 34.2, 31.8, 28.7, 23.6, 23.3.LC-MS: purity 92.34% t_R_ = 6.34, (ESI) *m*/*z* [M + H]^+^ 318.20. Anal. calcd. for C_23_H_37_NO_5_: C, 67.78; H, 9.15; N, 3.44%. Found: C, 67.65; H, 8.81; N, 3.42.

*1-(6-(4-tert-Butylphenoxy)hexyl)-4-methylpiperidine hydrogen oxalate* (**27**). White solid, yield 12%, m.p. 136–139 °C, C_22_H_37_NO x C_2_H_2_O_4_ (MW = 421.56). ^1^H NMR (400 MHz, DMSO-*d*_6_) δ: 7.27 (d, *J* = 8.61 Hz, 2H), 6.83 (d, *J* = 9.00 Hz, 2H), 3.92 (t, *J* = 6.46 Hz, 2H), 3.36 (d, *J* = 11.74 Hz, 2H), 2.89–3.03 (m, 2H), 2.76–2.87 (m, 2H), 1.54–1.82 (m, 7H), 1.29–1.48 (m, 6H), 1.25 (s, 9H), 0.91 (d, *J* = 6.26 Hz, 3H). ^13^C NMR (101 MHz, DMSO-*d*_6_) δ: 165.3, 156.9, 143.0, 126.5, 114.3, 67.6, 56.0, 51.8, 40.9, 34.2, 31.8, 30.9, 29.0, 28.5, 26.4, 25.6, 23.7, 21.3. LC-MS: purity 98.49% t_R_ = 6.77, (ESI) *m*/*z* [M + H]^+^ 332.22. Anal. calcd. for C_24_H_39_NO_5_: C, 68.37; H, 9.32; N, 3.32%. Found: C, 68.51; H, 9.40; N, 3.30%.

*1-(3-(4-tert-Butylphenoxy)propyl)-azepane hydrogen oxalate* (**28**). White solid, yield 32%, m.p. 138–140 °C, C_19_H_31_NO x C_2_H_2_O_4_ (MW = 379.48). ^1^H NMR (500 MHz, DMSO-*d*_6_) δ: 7.24 (d, *J* = 3.15 Hz, 2H), 6.81 (d, *J* = 2.86 Hz, 2H), 3.95 (br. s., 2H), 2.88–3.47 (m, 6H), 2.08 (br. s., 2H), 1.76 (br. s., 4H), 1.56 (br. s., 4H), 1.20 (br. s., 9H). ^13^C NMR (126 MHz, DMSO-*d*_6_) δ: 165.5, 156.6, 143.4, 126.6, 114.5, 65.5, 54.3, 54.1, 34.3, 31.9, 26.6, 24.4, 23.6. LC-MS: purity 99.17% t_R_ = 5.82, (ESI) *m*/*z* [M + H]^+^ 290.22. Anal. calcd. for C_21_H_33_NO_5_: C, 66.46; H, 8.76; N, 3.69%. Found: C, 65.74; H, 8.41; N, 3.65%.

*1-(4-(4-tert-Butylphenoxy)butyl)-azepane hydrogen oxalate* (**29**). White solid, yield 49%, m.p. 149–151 °C, C_20_H_33_NO x C_2_H_2_O_4_ (MW = 393.51). ^1^H NMR (400 MHz, DMSO-d_6_) δ: 7.28 (d, *J* = 8.22 Hz, 2H), 6.85 (d, *J* = 7.82 Hz, 2H), 3.95 (t, *J* = 5.67 Hz, 2H), 3.16–3.32 (m, 4H), 3.11 (d, *J* = 7.04 Hz, 2H), 1.68–1.89 (m, 8H), 1.60 (br. s., 4H), 1.25 (s, 9H). ^13^C NMR (101 MHz, DMSO-*d*_6_) δ: 165.3, 156.7, 143.1, 126.5, 114.4, 67.2, 56.4, 53.9, 40.9, 34.2, 31.8, 26.6, 26.5, 23.5, 21.2. LC-MS: purity 99.24% t_R_ = 6.01, (ESI) *m*/*z* [M + H]^+^ 304.24. Anal. calcd. for C_22_H_35_NO_5_: C, 67.14; H, 8.96; N, 3.56%. Found: C, 67.40; H, 8.51; N, 3.55%.

*1-(5-(4-tert-Butylphenoxy)pentyl)-azepane hydrogen oxalate* (**30**). White solid, yield 38%, m.p. 151–153 °C, C_21_H_35_NO x C_2_H_2_O_4_ (MW = 407.53). ^1^H NMR (400 MHz, DMSO-*d*_6_) δ: 7.28 (d, *J* = 8.61 Hz, 2H), 6.84 (d, *J* = 9.00 Hz, 2H), 3.93 (t, *J* = 6.26 Hz, 2H), 3.13–3.32 (m, 4H), 2.94–3.08 (m, 2H), 1.79 (br. s., 4H), 1.66–1.75 (m, 4H), 1.54–1.64 (m, 4H), 1.43 (d, *J* = 7.43 Hz, 2H), 1.25 (s, 9H). ^13^C NMR (101 MHz, DMSO-*d*_6_) δ: 165.3, 156.8, 143.0, 126.5, 114.4, 67.5, 56.6, 53.9, 40.9, 34.2, 31.8, 28.7, 26.6, 23.8, 23.4, 23.3. LC-MS: purity 100% t_R_ = 6.27, (ESI) *m*/*z* [M + H]^+^ 366.25. Anal. calcd. for C_23_H_37_NO_5_: C, 67.78; H, 9.15; N, 3.44%. Found: C, 67.47; H, 8.75; N, 3.39%.

*1-(6-(4-tert-Butylphenoxy)hexyl)-azepane hydrogen oxalate* (**31**). White solid, yield 5%, m.p. 130–132 °C, C_22_H_37_NO x C_2_H_2_O_4_ (MW = 421.56). ^1^H NMR (400 MHz, DMSO-*d*_6_) δ: 7.27 (d, *J* = 8.61 Hz, 2H), 6.83 (d, *J* = 8.61 Hz, 2H), 3.92 (t, *J* = 6.46 Hz, 2H), 3.16–3.23 (m, 4H), 2.98–3.04 (m, 2H), 1.58–1.82 (m, 12H), 1.28–1.47 (m, 4H), 1.25 (s, 9H). ^13^C NMR (101 MHz, DMSO-*d*_6_) δ: 165.3, 156.8, 143.0, 126.5, 114.3, 67.6, 56.7, 53.9, 40.9, 34.2, 31.8, 29.0, 26.6, 26.4, 25.6, 24.0, 23.4. LC-MS: purity 97.93% t_R_ = 6.79, (ESI) *m*/*z* [M + H]^+^ 332.22. Anal. calcd. for C_24_H_39_NO_5_: C, 68.37; H, 9.32; N, 3.32%. Found: C, 68.46; H, 8.98; N, 3.32%.

### 3.2. Biological Studies In Vitro

#### 3.2.1. Affinity for Human Histamine H_3_ Receptor

The radioligand displacement binding assay was carried out in HEK-293 cells stably expressing the recombinant hH_3_R as described by Kottke et al. [23] with slight modifications [14]. [^3^H]*N*^α^-methylhistamine was used as radiolabeled tracer (2 nM, K_D_ = 3.08). Obtained data from at least three experiments (in at least duplicates) were analyzed with GraphPad Prism 6.1 (San Diego, CA, USA) using nonlinear regression (one-site competition on logarithmic scale), and K_i_ values were transformed from IC_50_ according to Cheng-Prusoff [24]. Statistical analysis was performed on −log K_i_ values. Mean values and 95% confidence intervals were converted to nanomolar concentrations.

#### 3.2.2. Human Monoamine Oxidase Inhibitory Activity

##### General Method for Determining Activity Against MAO Isoforms

Inhibitory potency of compounds on MAO isoenzymes were carried out by a fluorometric method with a commercial Amplex™ Red Monoamine Oxidase Assay Kit (ThermoFisher Scientific A12214, Waltham, MA, USA) as described previously [12,25]. For all tests, recombinant human MAO B and MAO A (from Sigma AldrichM7441 and M7316, Darmstadt, Germany) were used. Inhibitors’ activity was measured in the presence of p-tyramine (200 μM). In all experiments, reference inhibitors in the concentrations that fully inhibited the MAO isoform were included. Reference inhibitors for MAO-B were pargyline 10 μM, rasagiline 1 μM, and safinamide 1 μM and, for MAO-A, was clorgyline 1 μM.

##### Screening and Determination of IC_50_

Firstly, inhibitors’ activity was measured in a concentration of 1 μM. The results were normalized (no inhibition = 0% and fully inhibited enzyme 100%). For compounds that inhibited the enzyme by more than 50%, further studies were conducted to obtain IC_50_ from concentration–response curves. All calculations were made in Microsoft Excel and GraphPadPrism software. All experiments were performed in duplicate, and data are expressed as mean ± SEM of 2–5 independent experiments.

##### Reversibility Studies

Reversibility of MAO B inhibitors was tested as described previously [12] with slight modification. Two experiments were performed simultaneously. In the first experiment, inhibitors (in concentrations corresponding to their IC_80_ values) were incubated with the enzyme and p-tyramine (10 μM) for 22 min (measuring fluorescence every two minutes). Next, the concentration of the substrate was increased to 1 mM and the fluorescence was measured every 5 min for 5 h. In the second experiment, inhibitors and enzyme were preincubated for 30 min in room temperature before addition of 10 μM p-tyramine; then, continuation of the experiment was carried out identically as the first.

##### Kinetic Studies

The mode of the inhibition was tested according to the standard procedure described in Reversibility Studies using different concentrations of the substrate [12,26]. Inhibitors (compound **9** and **DL76**) were used in three concentrations corresponding to their IC_20_, IC_50_, and IC_80_ values. Substrate was used in six concentrations: 0.05 nM, 0.1 mM, 0.5 mM, 1 mM, 1.5 mM, and 2 mM. After the experiment, velocities were calculated and put on the graph (*y*-axis) against the concentration of the substrate (*x*-axis). From the Michaelis–Menten plot V_max_, K_M_, and α values were calculated for different concentrations of the inhibitor. Double-reciprocal plot (Lineweaver–Burk plot) were prepared to display the data.

#### 3.2.3. Toxicity and Neuroprotection Evaluation In Vitro

##### Cell Lines

SH-SY5Y CRL-2266™ neuroblastoma cell line was purchased directly from American Type Culture Collection (Manassas, VG, USA) and were cultured as described previously [20]. Human embryonic kidney HEK-293 cell line (ATCC CRL-1573) was kindly donated by Prof. Dr Christa Müller (Pharmaceutical Institute, Pharmaceutical Chemistry I, University of Bonn, Germany). The cells were cultured as described previously [27].

##### Toxicity Studies

The HEK-293 cells were seeded at a concentration of 1.5 × 10^4^ cells/well in 200 μL culture medium and incubated for 24 h at 37 °C and 5% CO_2_. Next, the cytostatic reference doxorubicin (DX) or **DL76** dissolved in DMSO were added at various concentrations into microplate (total concentration of DMSO in media was 1%) and incubated for 48 h at 37 °C and 5% CO_2_. Then, 25 μL EZ4U labelling mixture (Biomedica, Vienna, Austria) was added and the cells were incubated for 2 h. The spectrophotometric absorbance of the samples was measured using a microplate reader (EnSpire, PerkinElmer, Waltham, MA, USA) at 492 nm. All measurements were performed in triplicate, and results are shown as mean ± SD.

##### Neuroprotection Studies

SH-SY5Y cells were seeded in microplate at a concentration of 2.5 × 10^4^ cells/well in 100 μL culture medium and cultured for 24 h at 37 C and 5% CO_2_ to reach 70% confluence. The cells were preincubated first for 1 h with **DL76** (10 and 50 µM) or with the reference neuroprotectant salsolinol. (R,S)-salsolinol (purity ≥ 99%) was obtained from Cayman Chemical (Ann Arbor, MI, USA). Next, H_2_O_2_ was added at final concentration 300 μM and the cells were placed into the incubator. After 24 h of compounds co-incubation with H_2_O_2_, the CellTiter 96^®^AQueous Non-Radioactive Cell Proliferation Assay (MTS) labeling mixture was added to each well, and the microplates were incubated under the same conditions for 5 h. The absorbance was measured using a microplate reader EnSpire (PerkinElmer, Waltham, MA USA) at 490 nm. All measurements were performed in triplicate, and results are shown as mean ± SD.

### 3.3. Antiparkinsonian Activity in Haloperidol-Induced Catalepsy In Vivo

#### 3.3.1. Animals

The experiments were carried out on male rats Wistar (180–220 g). Animals were housed in plastic cages in room at a constant temperature of 20 ± 2 °C with natural light–dark cycles. The animals had free access to standard pellet diet and water and were used after a minimum of 3 days of acclimatization to the housing conditions. Control and experimental groups consisted of 6 animals each. All experimental procedures were performed according to the European Union Directive of 22 September 2010 (2010/63/EU) and Polish legislation concerning animal care and use and was approved by the Local Ethics Committee for Experiments on Animals in Kraków, Poland (Resolution No. 70/2014, approval date: 20 May 2014). The examined compound was administered as the suspension in 0.5% methylcellulose in constant volume of 10 mL/kg.

#### 3.3.2. Drugs

Haloperidol was purchased from Sigma Aldrich (Darmstadt, Germany). MSX-3 ((E)-phosphoric acid mono-[3-[8-[2-(3-methoxyphenyl)vinyl]-7-methyl-2,6-dioxo-1-prop-2-ynyl-1,2,6,7-tetrahydro-purin-3-yl]propyl] ester disodium salt) was synthesized in the laboratory of Pharmaceutical Institute, Pharmaceutical Chemistry I, University of Bonn, Germany and donated by Prof. Dr Christa Müller.

#### 3.3.3. Statistical Analysis

The data are expressed as the means ± SEM. All statistical calculations were carried out with the GraphPad Prism 5 program. The data were evaluated by one-way analysis of variance (ANOVA) followed by Duncan test; *p* < 0.05 was considered significant.

#### 3.3.4. Determination of Antiparkinsonian Activity in Catalepsy Tests

Antiparkinsonian activity in catalepsy tests was performed as described in [28]. Haloperidol was administered s.c. at a dose of 0.63 mg/kg, which in 100% of control animals caused catalepsy. Tested compounds were administered i.p. at the doses 50 and 25 mg/kg body weight, 5 min after haloperidol injection. After 60 min from the injection of haloperidol, to assess the occurrence of catalepsy, the animals were placed in a forced position and the time they remained in this position was measured. Two tests were performed to determine the antiparkinsonian activity: crossed leg position test and bar test. In the first test, the animals were put on the hind paws behind the front (forced position); in the second, the animals were supported on a wooden block (the front paws were placed on a block suspended 10 cm above the ground). Then, the time that the animals remain is measured in a forced position (time was measured to a maximum of 60 s). Observation was carried out 3 times in 3-min intervals. The shortening of the time of catalepsy to the control group was adopted for the antiparkinsonian activity.

## 4. Conclusions

In summary, in this study, new potential DTL for PD have been designed and synthesized. As the lead structure, **DL76** was chosen, the compound with proven high hH_3_R affinity (K_i_ = 22 nM [13] in CHO K1 cells and K_i_ = 38 nM in HEK 293 cells) and hMAO B inhibitory activity in vitro (IC_50_ = 48 nM). The introduced modifications were aimed at assessing the influence of cyclic amines and the length of the alkyl chain on hH_3_R affinity and hMAO B inhibition. Most compounds showed nanomolar range affinities for hH_3_R. The significant increase in the inhibitory effect for hMAO B occurred for pyrrolidine (**5**) and 2-methylpiperidine (**9**) derivatives. These results confirmed our previous observations concerning 4-*tert*-amylphenoxy derivatives [12] where such compounds were among the most potent hMAO B inhibitors.

In vitro toxicity studies with **DL76** in the HEK293 cells and neuroblastoma SH-SY5Y cells did not show risk of toxicity at the dose of 50 μM of this compound. However, no neuroprotection effect of **DL76** against very high toxic levels of hydrogen peroxide (300 µM) in neuroblastoma SH-SY5Y cells was observed.

Conducted in vivo studies showed that tested **DL76** caused just statistically significant antiparkinsonian activity in the crossed-leg position test. The tested compound, at the dose of 50 mg/kg body weight, practically completely reduced the duration of catalepsy, whereas in the bar test at this dose, a low positive effect was observed. Moreover, **DL76** did not show any cataleptic effects.

Considering the dual functional profile, the most valuable compound proved to be **DL76** with balanced activity against both biological targets (hH_3_R K_i_ = 38 nM and hMAO B IC_50_ = 48 nM). Structural modification in DTL is not easy as it requires optimization towards both targets. By direct exchange of a cyclic amine, it was possible to obtain a very potent hMAO B inhibitor, compound **5** with the IC_50_ of 2.7 nM. However, this compound proved to be only a moderate hH_3_R ligand (K_i_ = 371 nM). Generally, very potent hMAO B inhibitors (**5**, **9**, and **28**) showed higher strength to inhibit hMAO B than to block hH_3_R. Thus, it seems that, in this class of compounds, a cyclic amine moiety in the western part of a molecule plays the very important role in the interaction with hMAO B, but further studies should be performed to confirm it. However, the presented compounds are promising starting materials in further search for new active DTL for PD.

## Figures and Tables

**Table 1:**
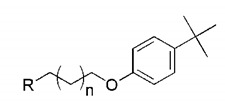
Human histamine H_3_ receptor affinity and human MAO B inhibition of tested compounds **5–31** and **DL76**.

Compounds	R	n	H_3_R ^a^ *K_i_* (nM) (95%CI)	MAO B ^b^ IC_50_ (nM) (%Inh.) ^c^	MAO A ^b^ IC_50_ (nM) (%Inh.) ^d^
**5**	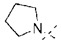	1	371 (136, 1009)	2.7 ± 0.4	nt ^e^
**DL76**	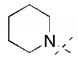	1	38 ^f^ (8, 181)	48 ± 15	>10,000 (9%)
**6**		2	309 (166, 574)	290 ± 7	>10,000 (10%)
**7**		3	252 (64, 990)	(28%)	nt ^e^
**8**		4	225 (98, 519)	(13%)	nt ^e^
**9**	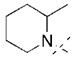	1	69 (49, 96)	11 ± 1	>10,000 (5%)
**10**		2	153 (46, 505)	475 ± 38	>10,000 (2%)
**11**		3	1556 (349, 6941)	(36%)	nt ^e^
**12**	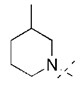	1	98 (43, 226)	117 ±12	>10,000 (10%)
**13**		2	102 (18, 571)	1405 ± 494	nt ^e^
**14**		3	114 (33, 397)	(33%)	nt ^e^
**15**		4	351 (223, 552)	(13%)	nt
**16**	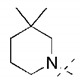	1	1624 (1075, 2453)	476 ± 38	>10,000 (6%)
**17**		2	3437 (2701, 4374)	(38%)	nt ^e^
**18**		3	3535 (2528, 4942)	2777 ± 66	>10,000 (19%)
**19**		4	2575 (542, 12227)	1953 ± 45	>10,000 (24%)
**20**	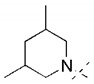	1	341 (49, 2388)	(37%)	nt ^e^
**21**		2	1381 (923, 2066)	(35%)	nt ^e^
**22**		3	2235 (1136, 4397)	(41%)	nt ^e^
**23**		4	2083 (936, 4637)	(34%)	nt ^e^
**24**	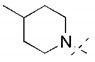	1	316 (123, 808)	(37%)	nt ^e^
**25**		2	400 (152, 1050)	(33%)	nt ^e^
**26**		3	531 (344, 822)	(39%)	nt ^e^
**27**		4	1350 (651, 2798)	(10%)	nt ^e^
**28**	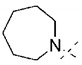	1	111 (68, 180)	45 ± 4	>10,000 (10%)
**29**		2	299 (105, 855)	1627 ± 78	>10,000 (23%)
**30**		3	324 (121, 870)	(18%)	nt ^e^
**31**		4	829 ^g^ (313, 2194)	(23%)	nt ^e^
rasagiline			nt ^e^	15 ± 1	nt ^e^
pargiline			nt ^e^	360 ± 138	nt ^e^
safinamide			nt ^e^	7.7 ± 1.2	nt ^e^
clorgiline			nt ^e^	nt ^e^	1.76 ± 0.5 nM

^a^ [^3^H]*N^α^*-Methylhistamine-binding assay in HEK293 cells stably expressing the human H_3_R; mean value within the 95% confidence interval (CI); ^b^ fluorometricAmplex™ Red MAO assay [15]; mean ± SEM of 2–5 independent experiments; ^c^ the percent of inhibition at 1 μM, mean values of two independent experiments; ^d^ the percent of inhibition at 10 μM, mean values of two independent experiments; ^e^ nt = not tested; ^f^ K_i_ (±SEM) = 22 ± 3 nM in [^125^I]Iodoproxyfan binding assay in CHO K1 cells, data from Reference [13]; ^g^ data from Reference [16].

**Table 2:** V_max_ and K_m_ calculated from Michaelis–Menten curves of compound **9** and **DL76**.

Parameters	Compound 9				DL76			
Concentration (nM)	0	0.2	10	40	0	7	48	164
V_max_ (RFU/min)	1713	1652	1544	1238	2046	1628	1165	726.3
K_M_ (mM)	0.24	0.27	0.27	0.28	0.11	0.14	0.21	0.27

**Table 3:** Antiparkinsonian activity of **DL76** in the bar test.

Compound	Dose	Times of Observation of Catalepsy (s) ^1^		
	(mg/kg; i.p.)	0 min	3 min	6 min
**control**	-	17.5 ± 5.3	30.0 ± 0	30.0 ± 0
**DL-76**	25	19.5± 4.2	26.5 ± 3.5	30.0 ± 0
	50	13.8 ± 5.9	13.5 ± 4.2 *	22.0 ± 5.0
**MSX-3**	25	0 ± 0 ***	0 ± 0 ***	0 ± 0 ***
	50	0 ± 0 ***	0 ± 0 ***	0 ± 0 ***

^1^ mean ± SEM (*n* = 6); * *p* < 0.05; *** *p* < 0.001.

**Table 4:** Antiparkisonian activity of **DL76** in the crossed-leg position test.

Compound	Dose (mg/kg)	Times of Observation of Catalepsy (s) ^1^		
		0 min	3 min	6 min
**control**	-	4.2 ± 1.6	23.5 ± 6.5	30.0 ± 0
**DL-76**	25	0.66 ± 0.68	10.6 ± 6.1	15.0 ± 6.7
	50	0 ± 0 ***	0 ± 0 ***	0.16 ± 0.4 ***
**MSX-3**	25	0 ± 0 ***	0 ± 0 ***	0 ± 0 ***
	50	0 ± 0 ***	0 ± 0 ***	0 ± 0 ***

^1^ mean ± SEM (*n* = 6); *** *p* < 0.001.

## Supplementary Materials

Supplementary Materials can be found at https://www.mdpi.com/1422-0067/21/10/3411/s1.

## Abbreviations

AChE	Acetylcholinesterase
BuChE	Butyrylcholinesterase
DA	Dopamine
DMSO	Dimethyl sulfoxide
DTL	Dual Target Ligands
DX	Doxorubicin
H_3_R	Histamine H_3_ receptor
HEK293	Human embryonic kidney
i.p.	Intraperitoneal
MAO B	Monoamine oxidase B
MTDL	Multi-Target-Directed Ligands
PD	Parkinson’s disease
PEA	β-phenylethylamine
SAL	Salsolinol
s.c.	Subcutaneous
TLC	Thin-layer Chromatography

## References

[1] Draoui A., El Hiba O., Aimrane A., El Khiat A., Gamrani H. (2020). Parkinson’s disease: From bench to bedside. Rev. Neurol..

[2] Szökő É., Tábi T., Riederer P., Vécsei L., Magyar K. (2018). Pharmacological aspects of the neuroprotective effects of irreversible MAO-B inhibitors, selegiline and rasagiline, in Parkinson’s disease. J. Neural. Transm..

[3] Proschak E.J., Stark H., Merk D. (2019). Polypharmacology by Design: A Medicinal Chemist’s Perspective on Multitargeting Compounds. J. Med. Chem..

[4] Panula P., Chazot P.L., Cowart M., Gutzmer R., Leurs R., Liu W.L., Stark H., Thurmond R.L., Haas H.L. (2015). International Union of Basic and Clinical Pharmacology. XCVIII. Histamine Receptors. Pharmacol. Rev..

[5] Sadek B., Łażewska D., Hagenow S., Kieć-Kononowicz K., Stark H., Blandina P., Passani M.B. (2016). Histamine H_3_R antagonists: From scaffold hopping to clinical candidates. Histamine Receptors.

[6] Lamb Y.N. (2020). Pitolisant: A Review in Narcolepsy with or without Cataplexy. CNS Drugs..

[7] Zhou J., Jiang X., He S., Jiang H., Feng F., Liu W., Qu W., Sun H. (2019). Rational Design of Multitarget-Directed Ligands: Strategies and Emerging Paradigms. J. Med. Chem..

[8] Zindo F.T., Joubert J., Malan S.F. (2015). Propargylamine as functional moiety in the design of multifunctional drugs for neurodegenerative disorders: MAO inhibition and beyond. Future Med. Chem..

[9] Bautista-Aguilera Ó.M., Hagenow S., Palomino-antolin A., Farré-Alins V., Ismaili L., Joffrin P.L., Jimeno M.L., Soukup O., Janočková J., Kalinowsky L. (2018). Multitarget-directed ligands combining cholinesterase and monoamine oxidase inhibition with histamine H_3_R antagonism for neurodegenerative diseases. Angew. Chem. Int. Ed. Engl..

[10] Lutsenko K., Hagenow S., Affini A., Reiner D., Stark H. (2019). Rasagiline derivatives combined with histamine H_3_ receptor properties. Bioorg. Med. Chem. Lett..

[11] Affini A., Hagenow S., Zivkovic A., Marco-Contelles J., Stark H. (2018). Novel indanone derivatives as MAO B/H_3_R dual-targeting ligands for treatment of Parkinson’s disease. Eur. J. Med. Chem..

[12] Łażewska D., Olejarz-Maciej A., Kaleta M., Bajda M., Siwek A., Karcz T., Doroz-Płonka A., Cichoń U., Kuder K., Kieć-Kononowicz K. (2018). 4-tert-Pentylphenoxyalkyl derivatives—Histamine H_3_ receptor ligands and monoamine oxidase B inhibitors. Bioorg. Med. Chem. Lett..

[13] Łażewska D., Ligneau X., Schwartz J.C., Schunack W., Stark H., Kieć-Kononowicz K. (2006). Ether derivatives of 3-piperidinopropan-1-ol as non-imidazole histamine H_3_ receptor antagonists. Bioorg. Med. Chem..

[14] Kuder K.J., Łażewska D., Latacz G., Schwed J.S., Karcz T., Stark H., Karolak-Wojciechowska J., Kieć-Kononowicz K. (2016). Chlorophenoxyaminoalkyl derivatives as histamine H_3_R ligands and antiseizure agents. Bioorg. Med. Chem..

[15] Zhou M., Panchuk-Voloshina N. (1997). A one-step fluorometric method for the continuous measurement of monoamine oxidase activity. Anal. Biochem..

[16] Podlewska S., Latacz G., Łażewska D., Kieć-Kononowicz K., Handzlik J. (2020). In silico and in vitro studies on interaction of novel non-imidazole histamine H_3_R ligands with CYP3A4. Bioorg. Med. Chem. Lett..

[17] Copeland R.A. (2005). Evaluation of Enzyme Inhibitors in Drug Discovery. A Guide for Medicinal Chemists and Pharmacologists.

[18] Ramsay R.R., Tipton K.F. (2017). Assessment of Enzyme Inhibition: A Review with Examples from the Development of Monoamine Oxidase and Cholinesterase Inhibitory Drugs. Molecules.

[19] Copeland R.A. (2000). Enzymes: A Practical Introduction to Structure, Mechanism, and Data Analysis.

[20] Kurnik-Łucka M., Latacz G., Martyniak A., Bugajski A., Kieć-Kononowicz K., Gil K. (2020). Salsolinol-neurotoxic or neuroprotective?. Neurotox. Res..

[21] Duty S., Jenner P. (2011). Animal models of Parkinson’s disease: A source of novel treatments and clues to the cause of the disease. Br. J. Pharmacol..

[22] Hauber W., Neuscheler P., Nagel J., Müller C.E. (2001). Catalepsy induced by a blockade of dopamine D1 or D2 receptors was reversed by a concomitant blockade of adenosine A(2A) receptors in the caudate-putamen of rats. Eur. J. Neurosci..

[23] Kottke T., Sander K., Weizel L., Schneider E.H., Seifert R., Stark H. (2011). Receptor-specific functional efficacies of alkyl imidazoles as dual histamine H_3_/H_4_ receptor ligands. Eur. J. Pharmacol..

[24] Cheng Y.C., Prusoff W. (1973). Relationship between the inhibition constant (KI) and the concentration of inhibitor which causes 50 per cent inhibition (I50) of an enzymatic reaction. Biochem. Pharmacol..

[25] Załuski M., Schabikowski J., Schlenk M., Olejarz-Maciej A., Kubas B., Karcz T., Kuder K., Latacz G., Zygmunt M., Synak D. (2019). Novel multi-target directed ligands based on annelated xanthine scaffold with aromatic substituents acting on adenosine receptor and monoamine oxidase B. Synthesis, in vitro and in silico studies. Bioorg. Med. Chem..

[26] Tzvetkov N.T., Hinz S., Küppers P., Gastreich M., Müller C.E. (2014). Indazole-and Indole-5-Carboxamides: Selective and Reversible Monoamine Oxidase B Inhibitors with Subnanomolar Potency. J. Med. Chem..

[27] Latacz G., Kechagioglou P., Papi R., Łażewska D., Więcek M., Kamińska K., Wencel P., Karcz T., Schwed J.S., Stark H. (2016). The Synthesis of 1,3,5-triazine Derivatives and JNJ7777120 Analogues with Histamine H_4_ Receptor Affinity and Their Interaction with PTEN Promoter. Chem. Biol. Drug Des..

[28] Prinssen E.P.M., Kleven M.S., Koek W. (1999). Interactions between neuroleptics and 5-HT1A ligands in preclinical behavioral models for antipsychotic and extrapyramidal effects. Psychopharmacology.

