# Supplementary material for: Dual Target Ligands with 4-tert-Butylphenoxy Scaffold as Histamine H3 Receptor Antagonists and Monoamine Oxidase B Inhibitors
Source: Int J Mol Sci. 2020 May 12;21(10):3411. doi: 10.3390/ijms21103411 (PMC7279487; doi:10.3390/ijms21103411)
Supplement: Supplementary file 1 [file ijms-21-03411-s001.zip › ijms-792322-Supplementary Materials/Supplementary Materials S1.pdf]

Supplementary Materials S1

Dual target ligands with 4-*tert*-butylphenoxy scaffold as histamine H_3 receptor antagonists and monoamine oxidase B inhibitors

Dorota Łażewska[1*], Agnieszka Olejarz-Maciej[1], David Reiner[2], Maria Kaleta[1], Gniewomir Latacz[1], Małgorzata Zygmunt[3], Agata Doroz-Płonka[1], Tadeusz Karcz[1], Annika Frank[2], Holger Stark[2] and Katarzyna Kieć-Kononowicz[1*]

[1]Department of Technology and Biotechnology of Drugs, Jagiellonian University Medical College, 9 Medyczna str, 30-688 Kraków, Poland; agnieszka.olejarz@uj.edu.pl (A.O.-M.); maria.kaleta@uj.edu.pl (M.K.); glatacz@cm-uj.krakow.pl (G.L.); a.doroz-plonka@uj.edu.pl (A.D.-P.); t.karcz@uj.edu.pl (T.K.); mfkonono@cyf-kr.edu.pl (K.K-K.)

[2]Institute of Pharmaceutical and Medicinal Chemistry, Heinrich Heine University Düsseldorf, Universitaetsstr. 1, 40225 Duesseldorf, Germany; david.reiner@hhu.de (D.R.); a.frank@hhu.de (A.F.); stark@hhu.de (H.S.)

[3]Department of Pharmacodynamics, Jagiellonian University Medical College, 9 Medyczna str, 30-688, Kraków, Poland; malgorzata.zygmunt@uj.edu.pl (M.Z.)

*Correspondence: dlazewska@cm-uj.krakow.pl (D.Ł.), mfkono@cyf-kr.edu.pl (K.K-K.)

^{1}H NMR and ^{13}C NMR data of synthesized compounds

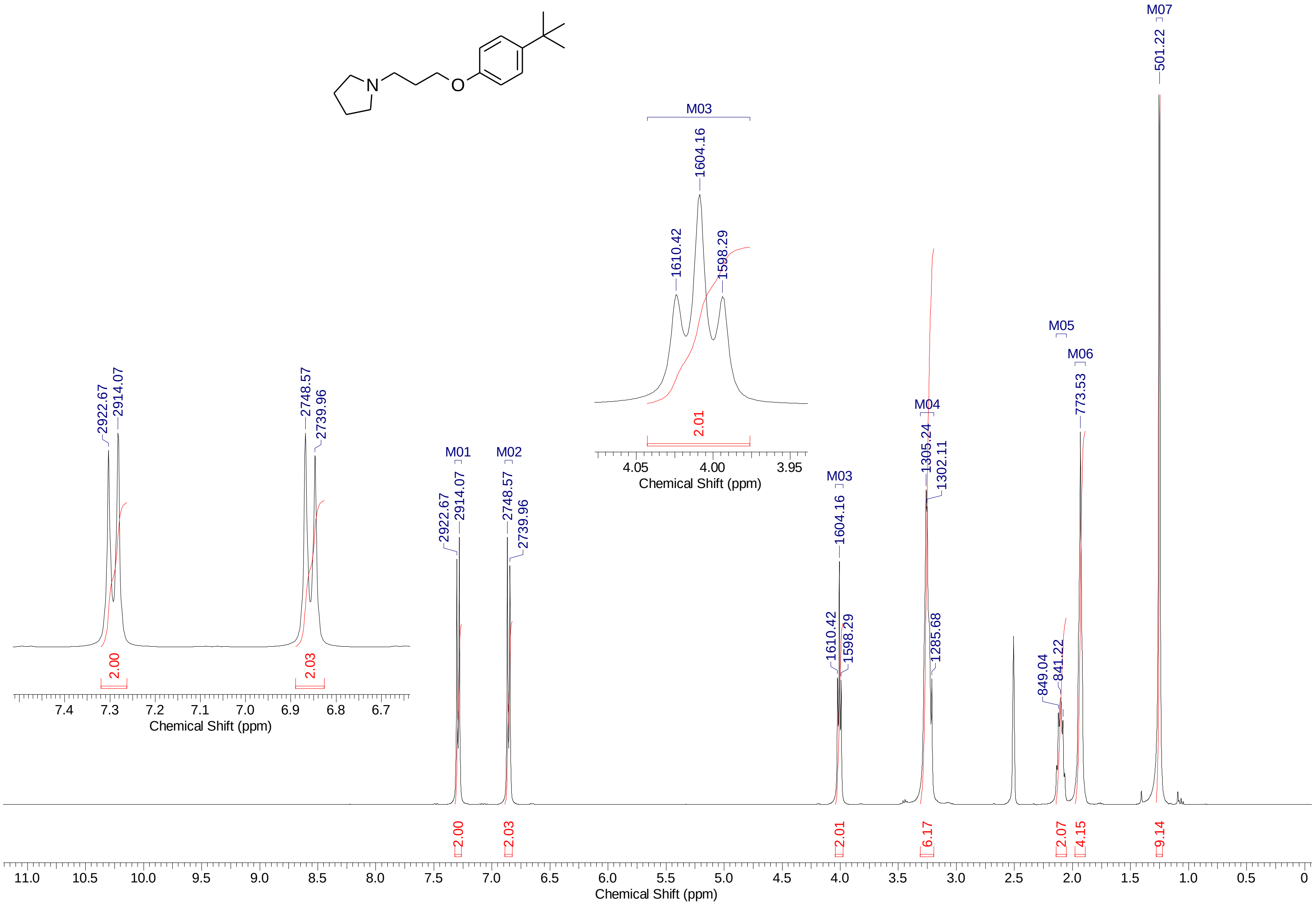

Fig S1. ^{1}H NMR spectrum of **5**

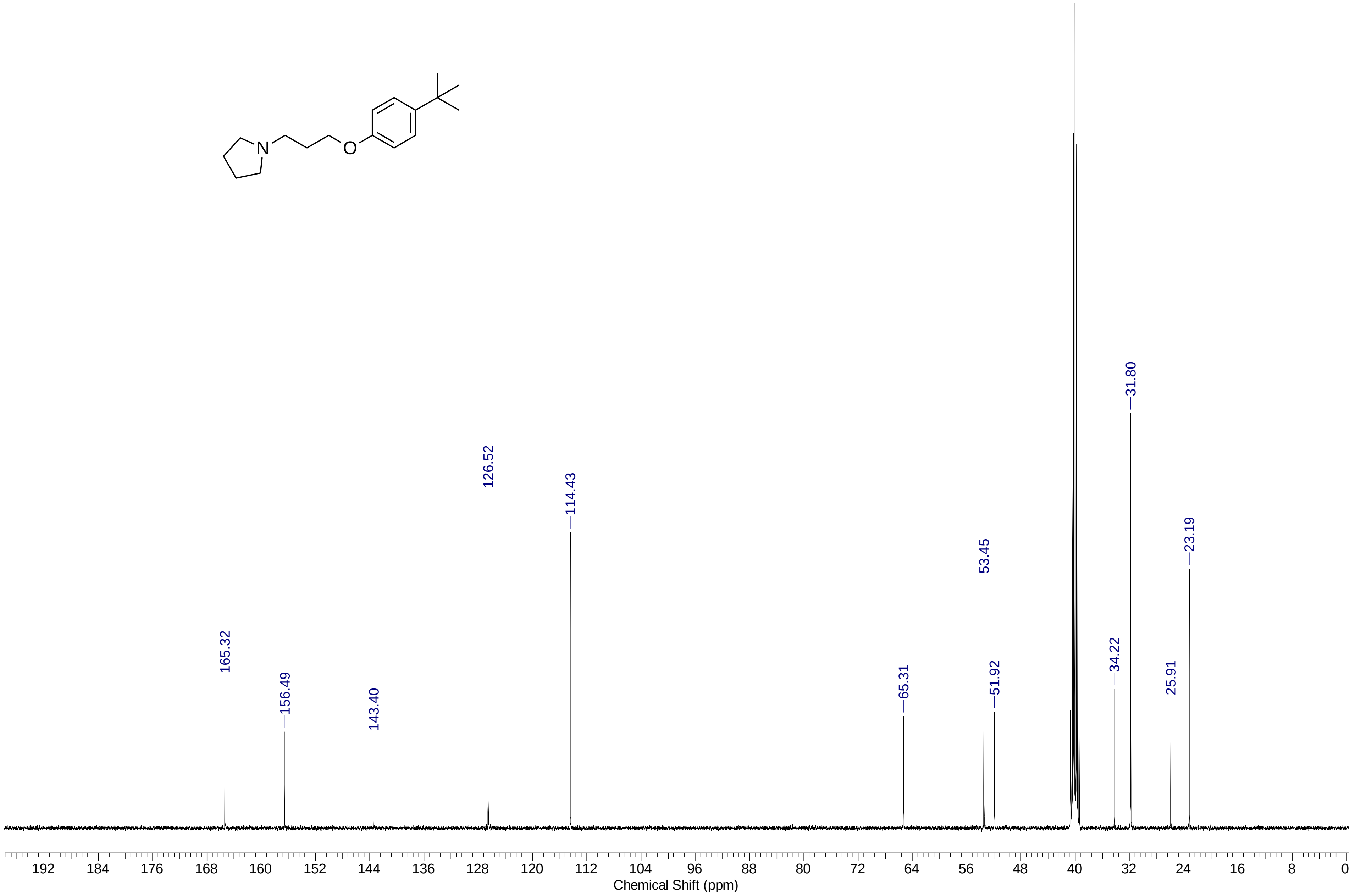

Fig S2. ^{13}C NMR spectrum of **5**

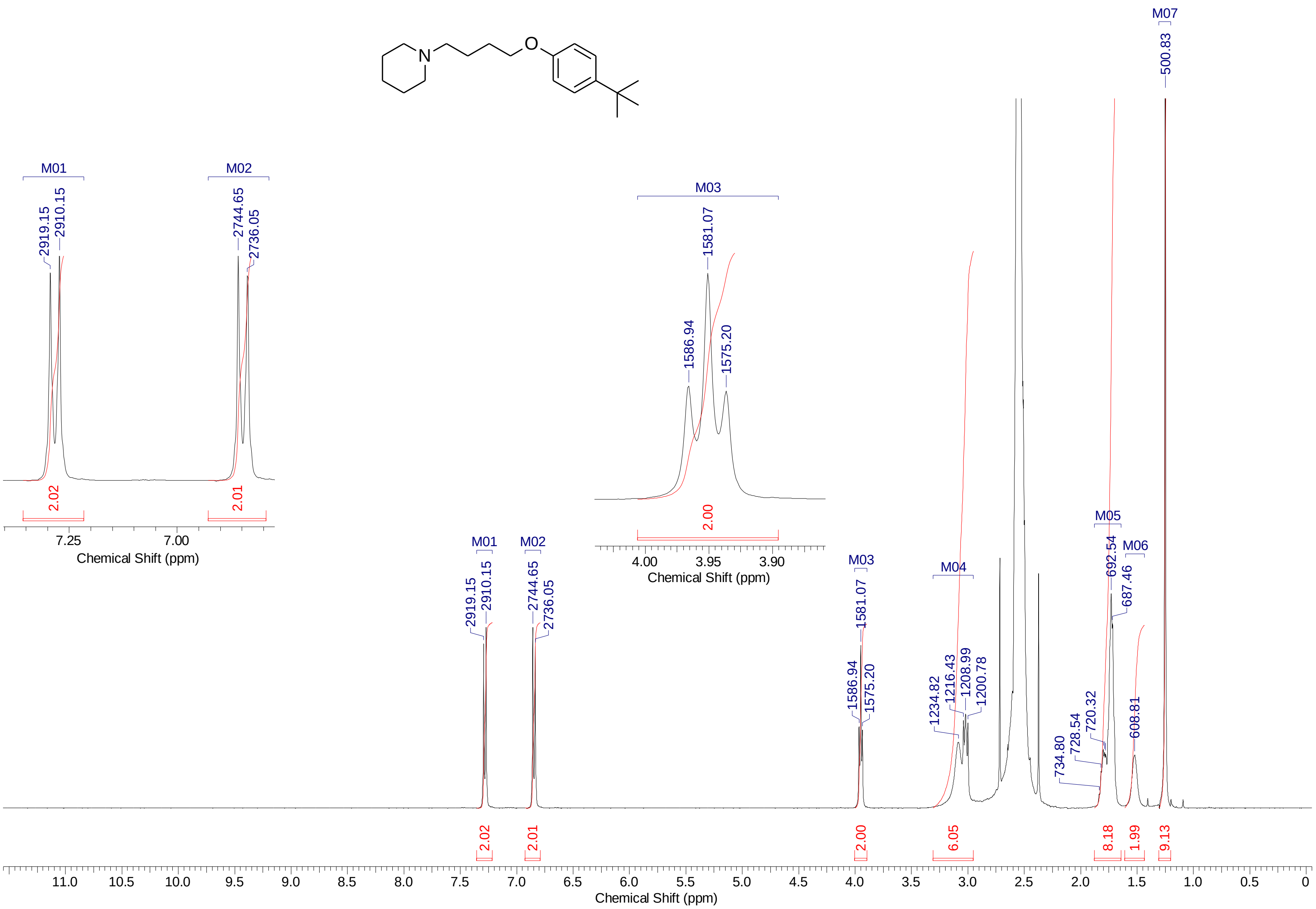

Fig S3. ^{1}H NMR spectrum of **6**

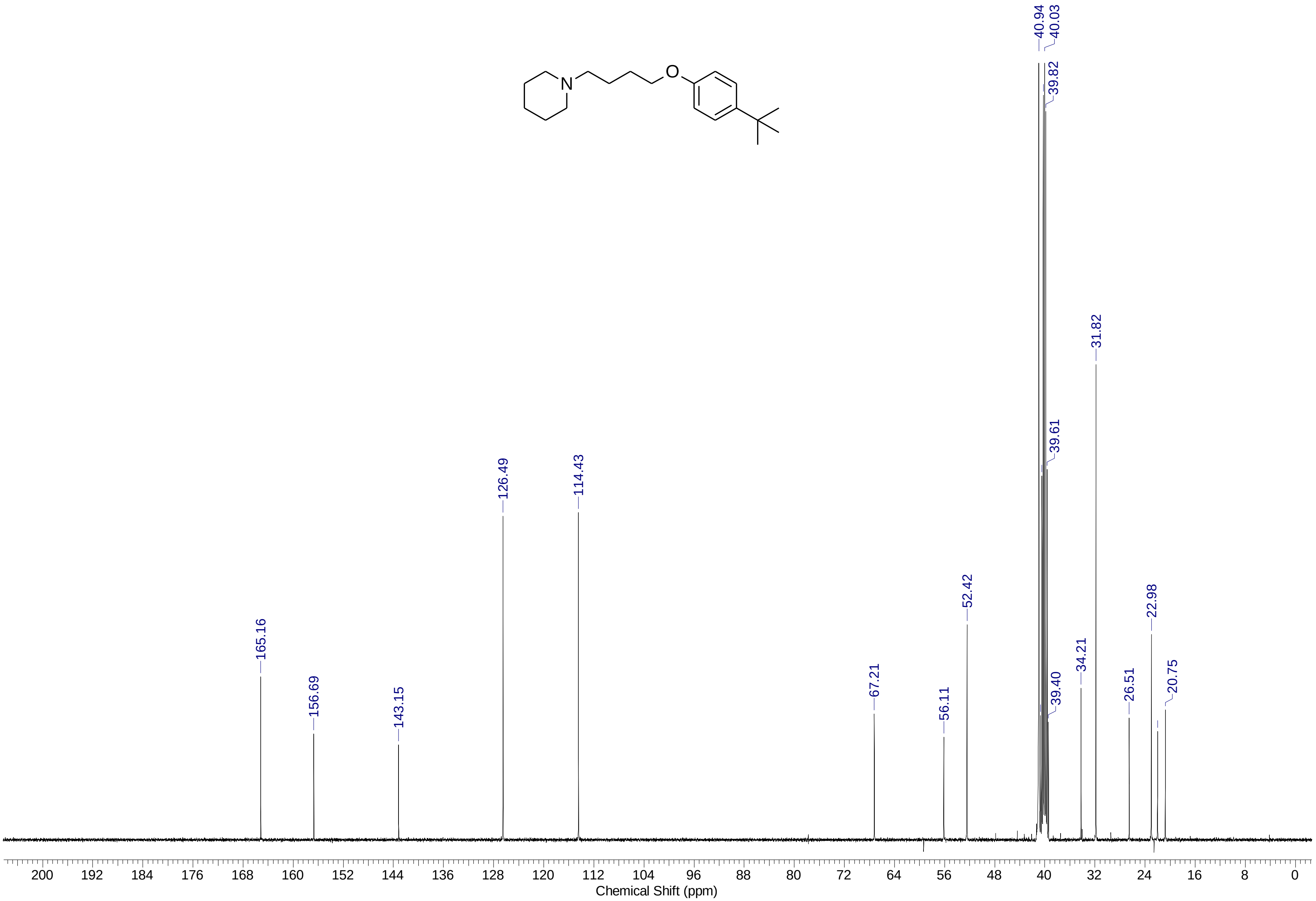

Fig S4. ^{13}C NMR spectrum of **6**

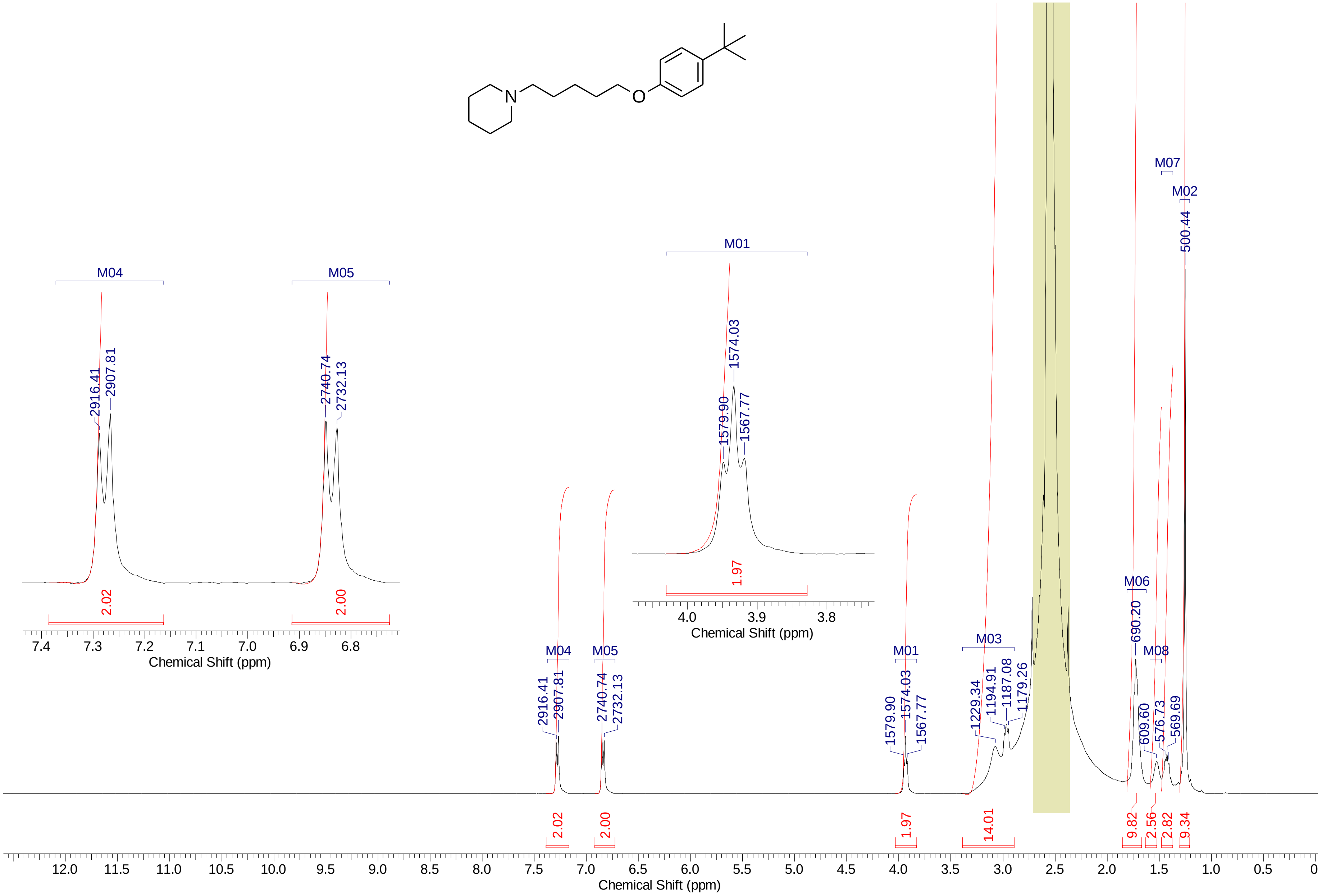

Fig S5. ^{1}H NMR spectrum of **7**

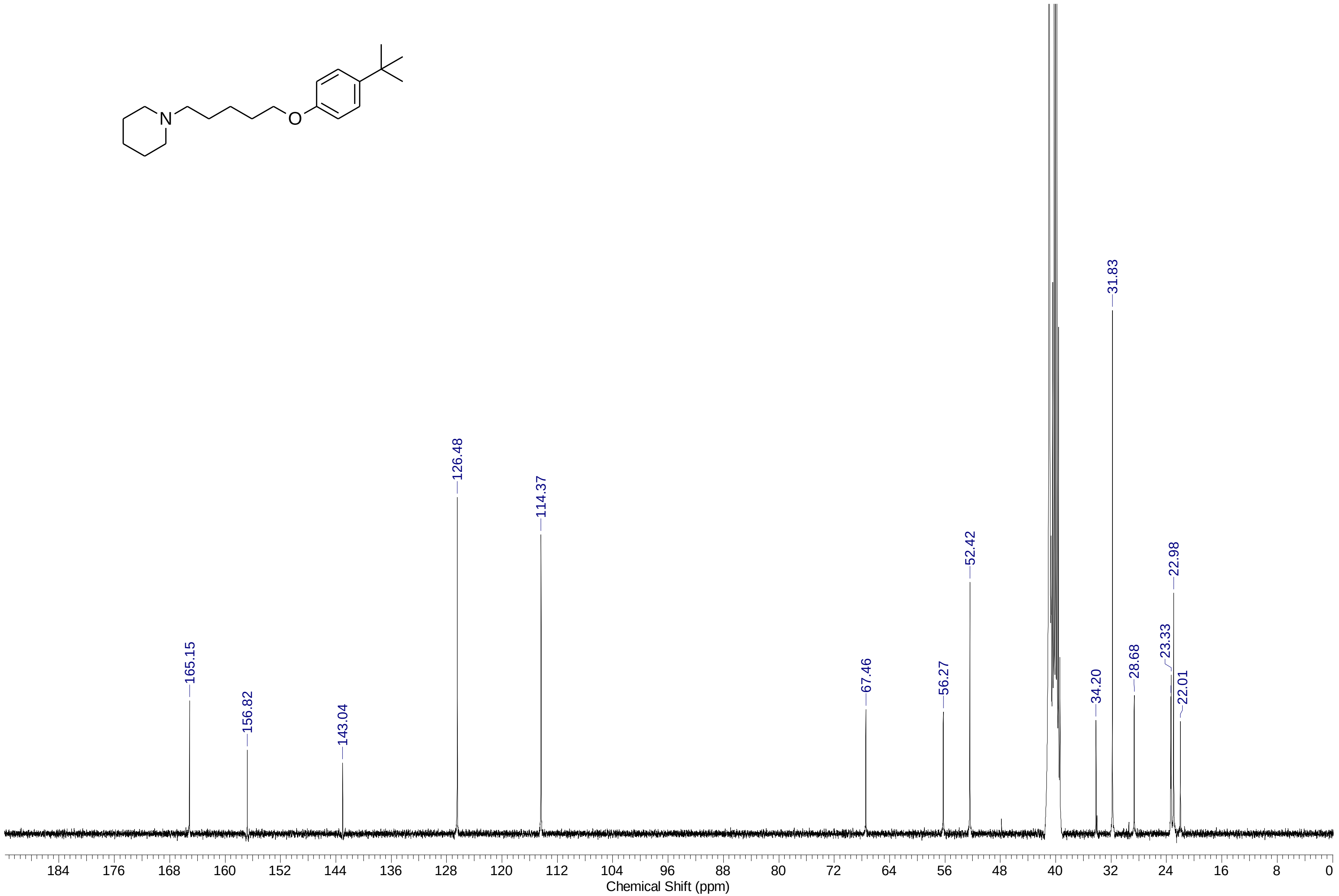

Fig S6. ^{13}C NMR spectrum of **7**

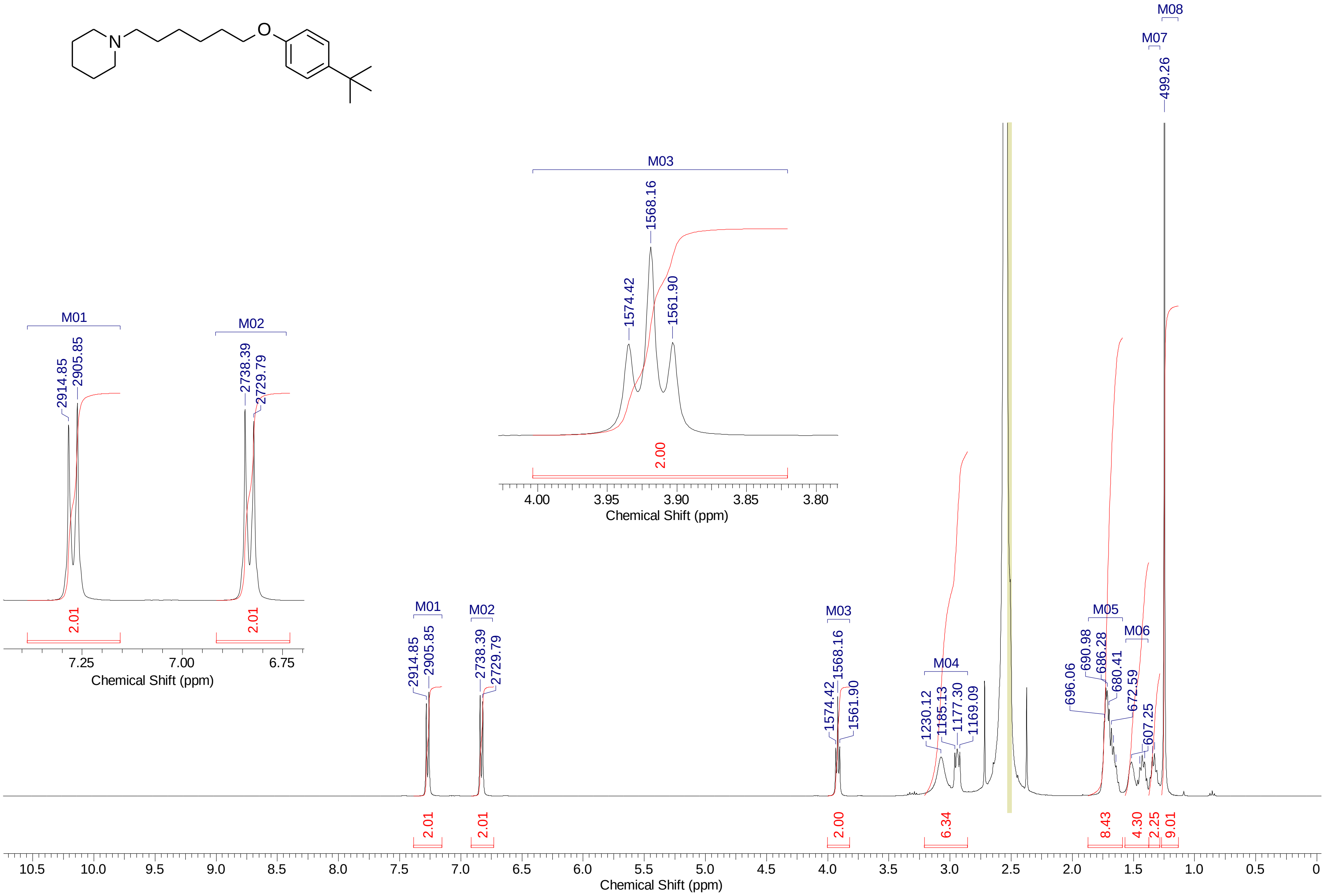

Fig S7. ^{1}H NMR spectrum of **8**

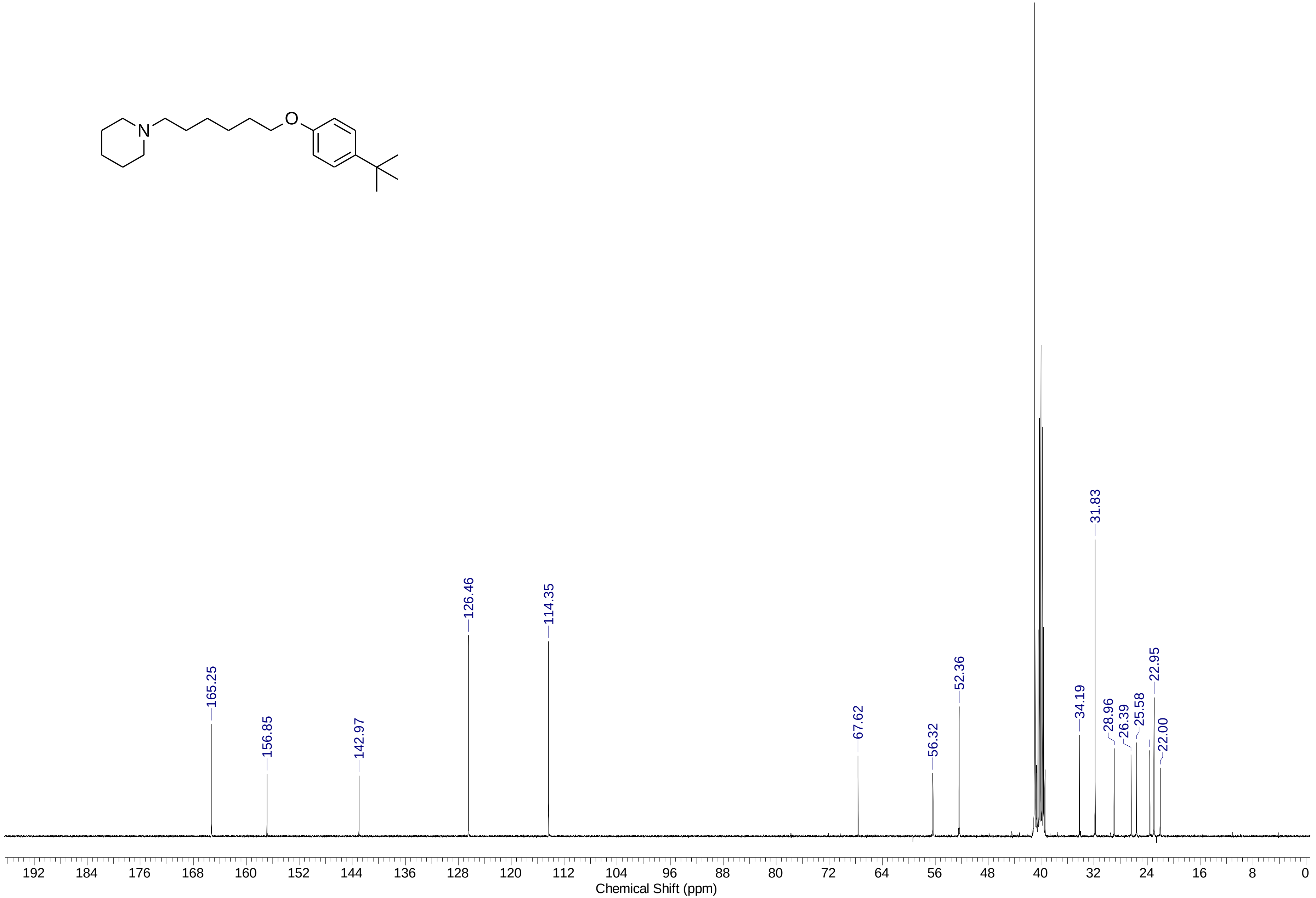

Fig S8. ^{13}C NMR spectrum of **8**

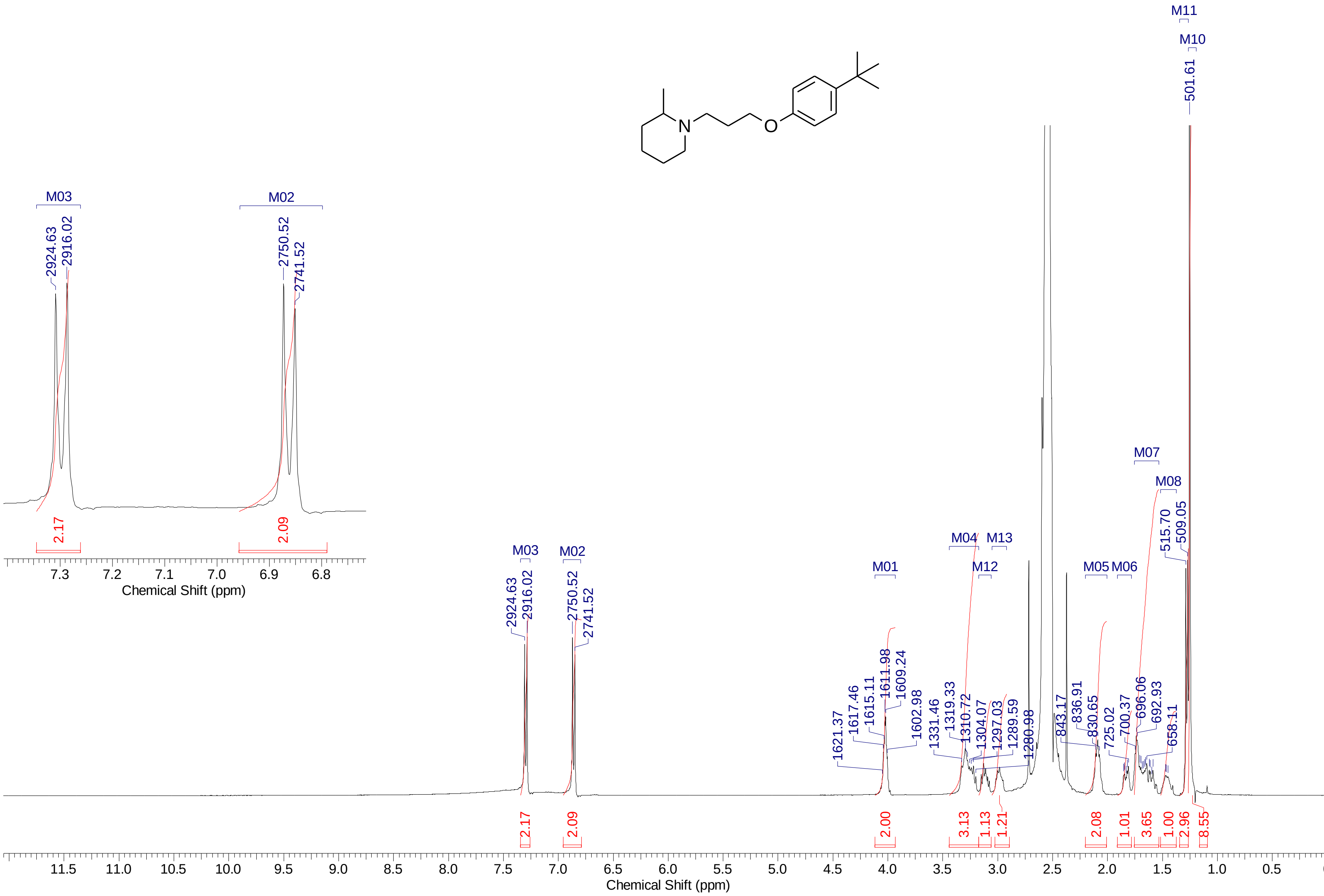

Fig S9. ^{1}H NMR spectrum of **9**

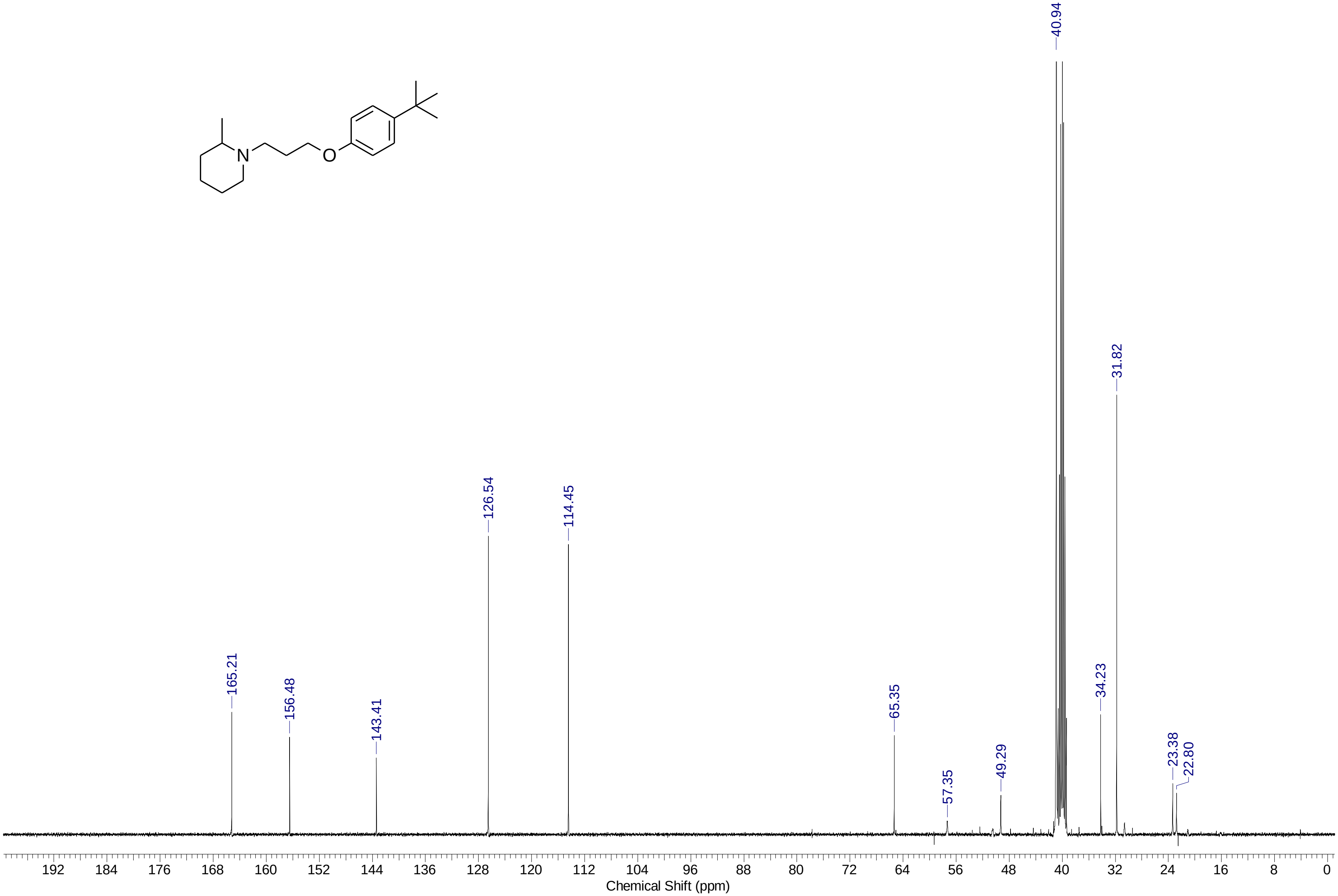

Fig S10. ^{13}C NMR spectrum of **9**

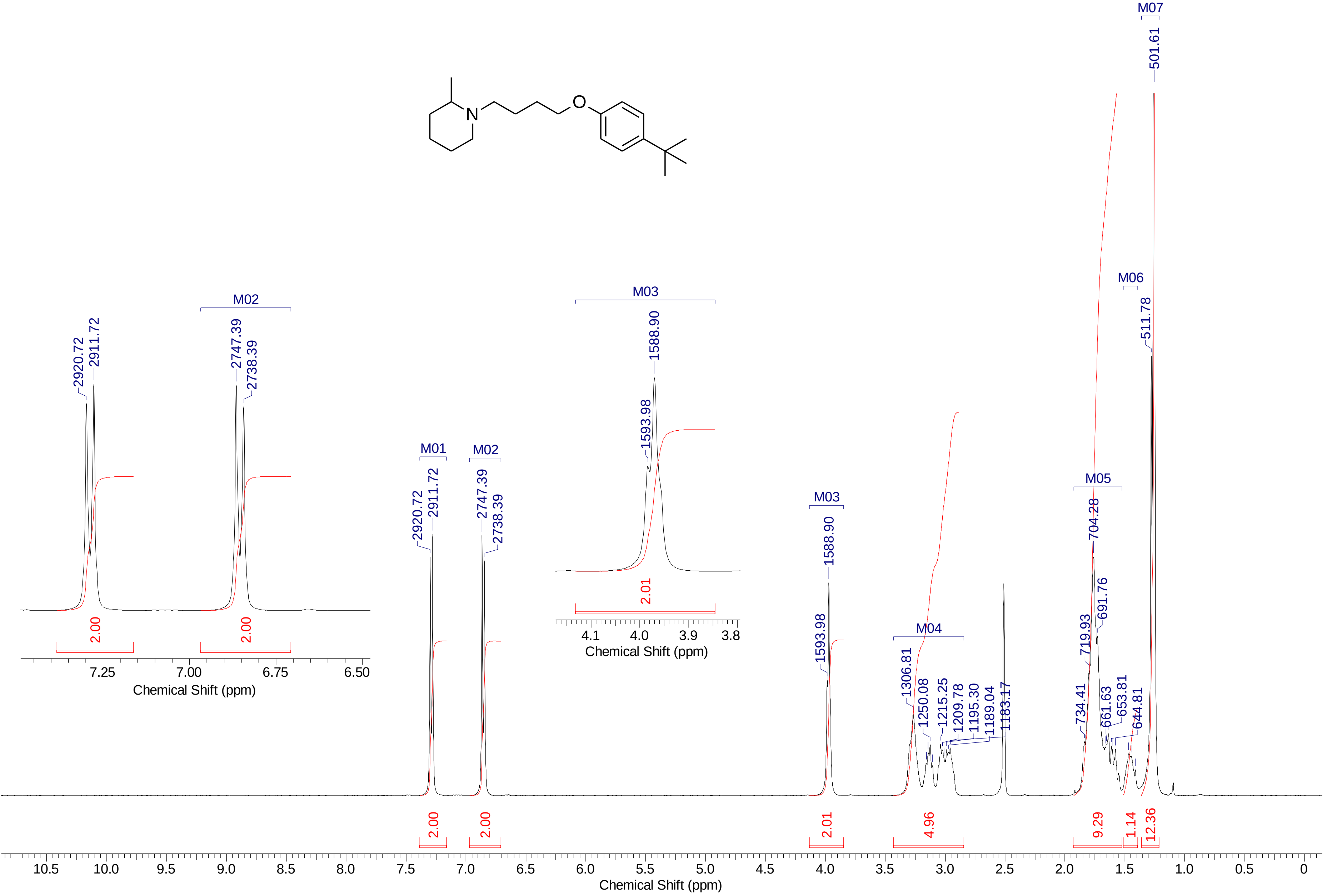

Fig S11. ^{1}H NMR spectrum of **10**

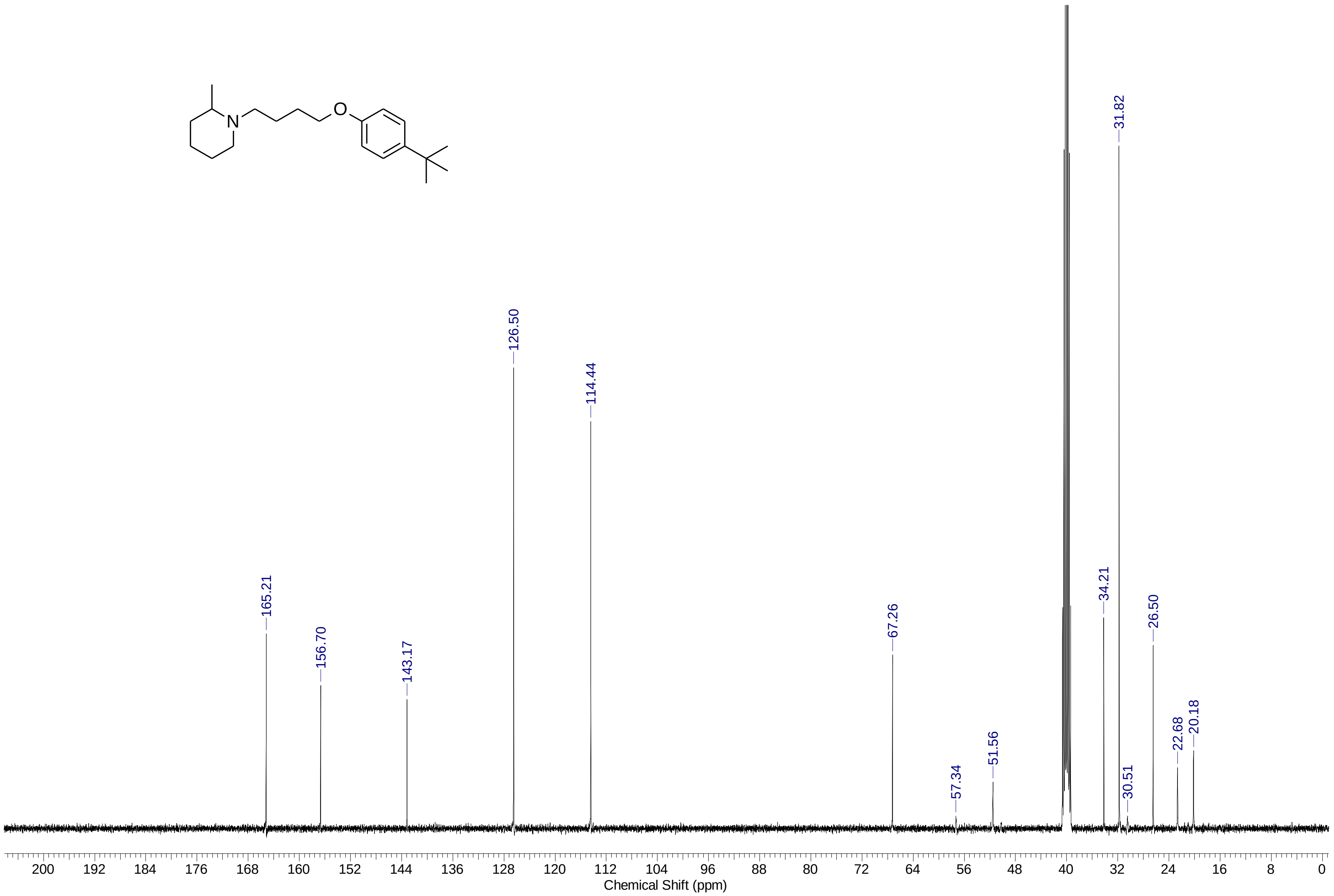

Fig S12. ^{13}C NMR spectrum of **10**

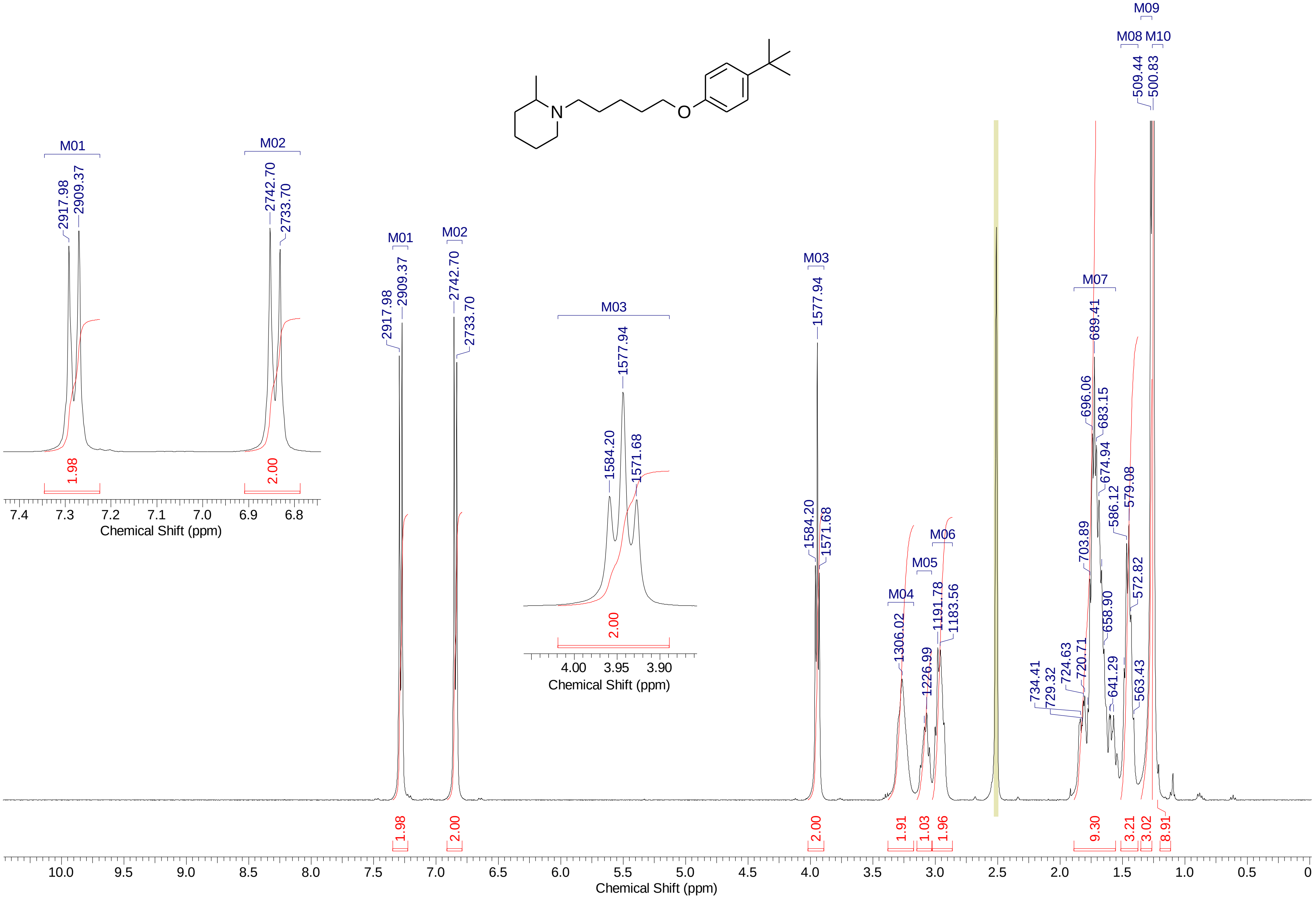

Fig S13. ^{1}H NMR spectrum of **11**

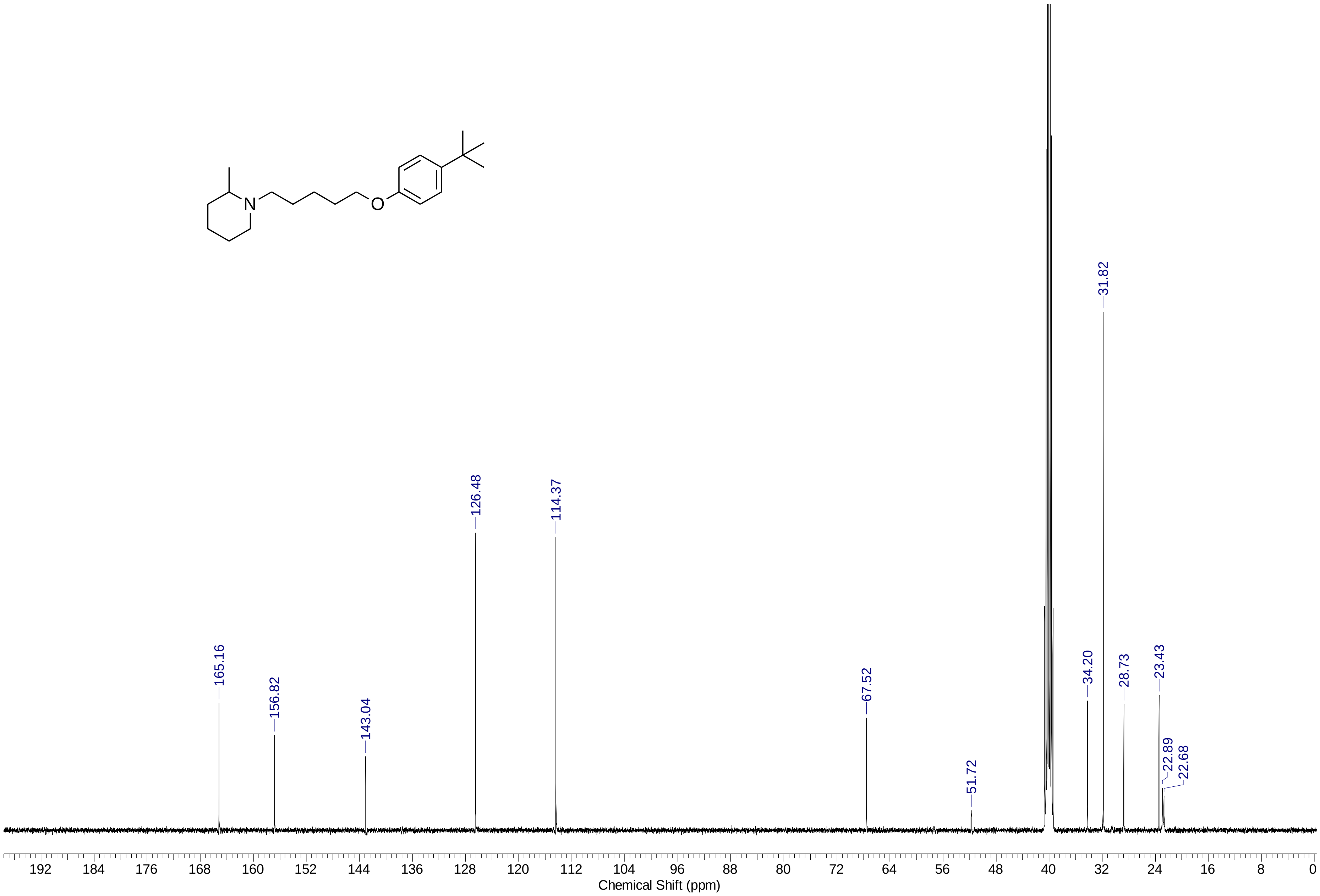

Fig S14. ^{13}C NMR spectrum of **11**

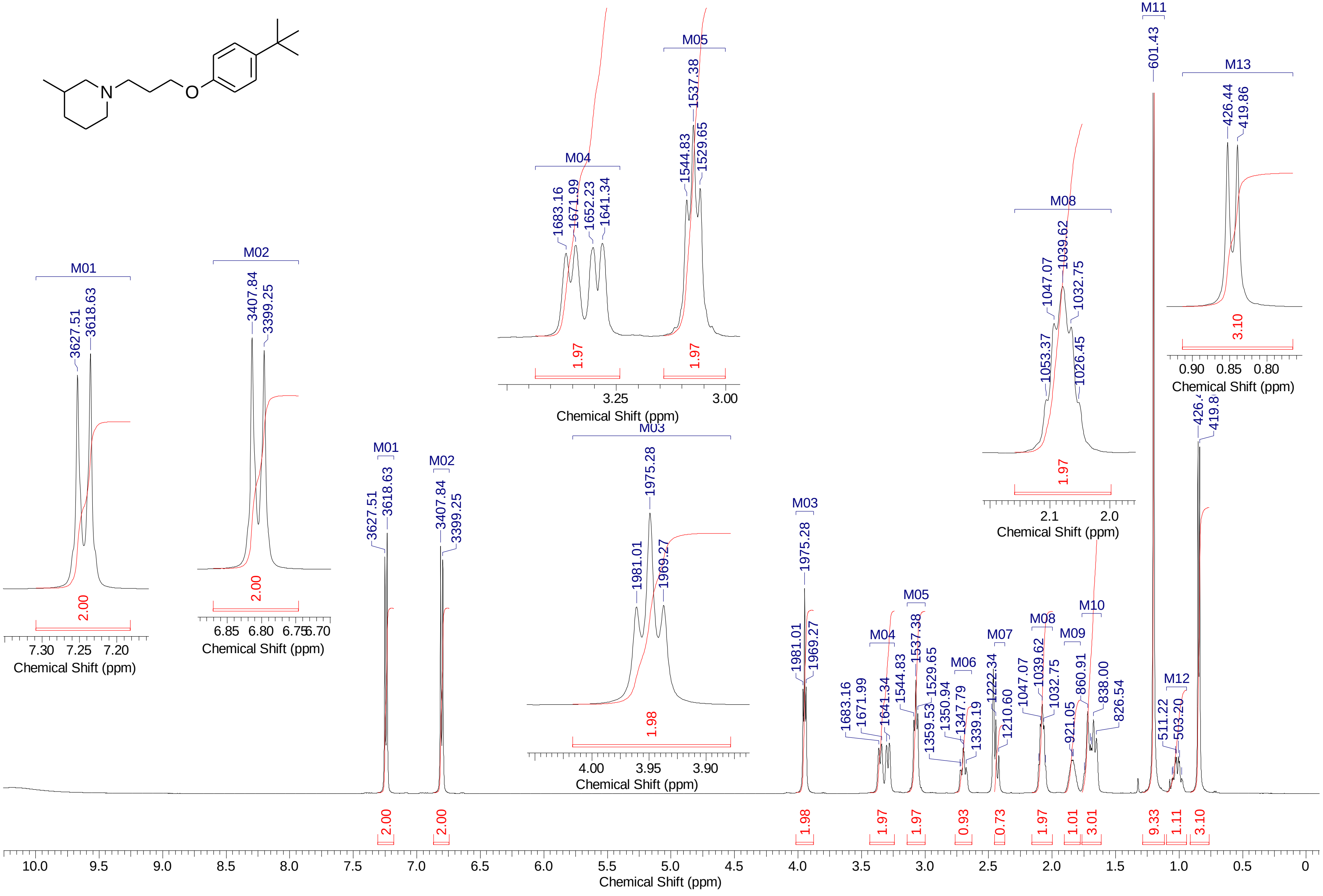

Fig S15. ^{1}H NMR spectrum of **12**

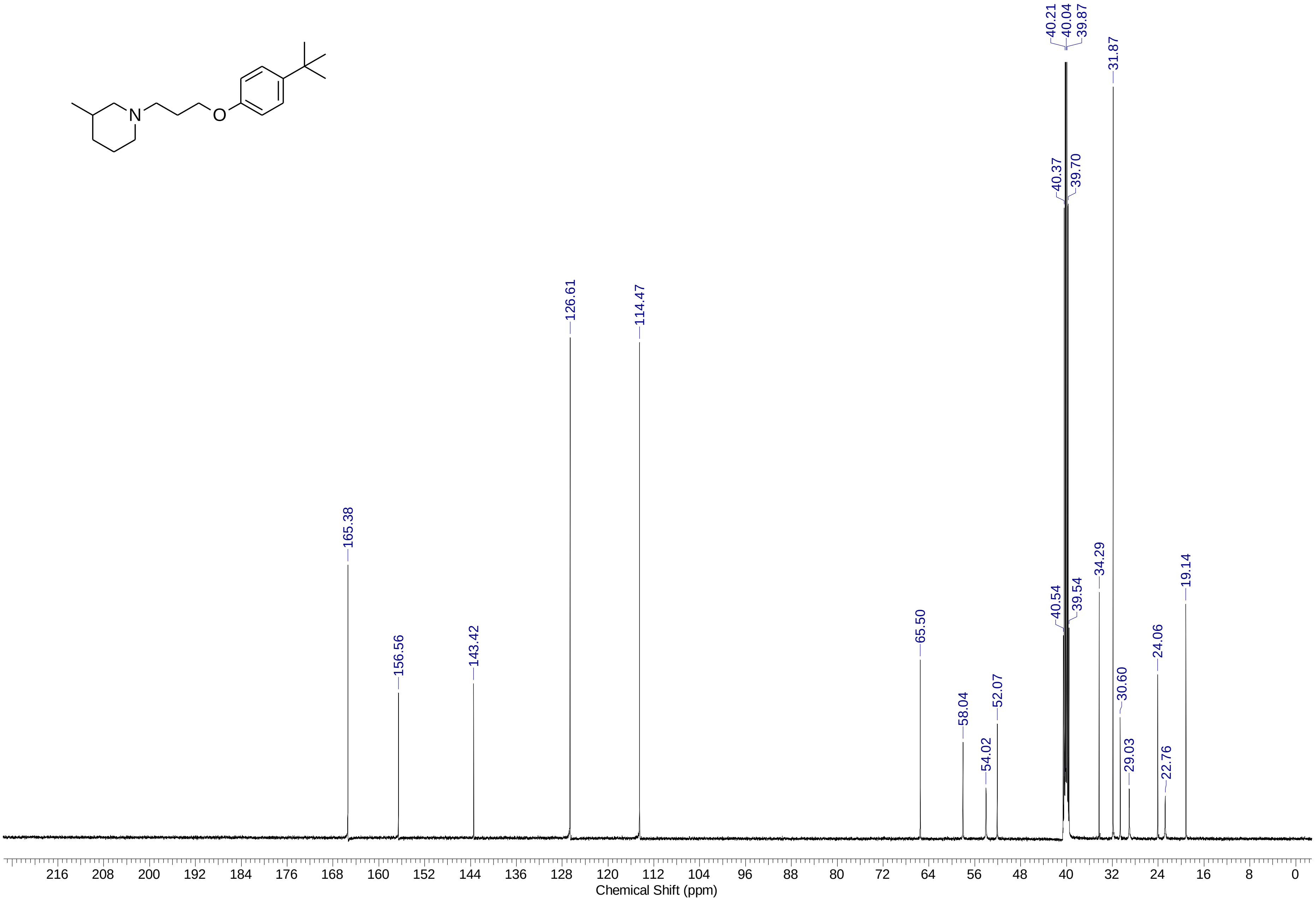

Fig S16. ^{13}C NMR spectrum of **12**

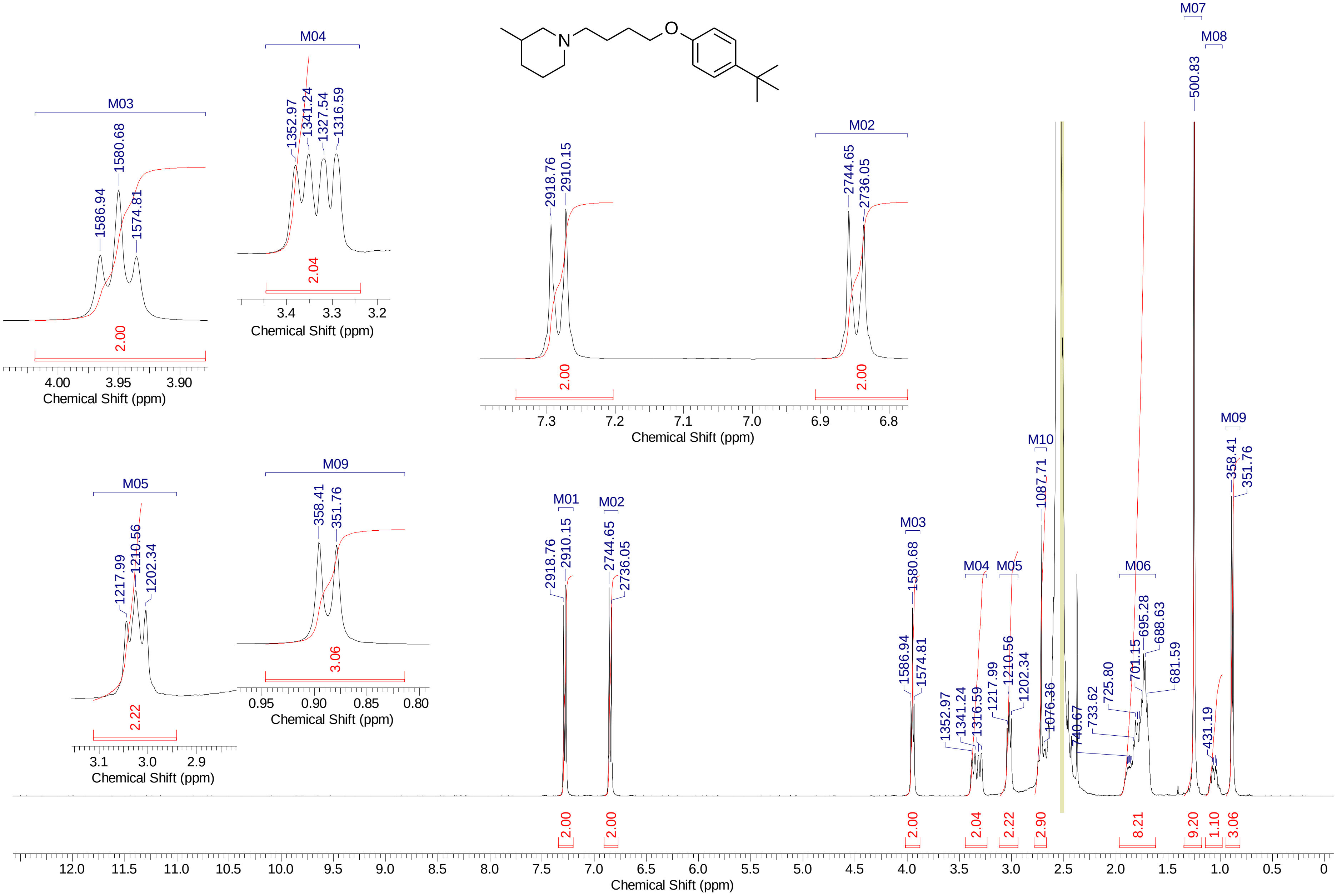

Fig S17. ^{1}H NMR spectrum of **13**

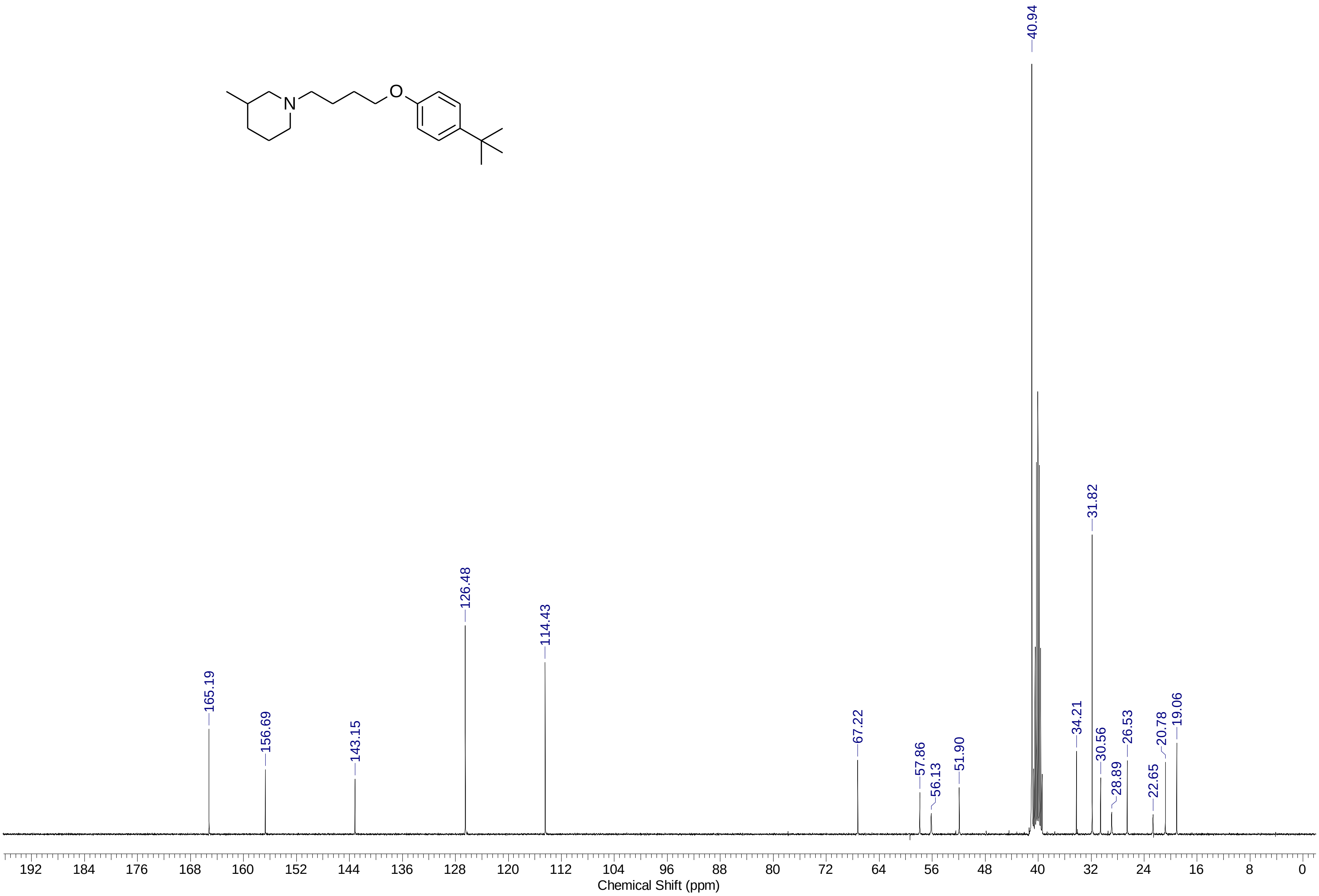

Fig S18. ^{13}C NMR spectrum of **13**

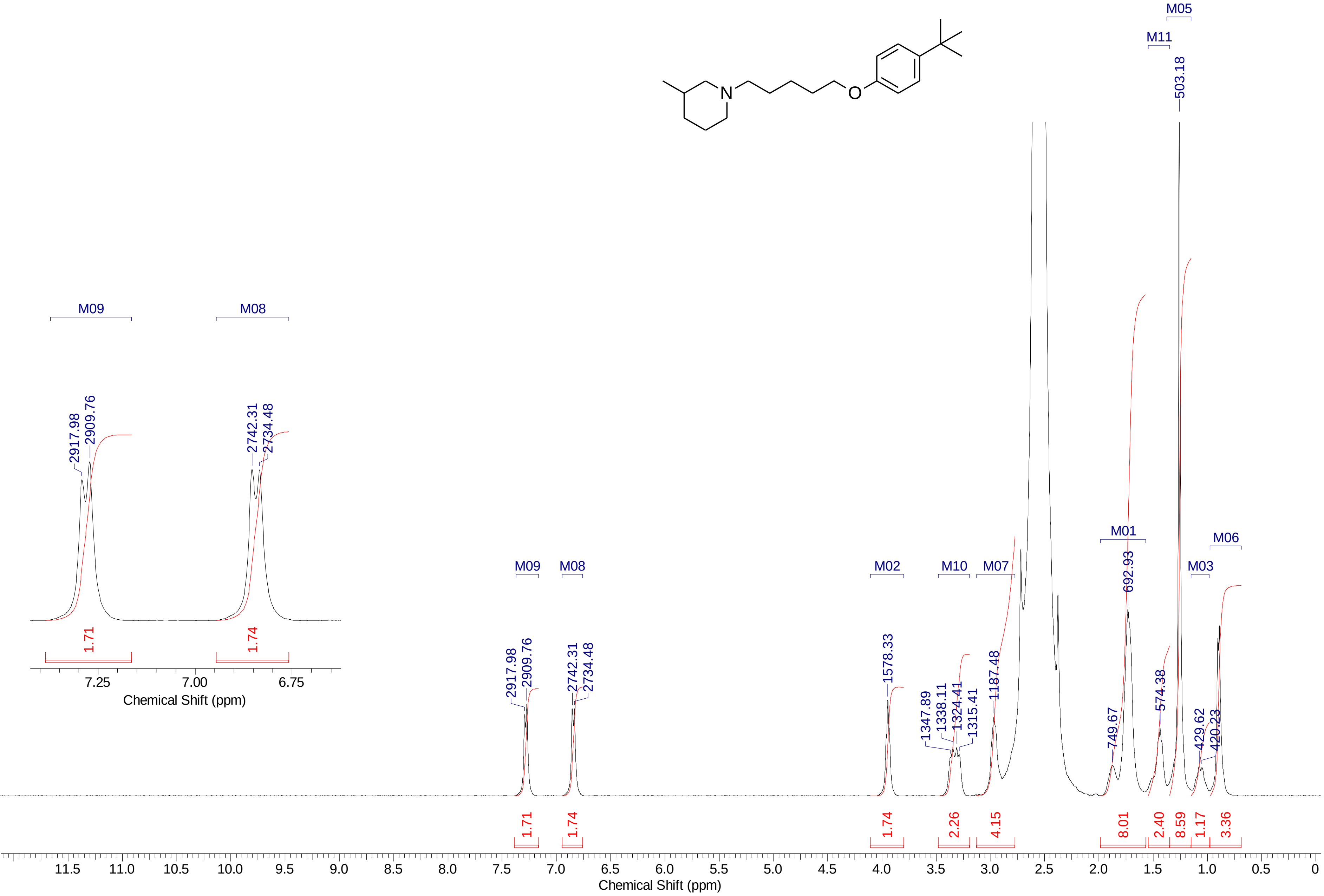

Fig S19. ^{1}H NMR spectrum of **14**

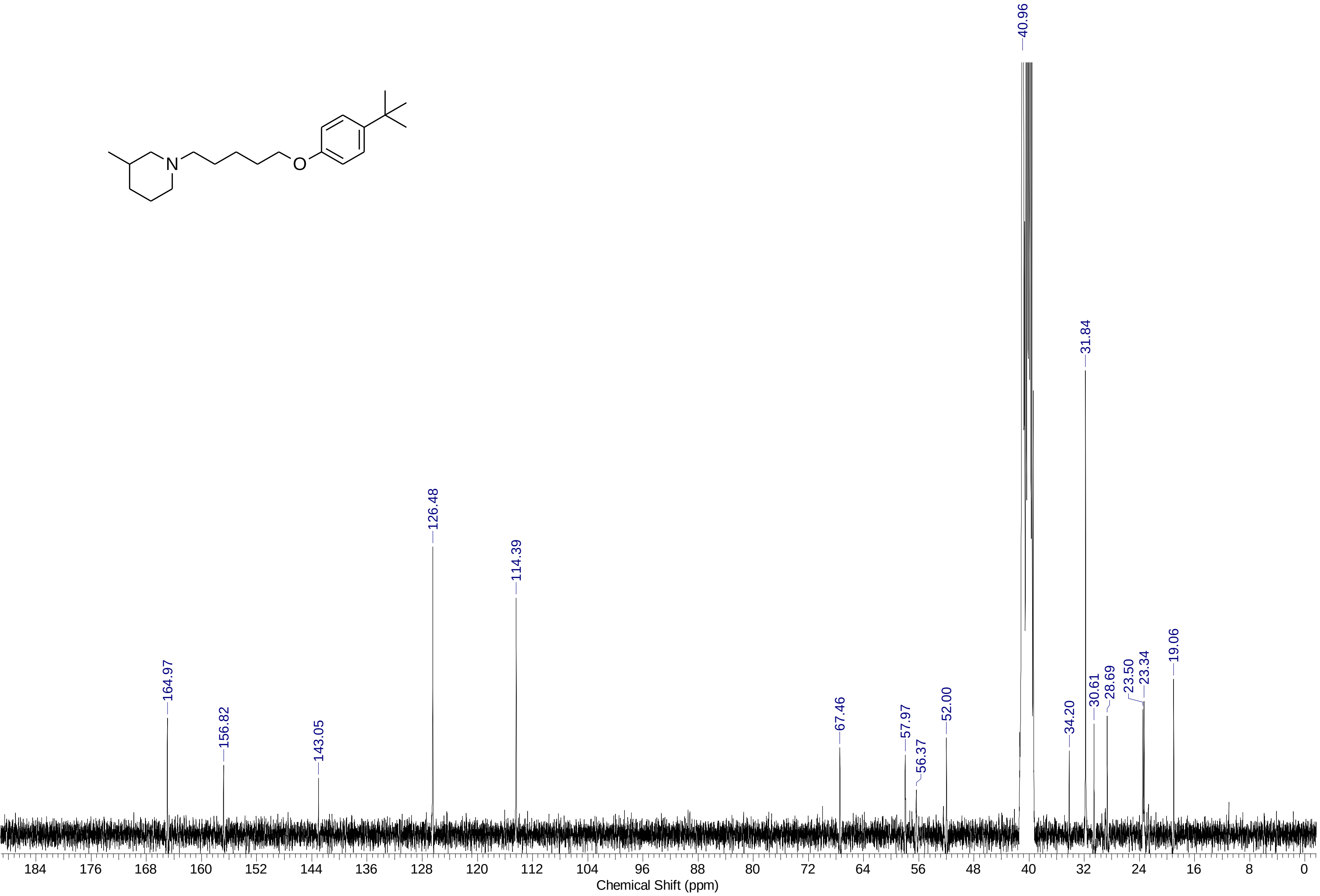

Fig S20. ^{13}C NMR spectrum of **14**

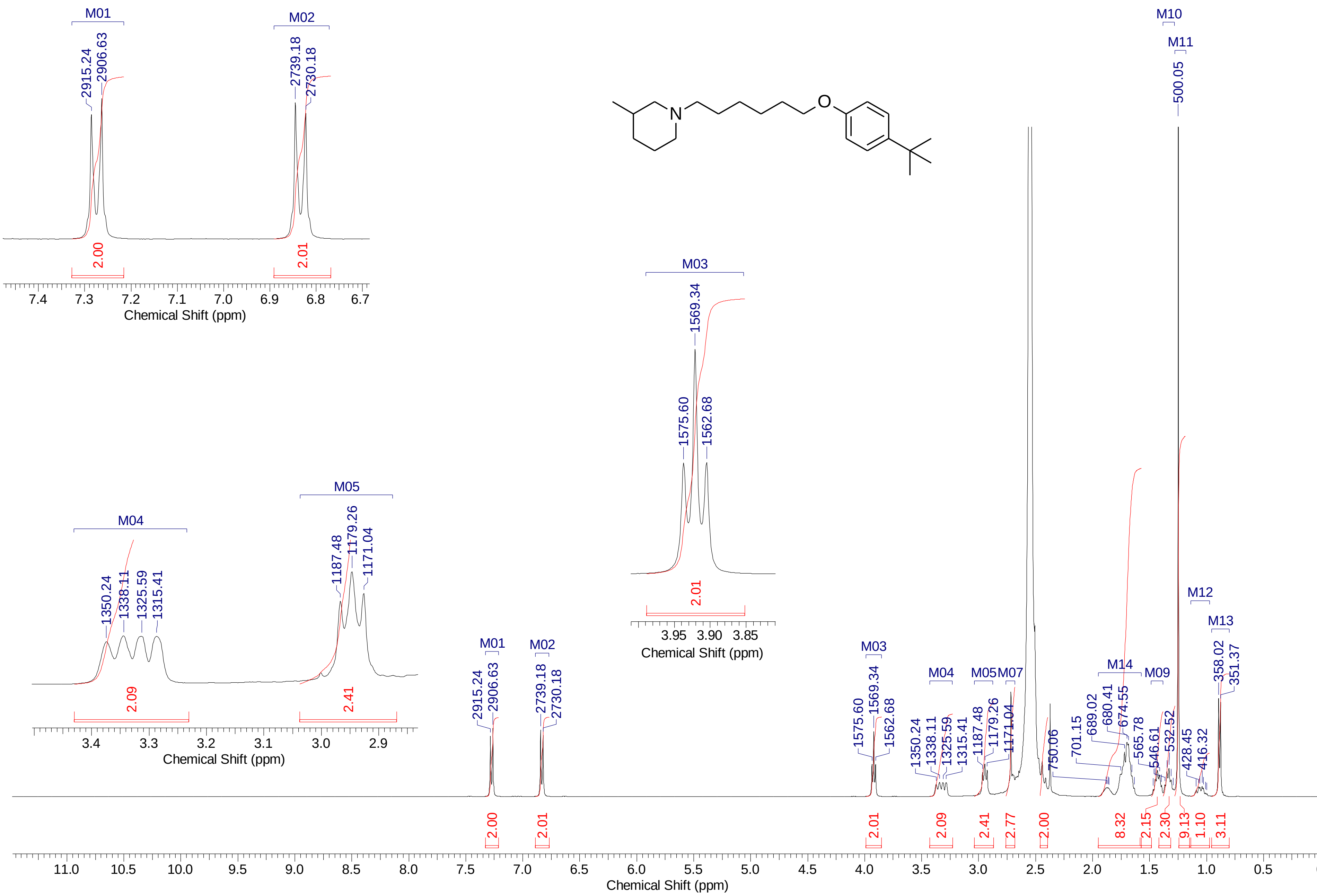

Fig S21. ^{1}H NMR spectrum of **15**

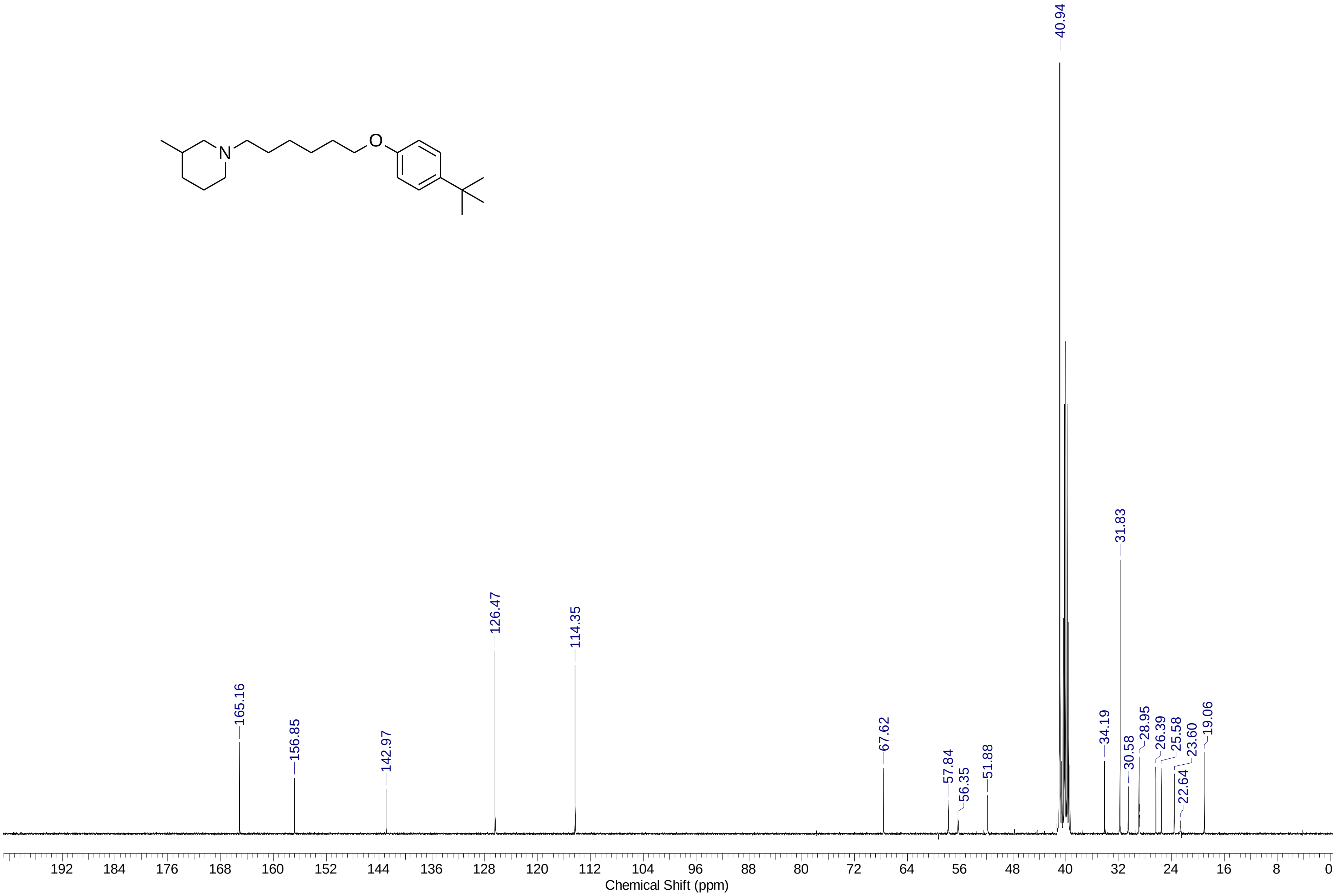

Fig S22. ^{13}C NMR spectrum of **15**

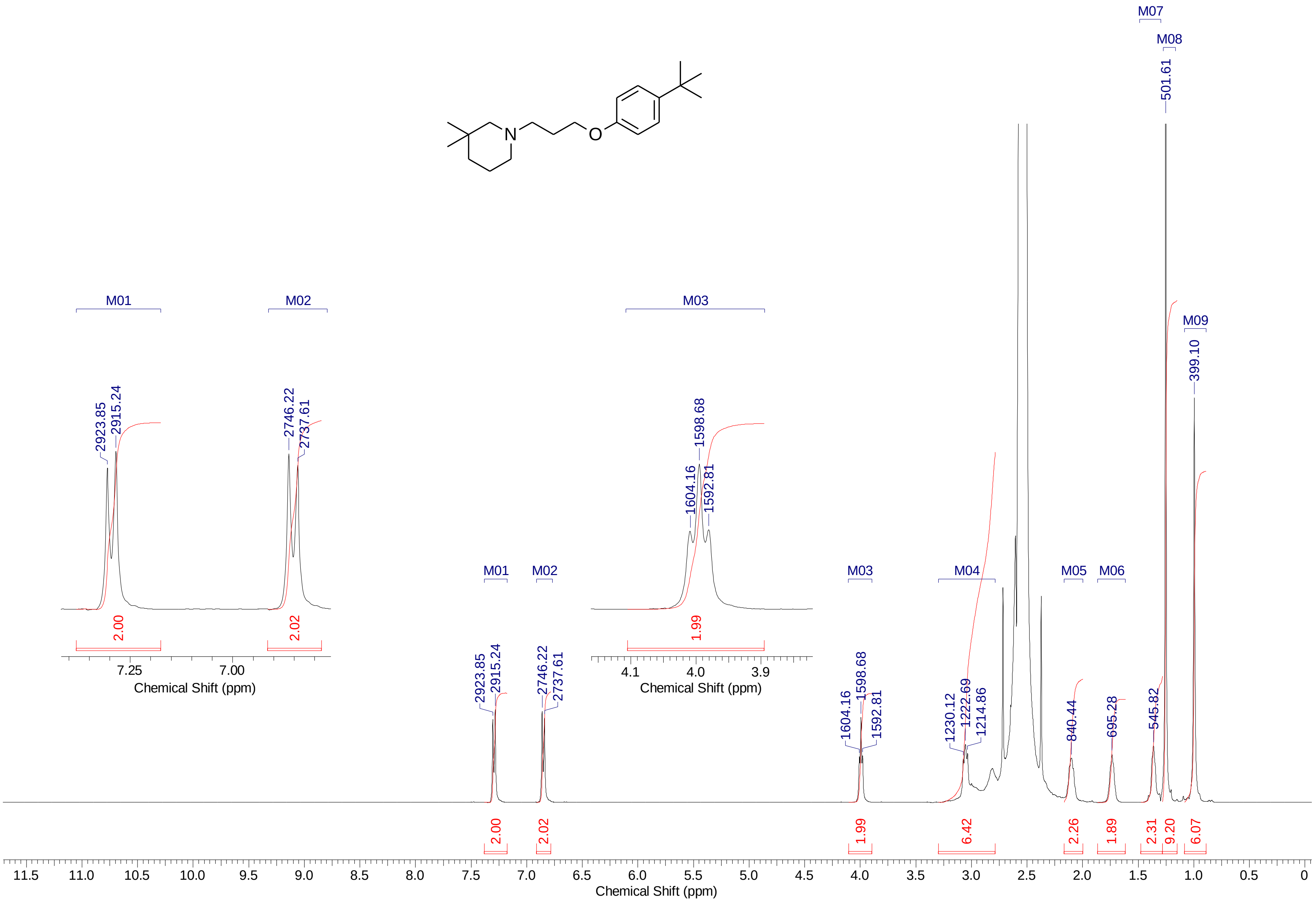

Fig S23. ^{1}H NMR spectrum of **16**

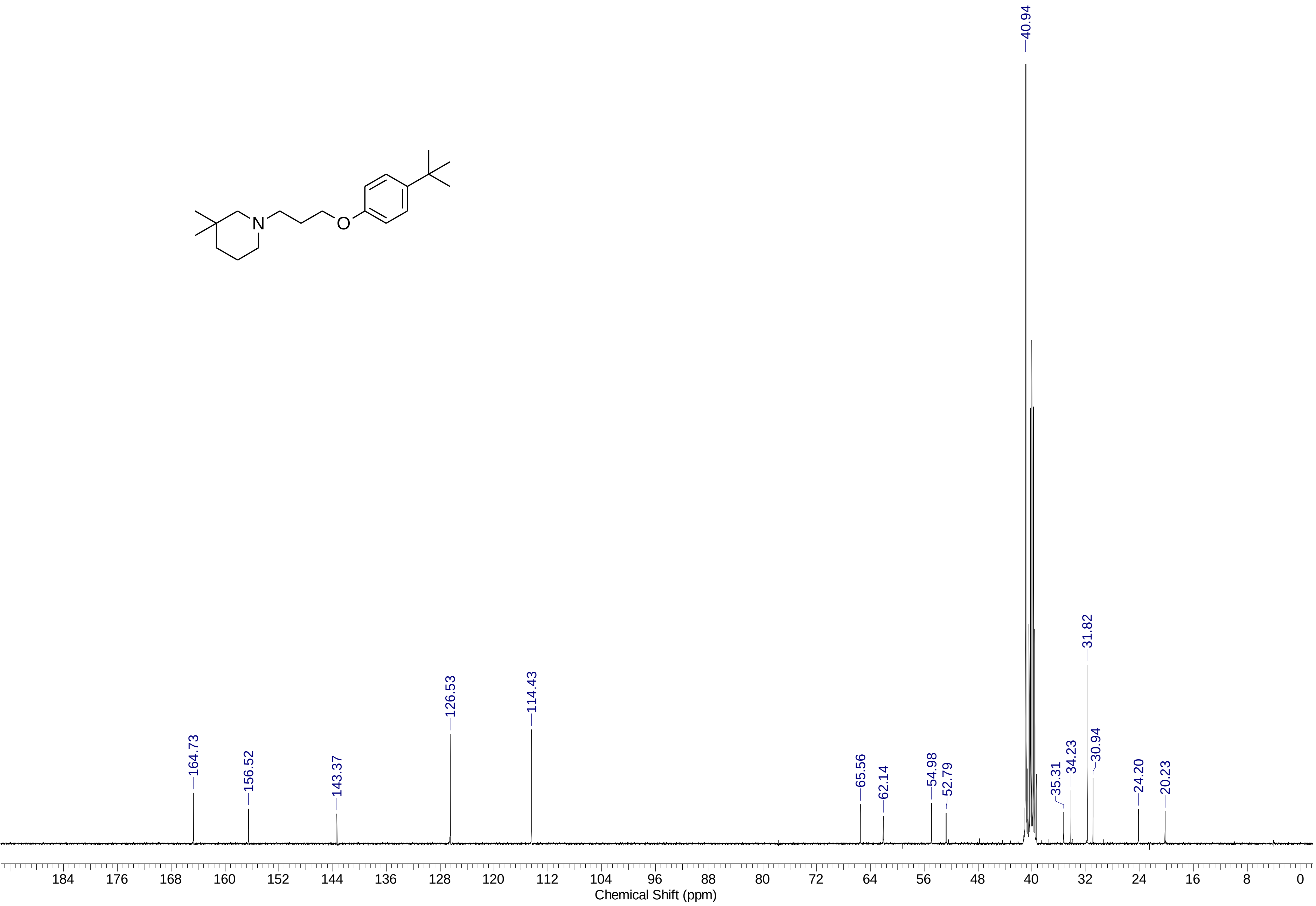

Fig S24. ^{13}C NMR spectrum of **16**

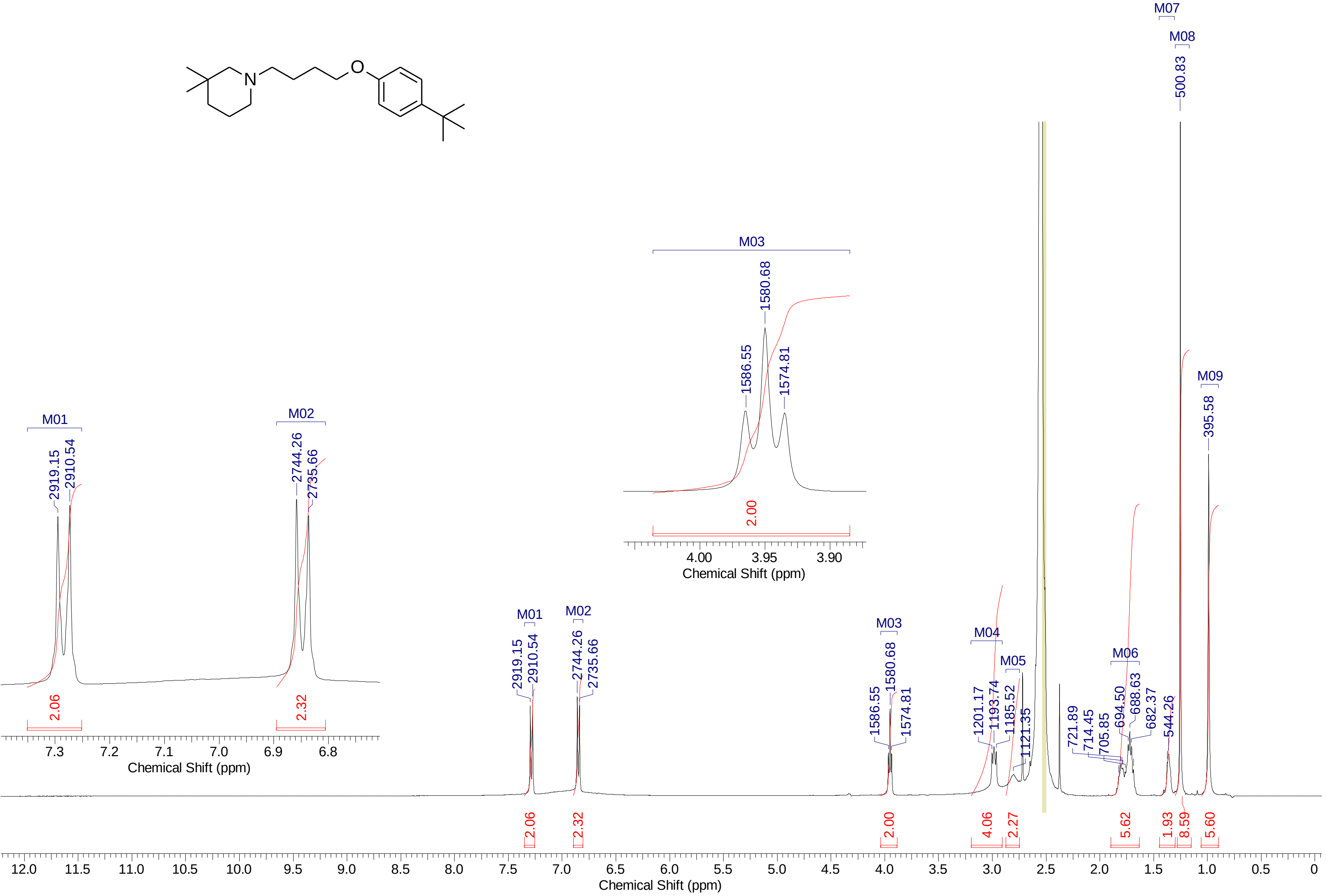

Fig S25. ^{1}H NMR spectrum of **17**

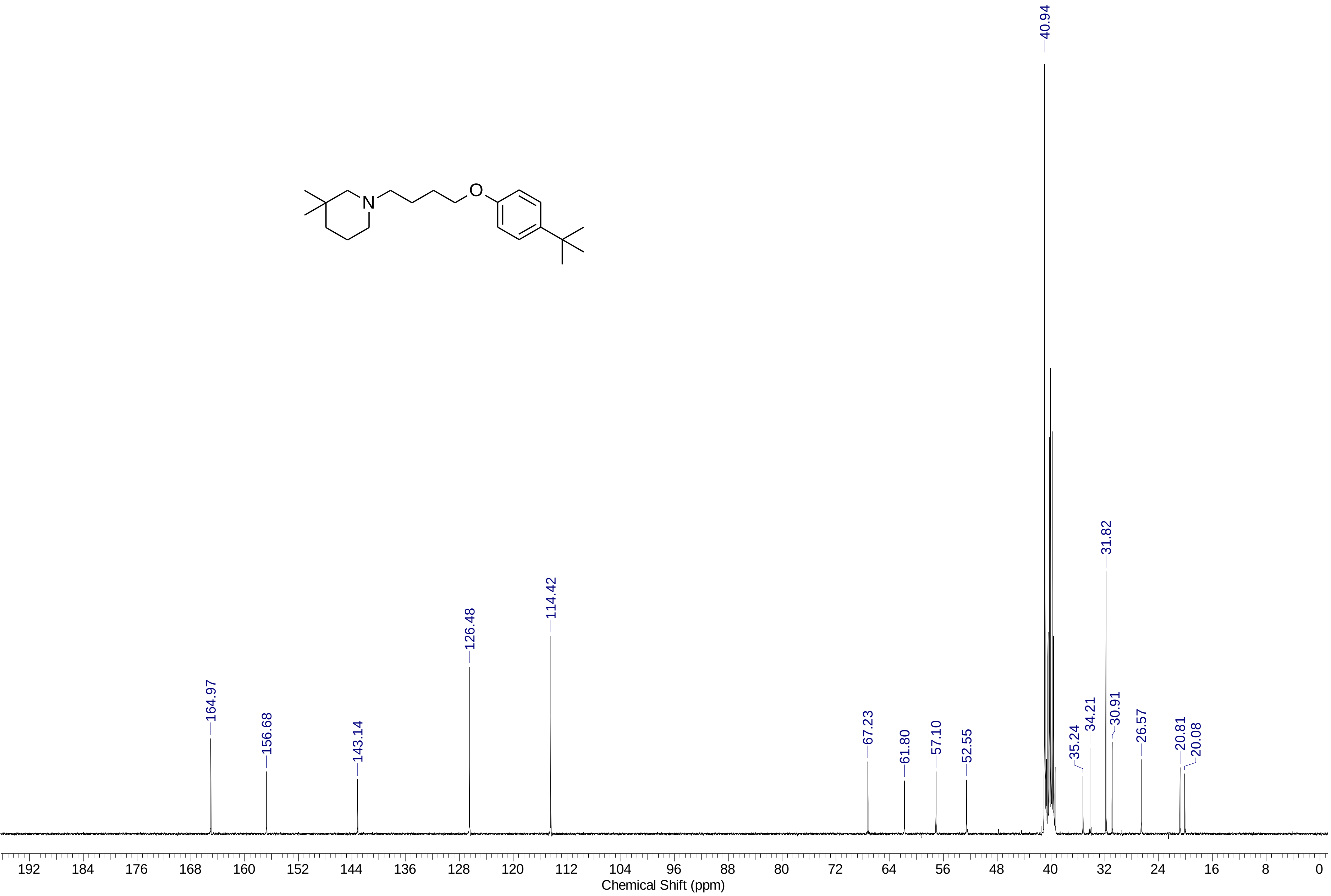

Fig S26. ^{13}C NMR spectrum of **17**

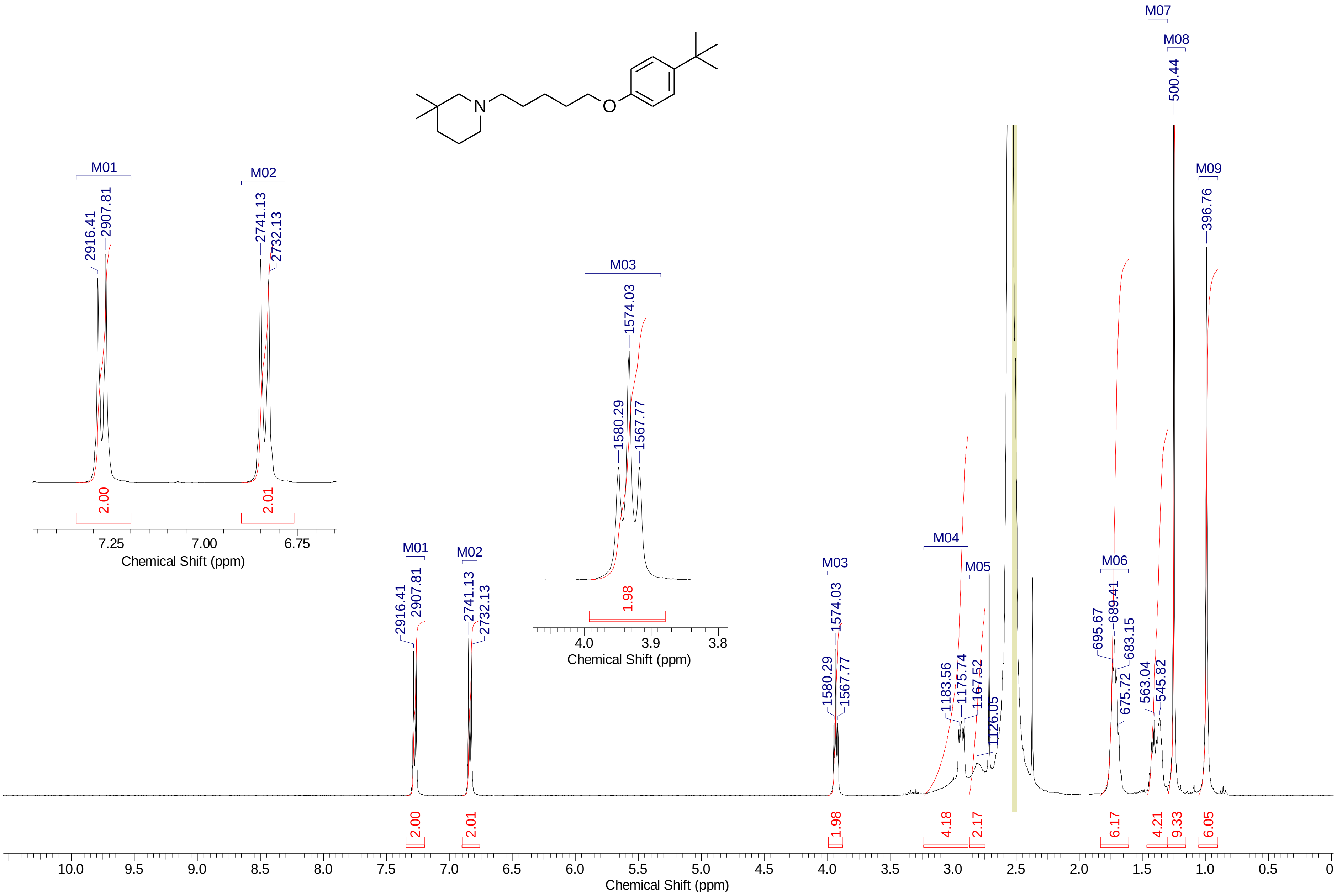

Fig S27. ^{1}H NMR spectrum of **18**

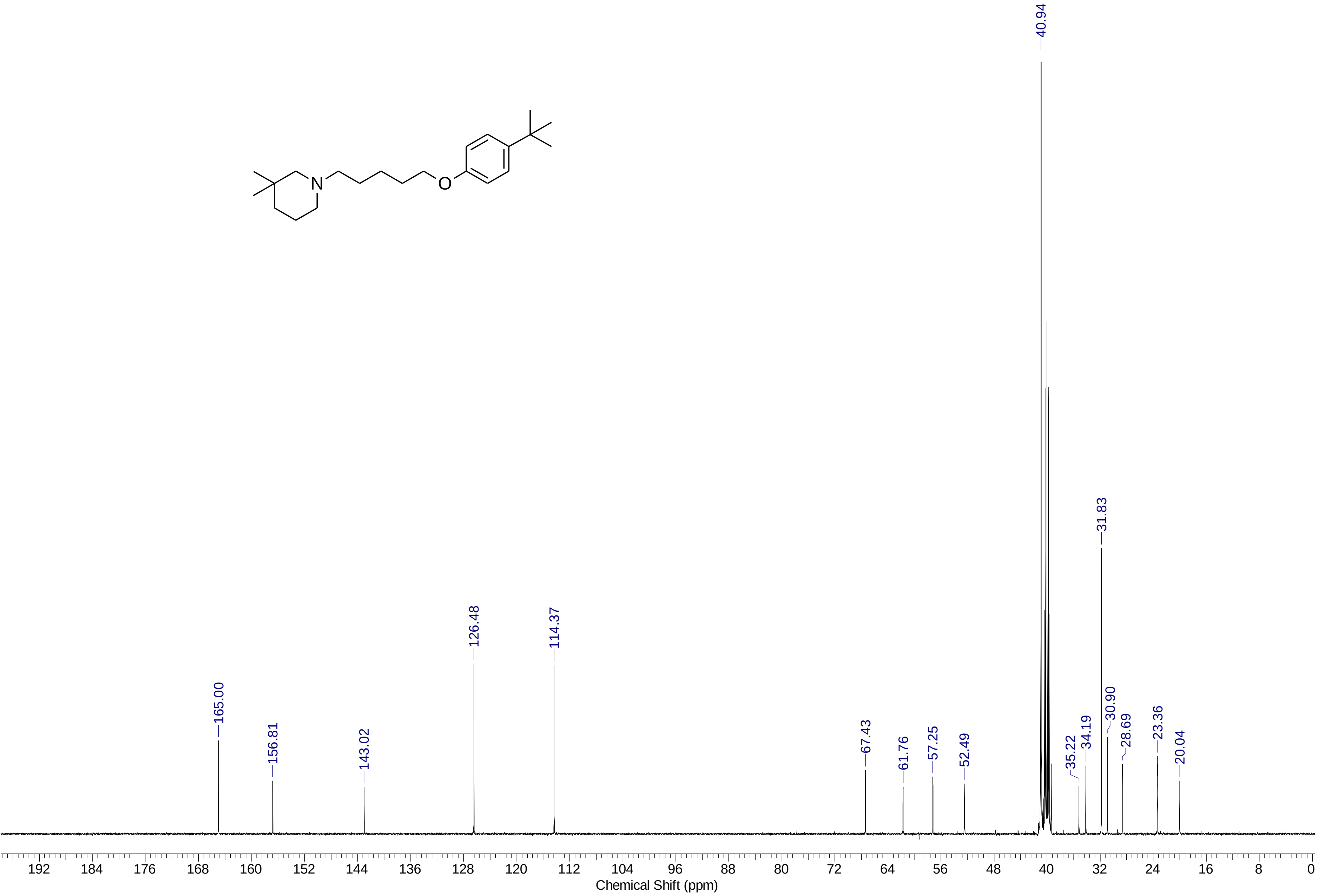

Fig S28. ^{13}C NMR spectrum of **18**

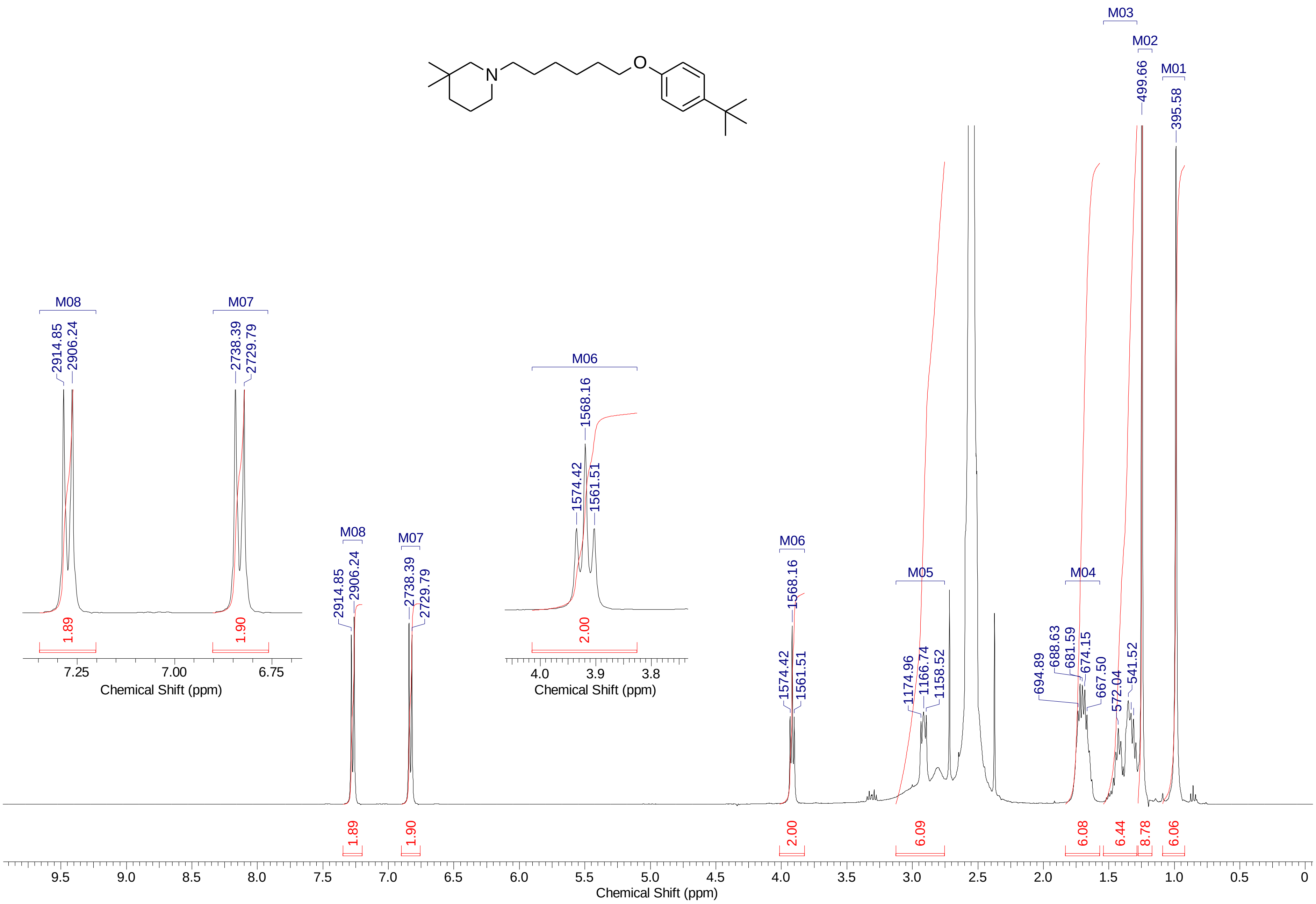

Fig S29. ^{1}H NMR spectrum of **19**

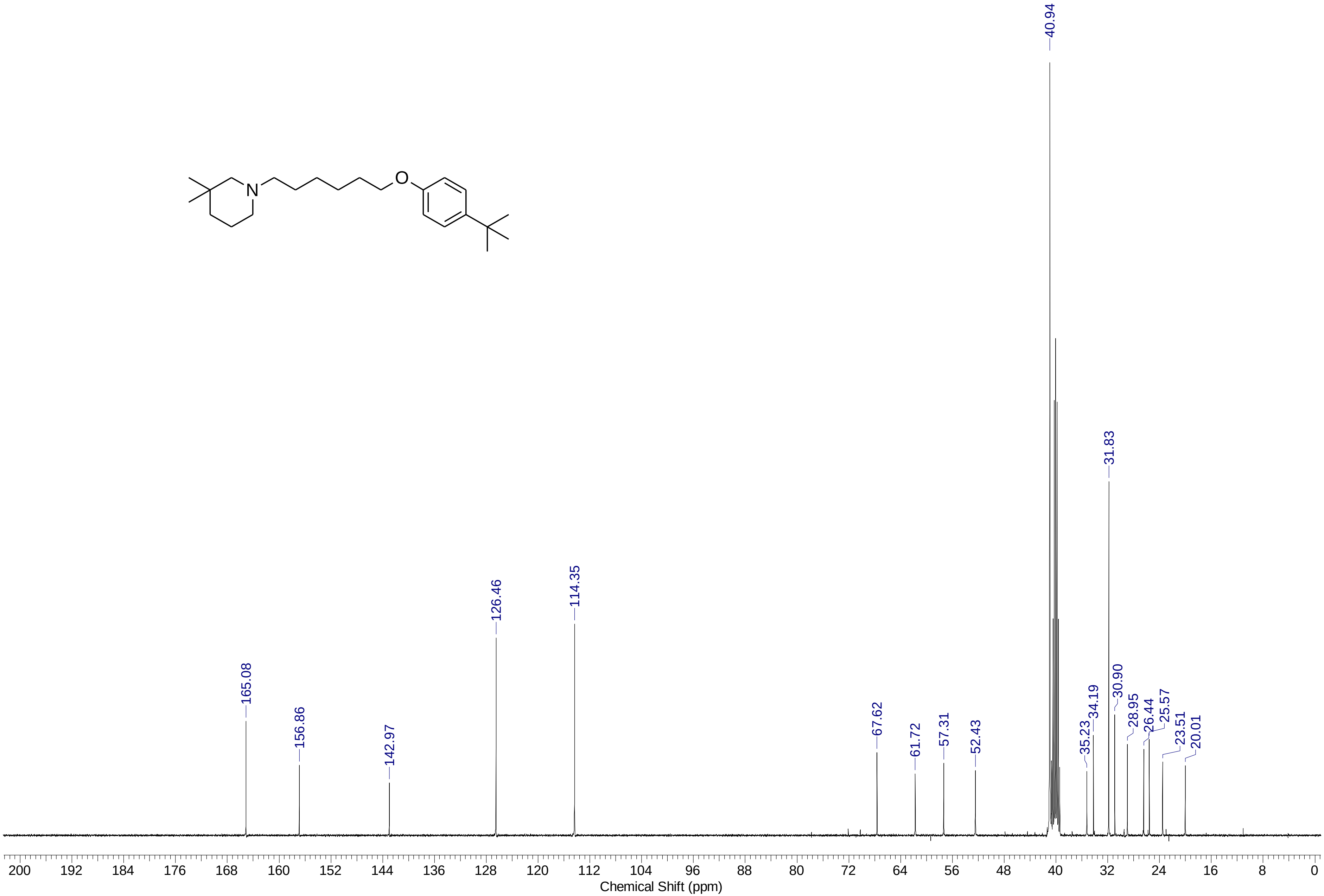

Fig S30. ^{13}C NMR spectrum of **19**

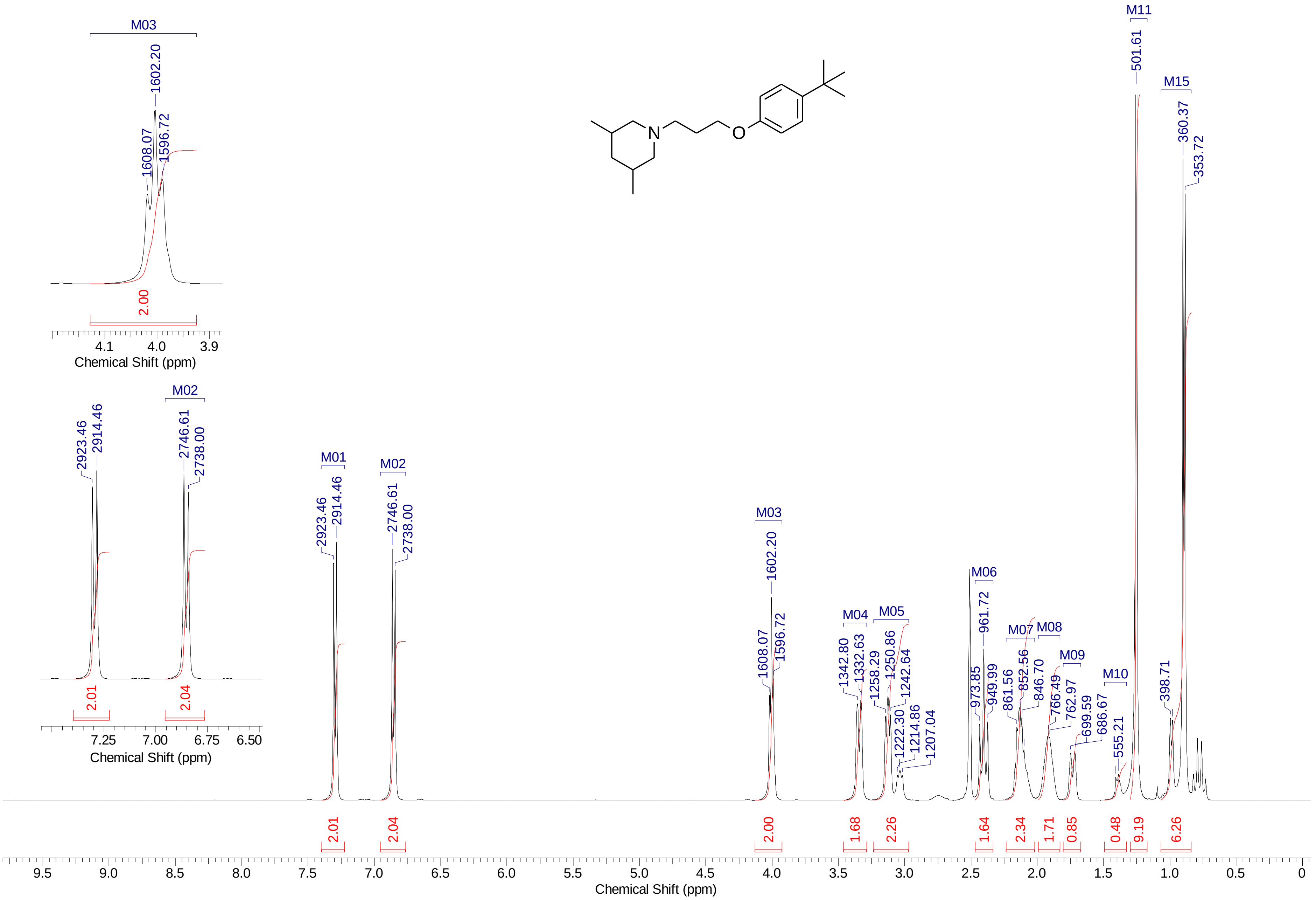

Fig S31. ^{1}H NMR spectrum of **20**

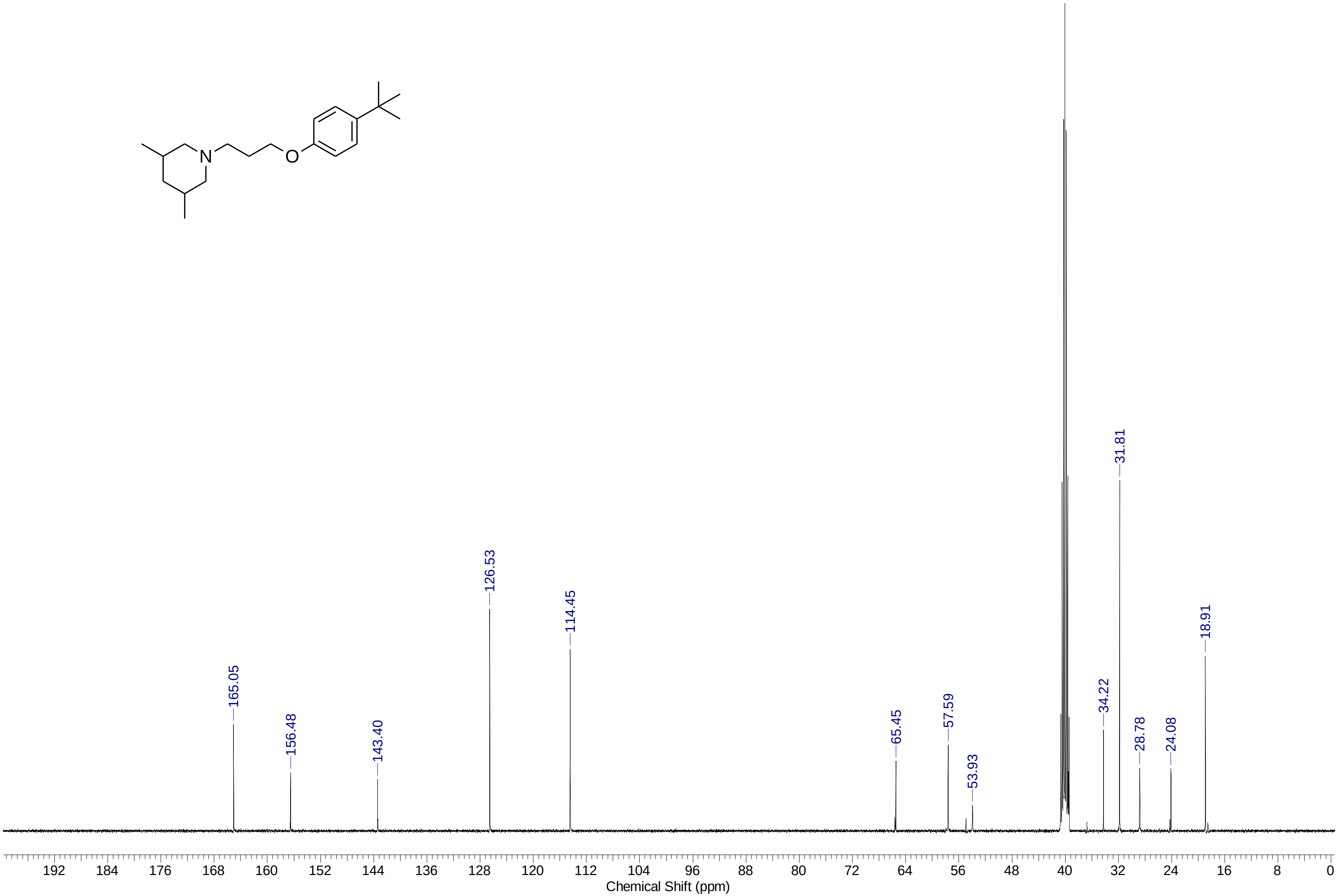

Fig S32. ^{13}C NMR spectrum of **20**

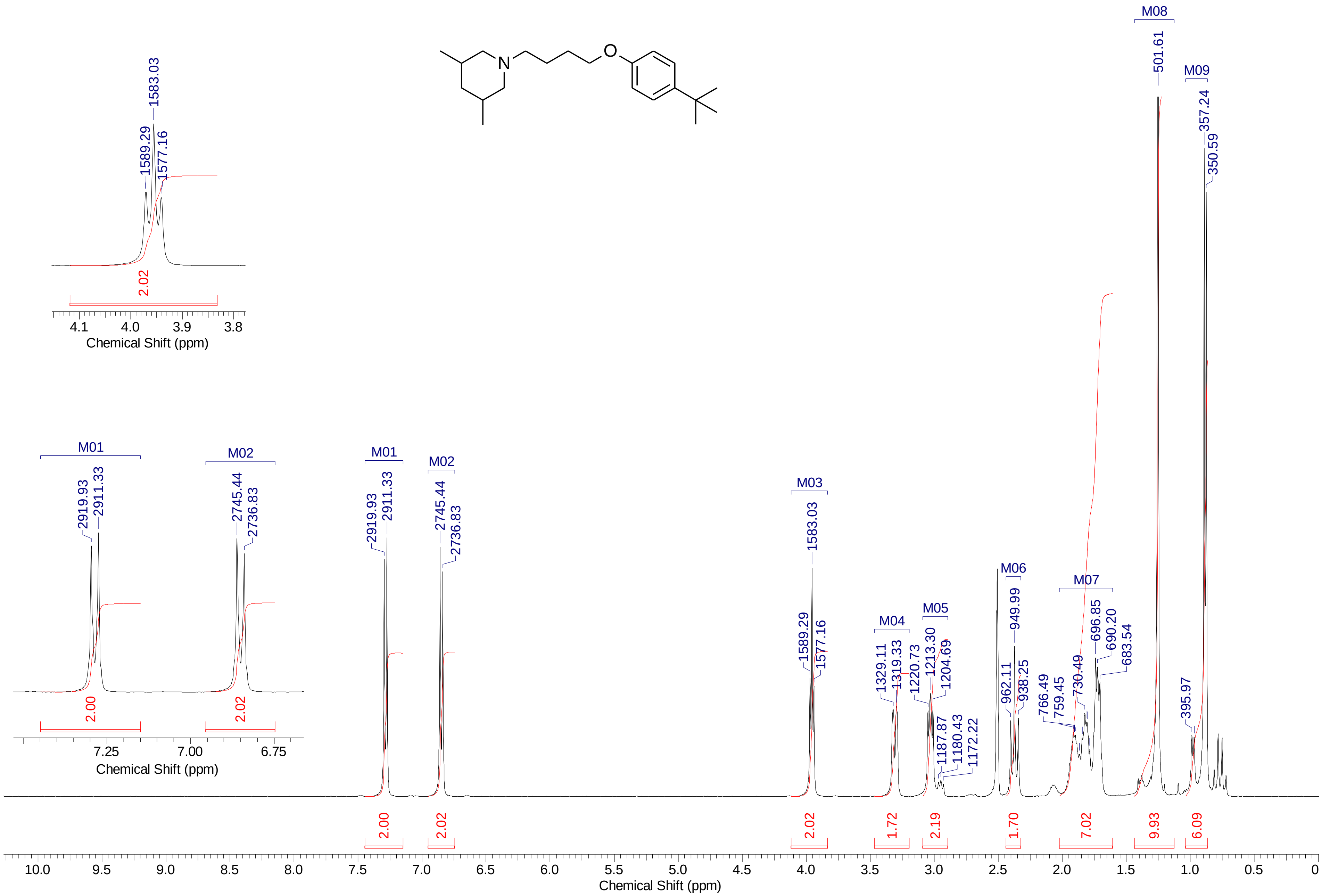

Fig S33. ^{1}H NMR spectrum of **21**

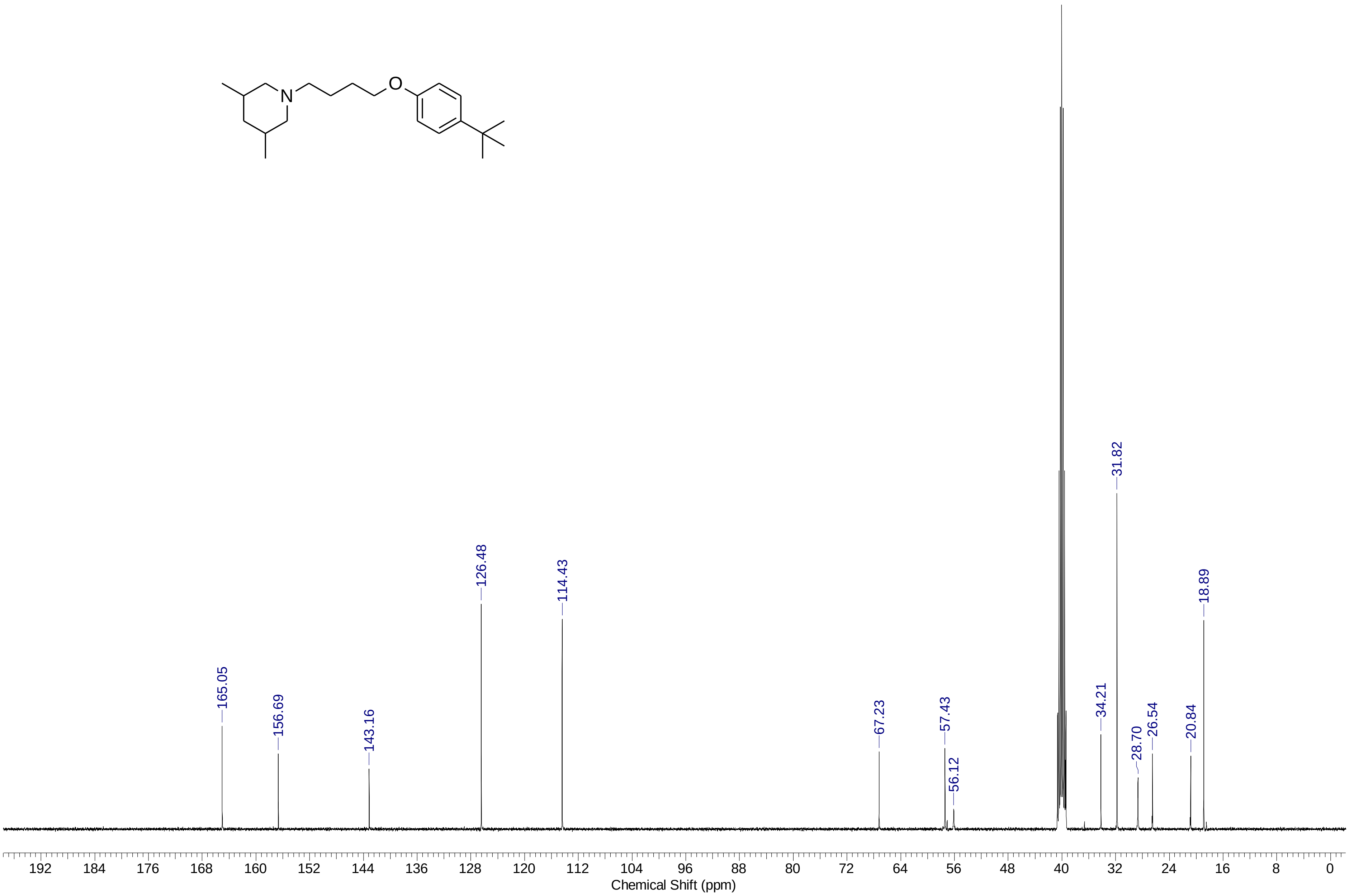

Fig S34. ^{13}C NMR spectrum of **21**

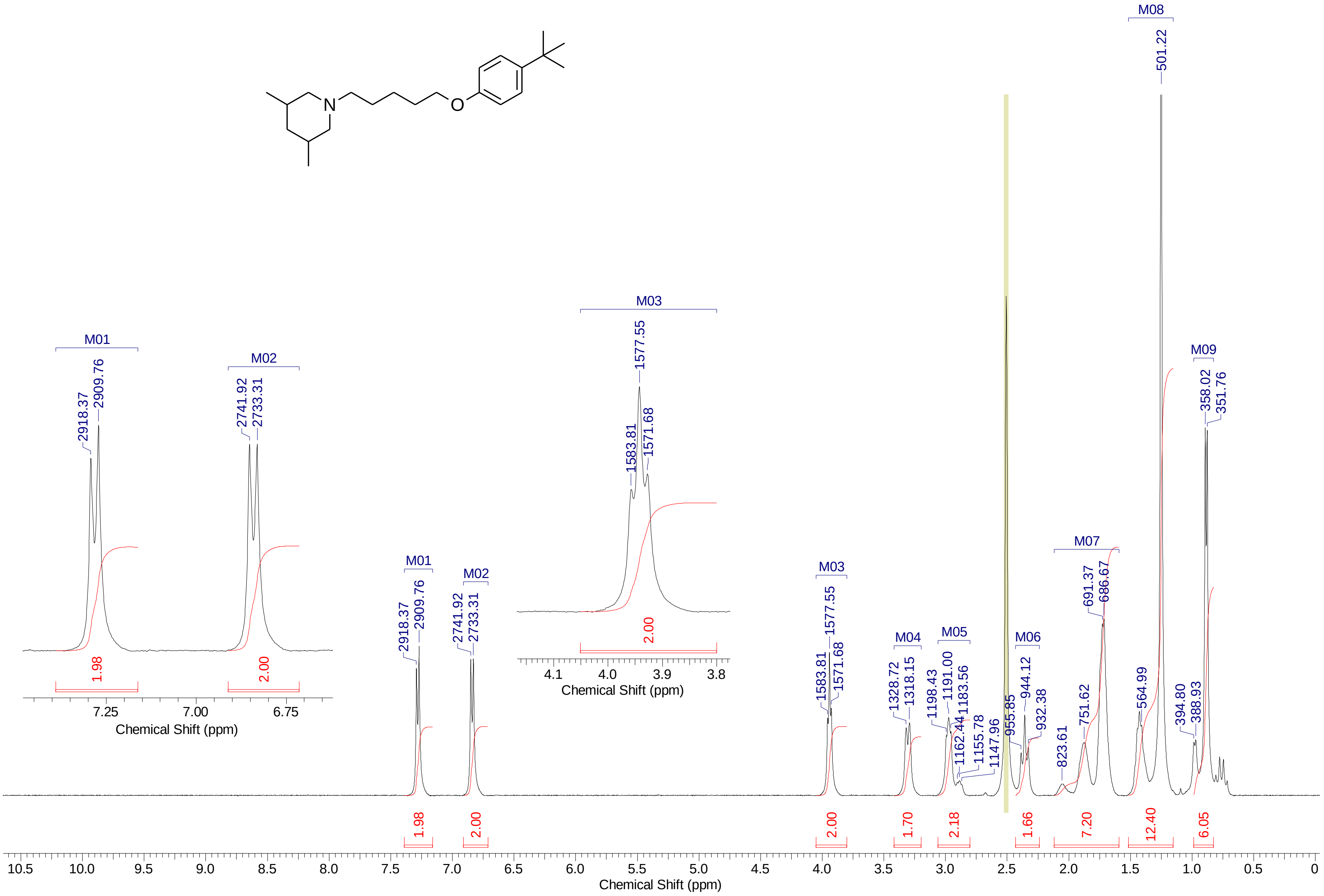

Fig S35. ^{1}H NMR spectrum of **22**

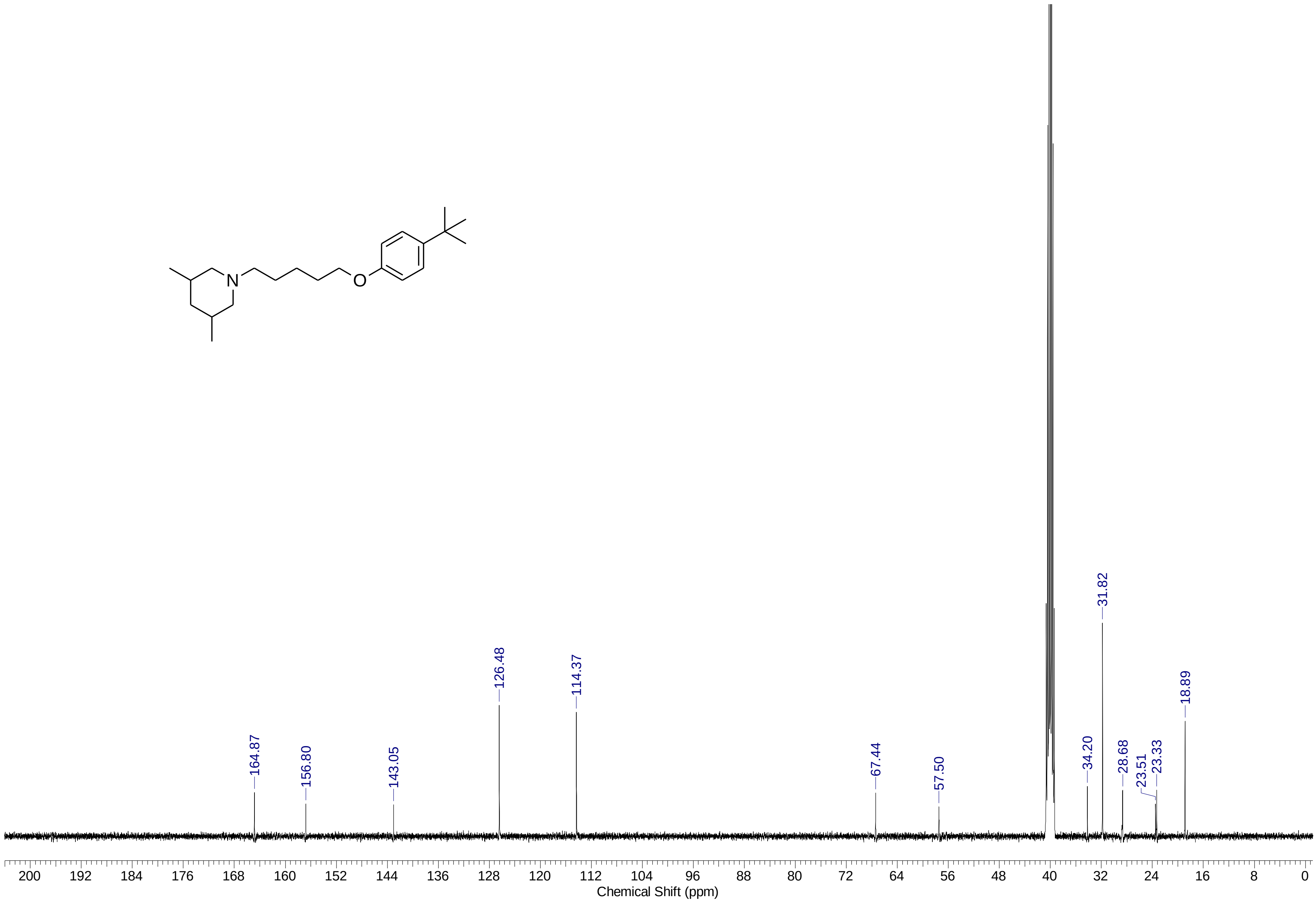

Fig S36. ^{13}C NMR spectrum of **22**

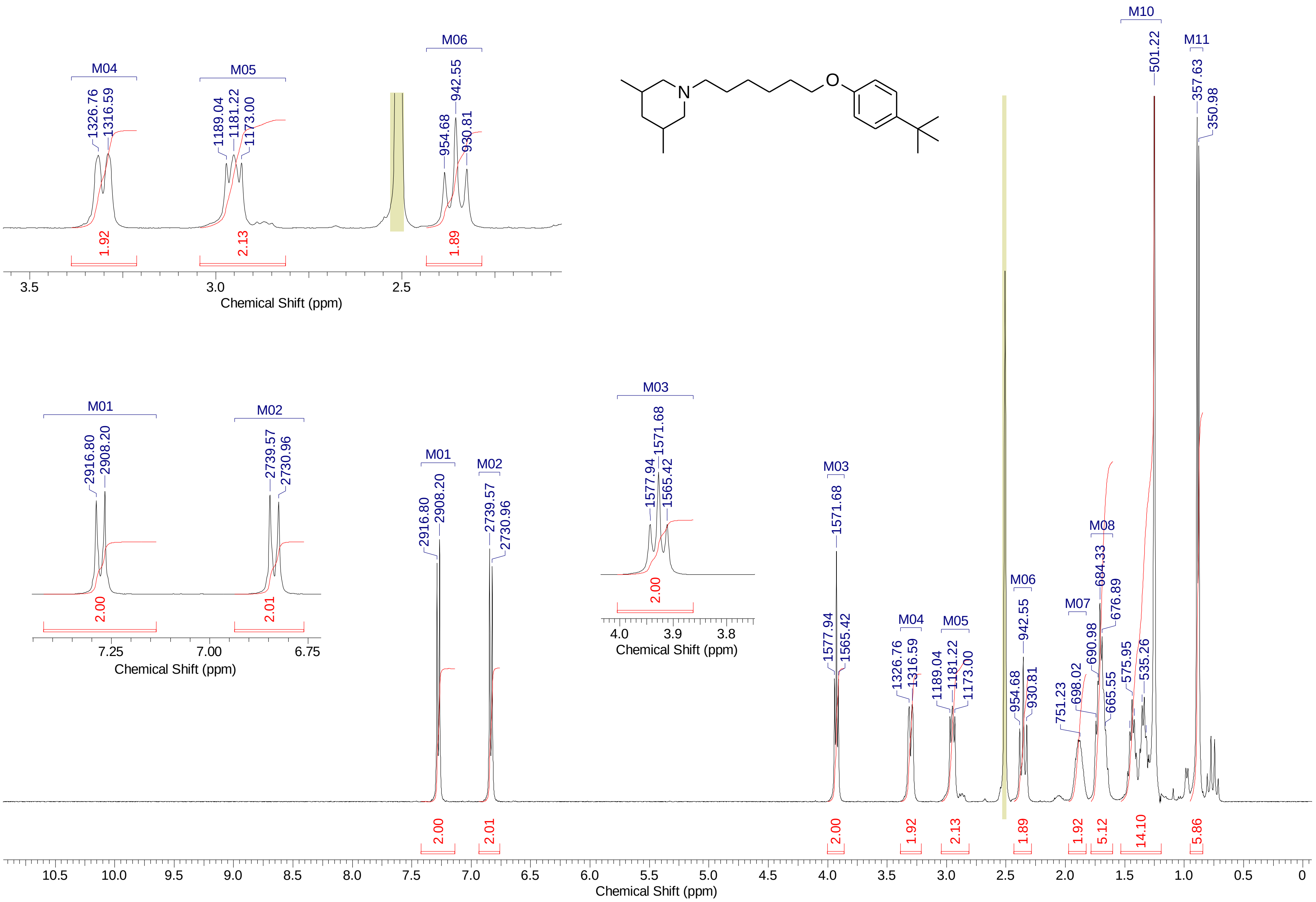

Fig S37. ^{1}H NMR spectrum of **23**

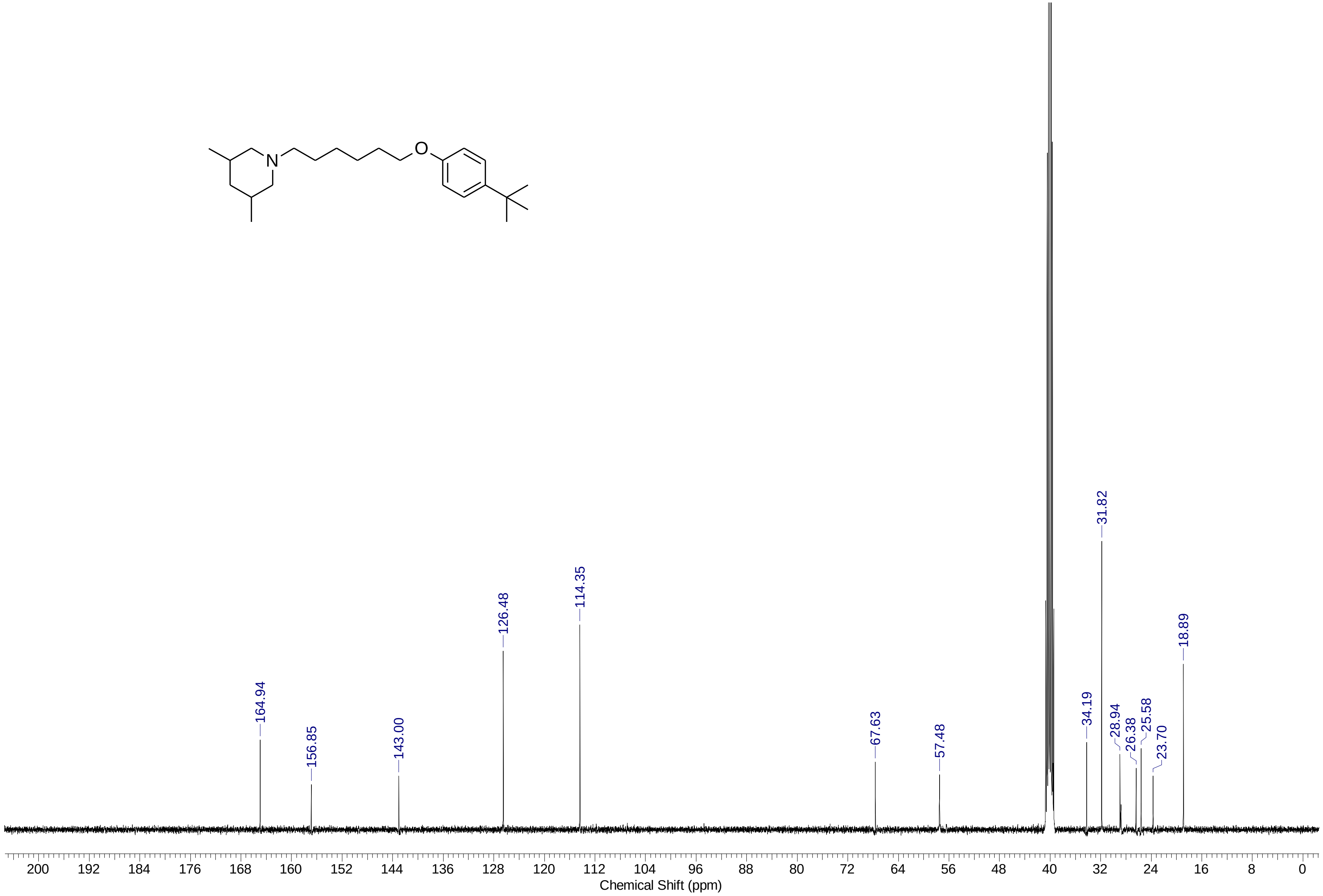

Fig S38. ^{13}C NMR spectrum of **23**

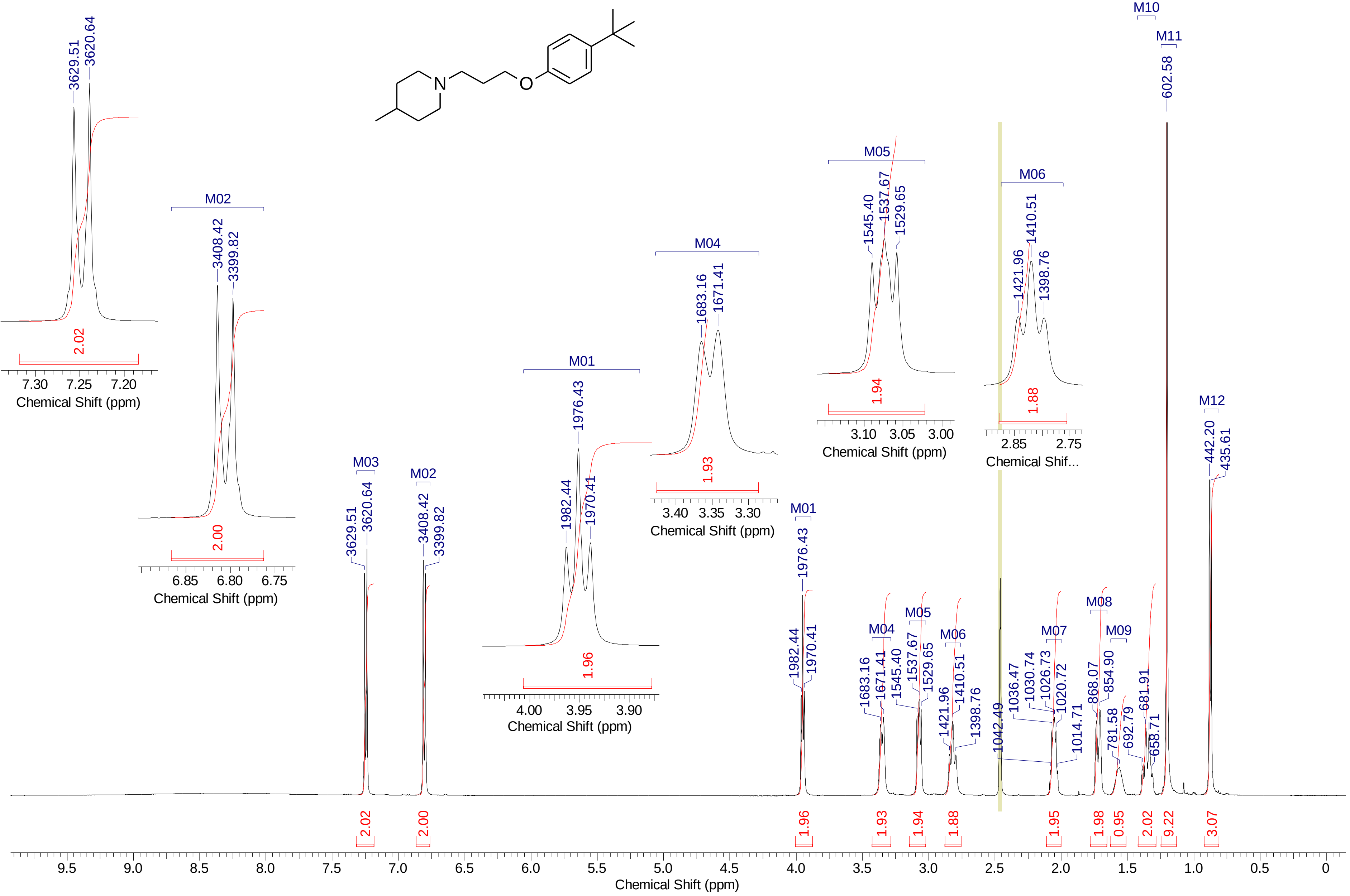

Fig S39. ^{1}H NMR spectrum of **24**

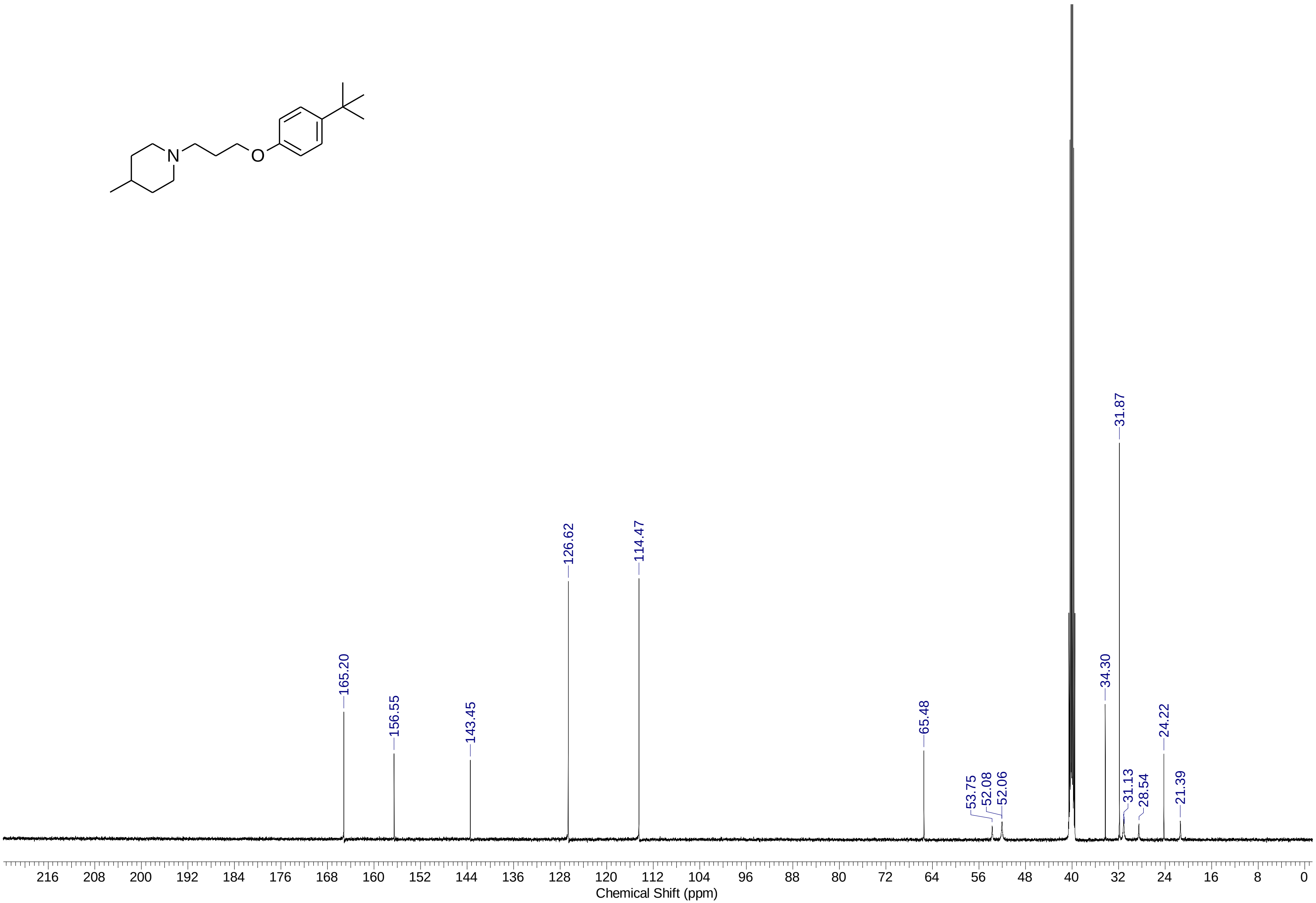

Fig S40. ^{13}C NMR spectrum of **24**

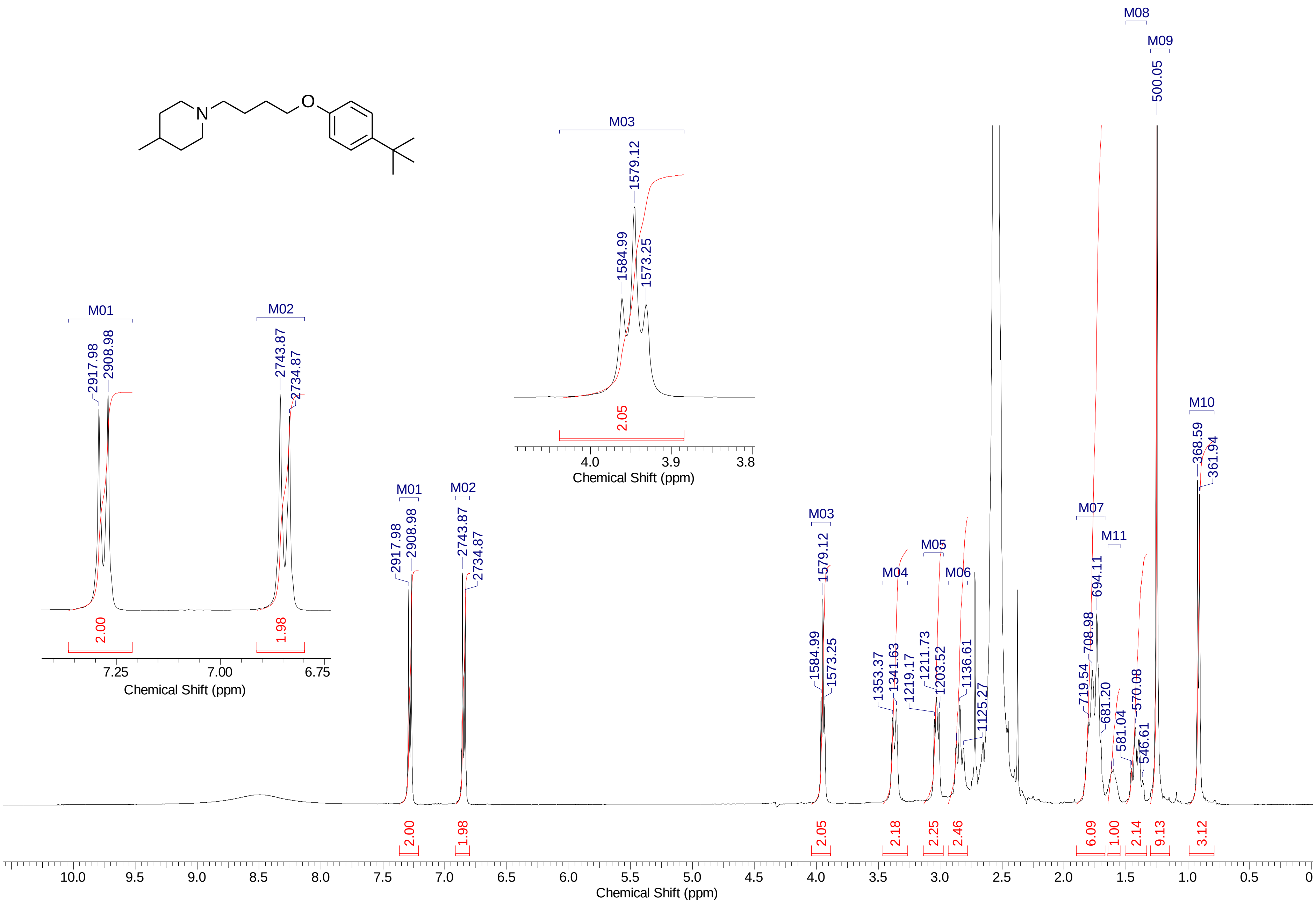

Fig S41. ^{1}H NMR spectrum of **25**

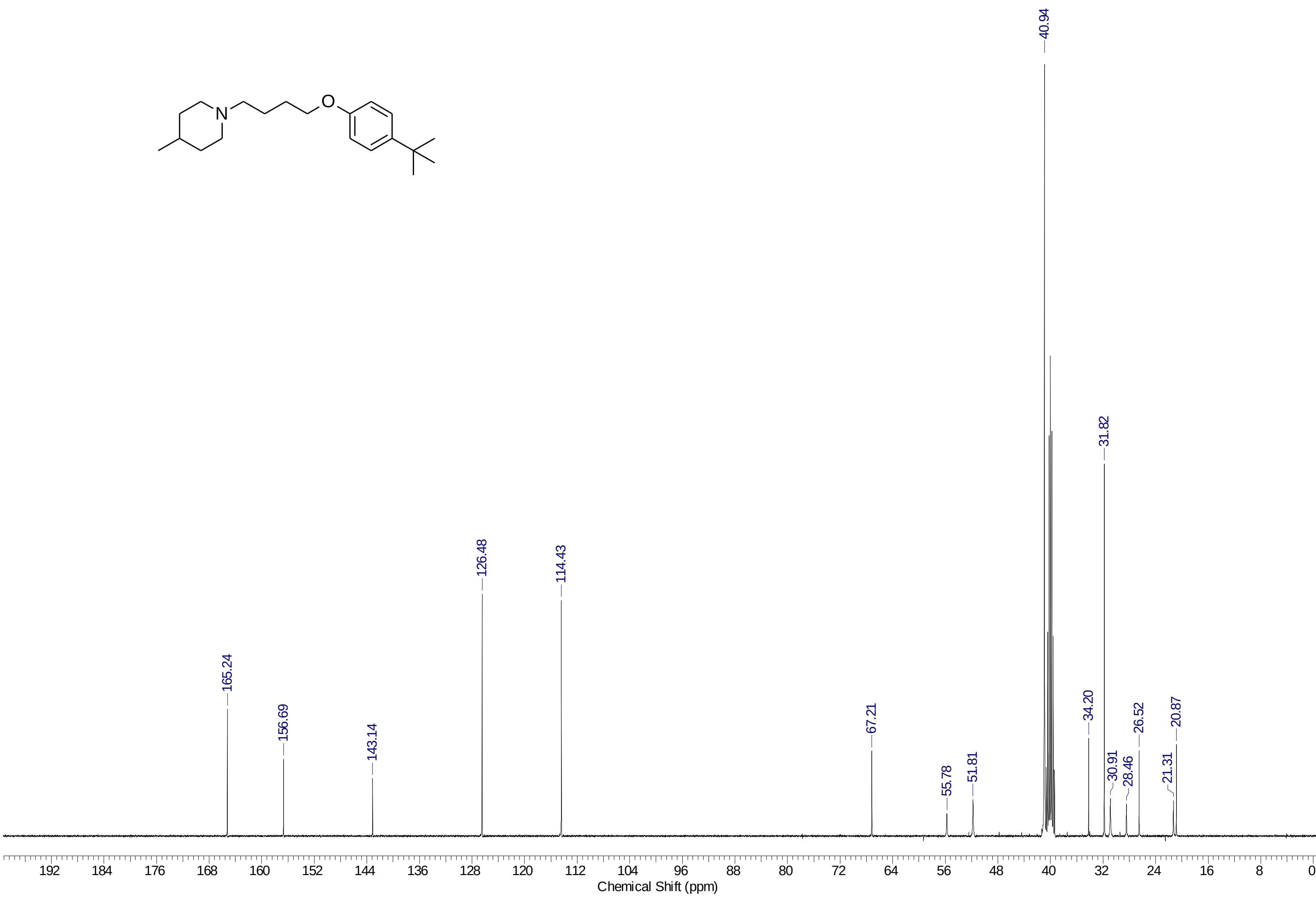

Fig S42. ^{13}C NMR spectrum of **25**

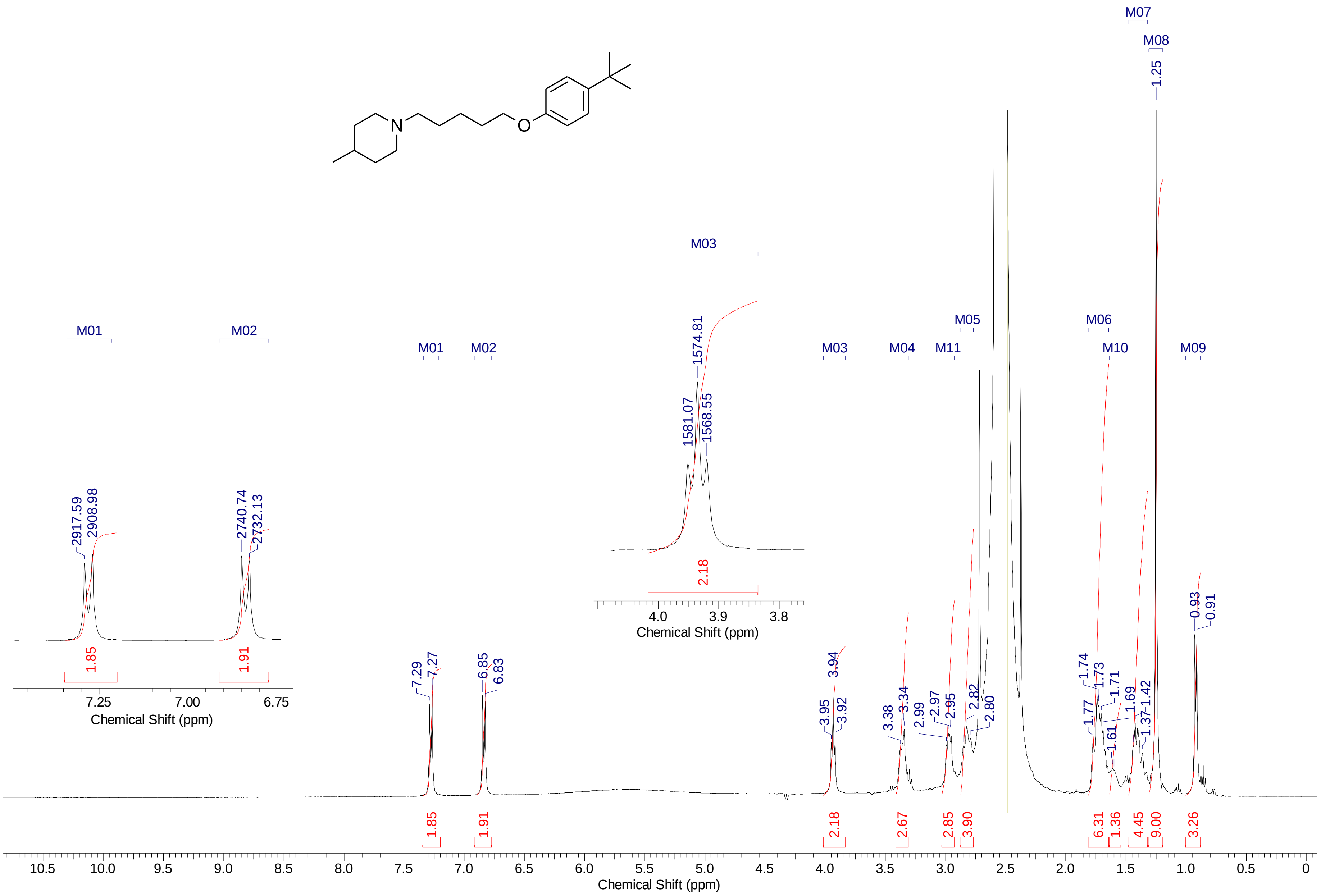

Fig S43. ^{1}H NMR spectrum of **26**

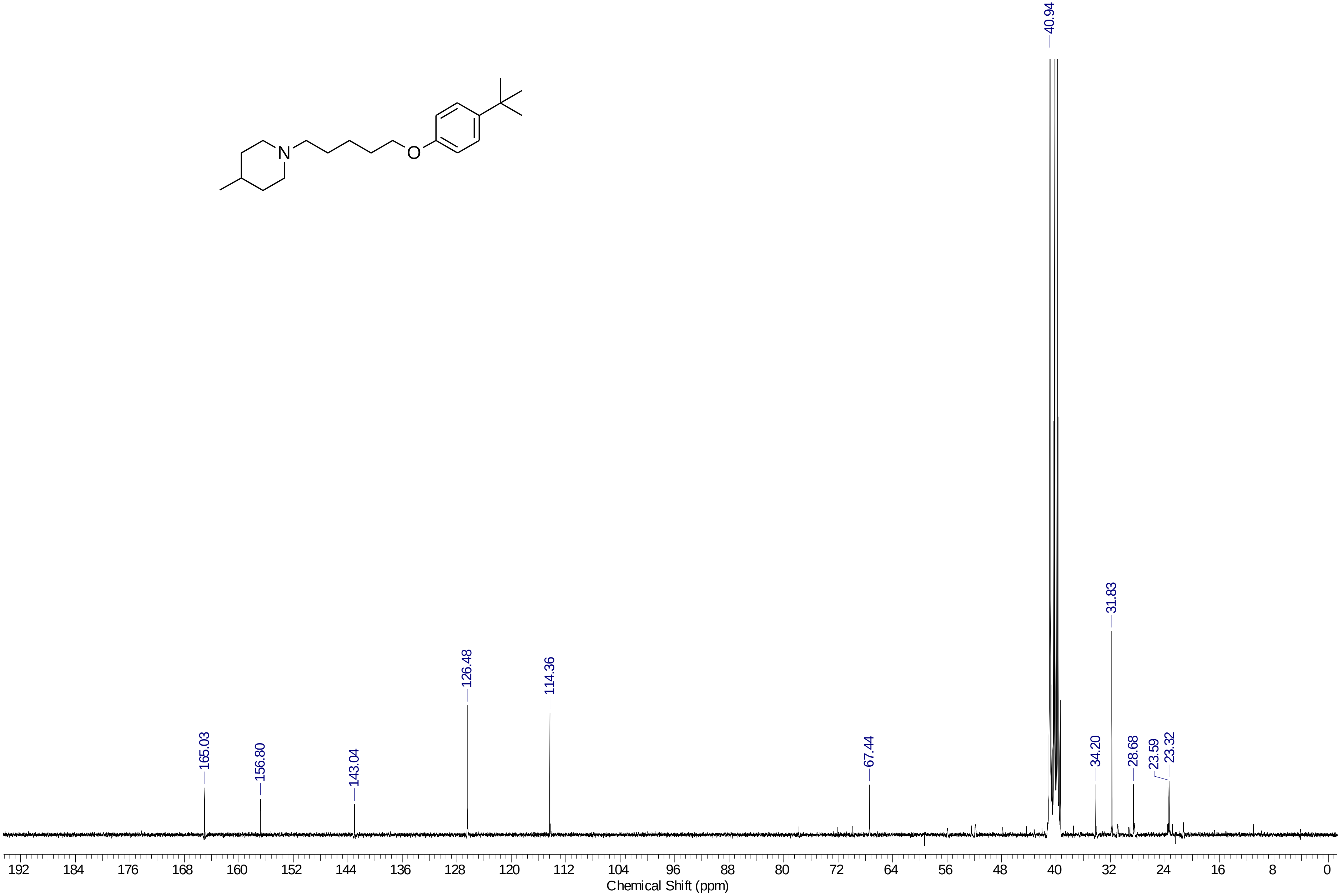

Fig S44. ^{13}C NMR spectrum of **26**

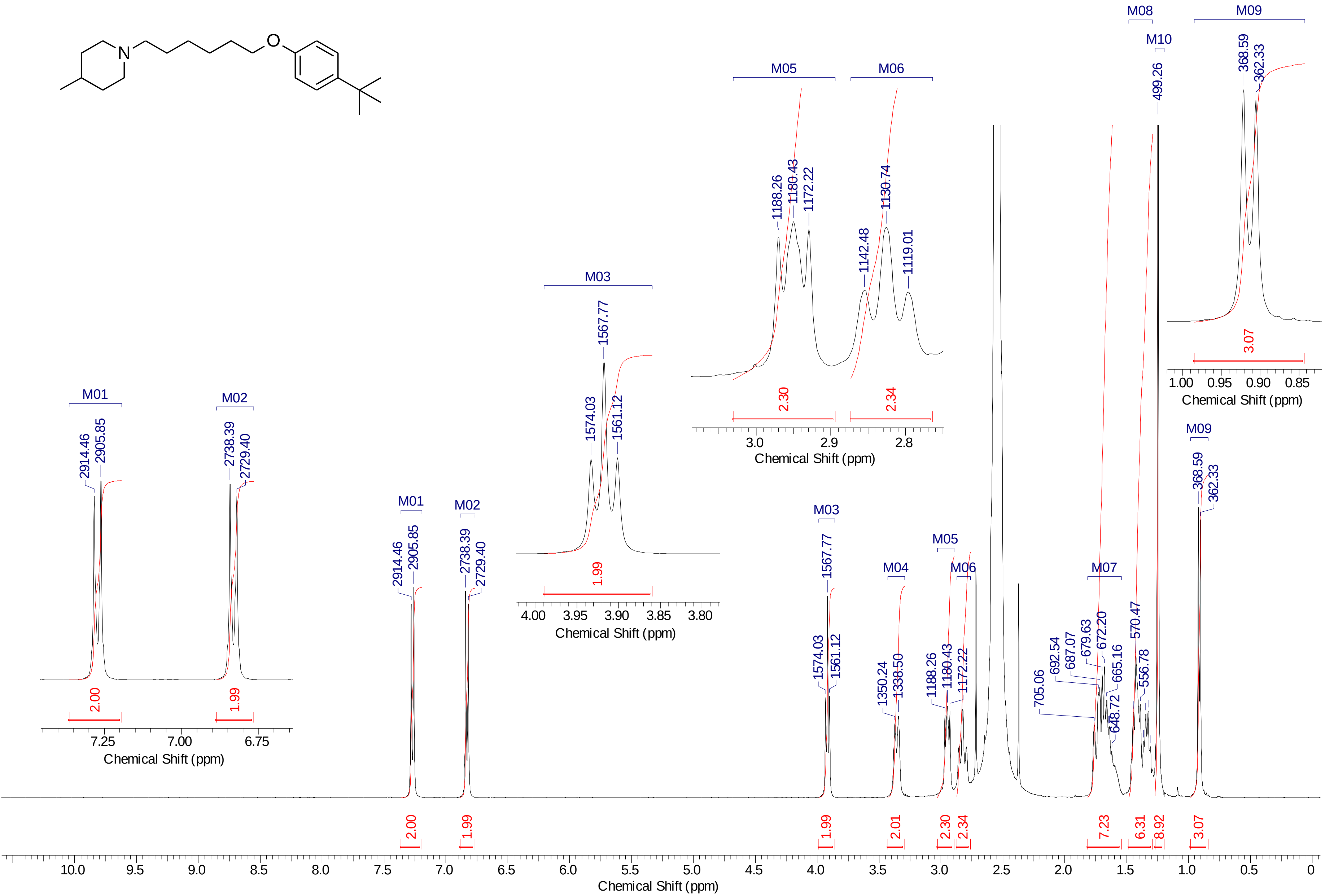

Fig S45. ^{1}H NMR spectrum of **27**

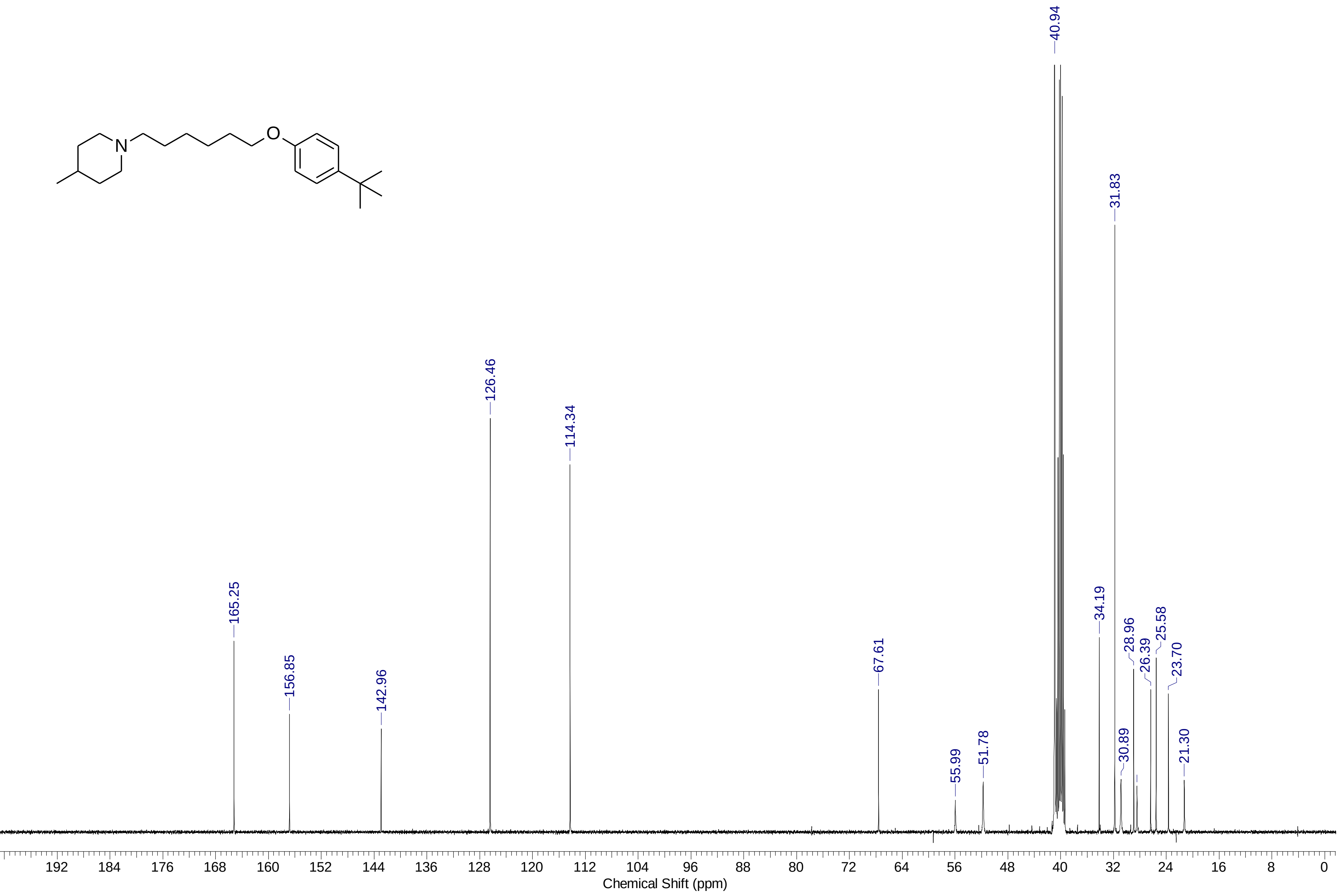

Fig S46. ^{13}C NMR spectrum of **27**

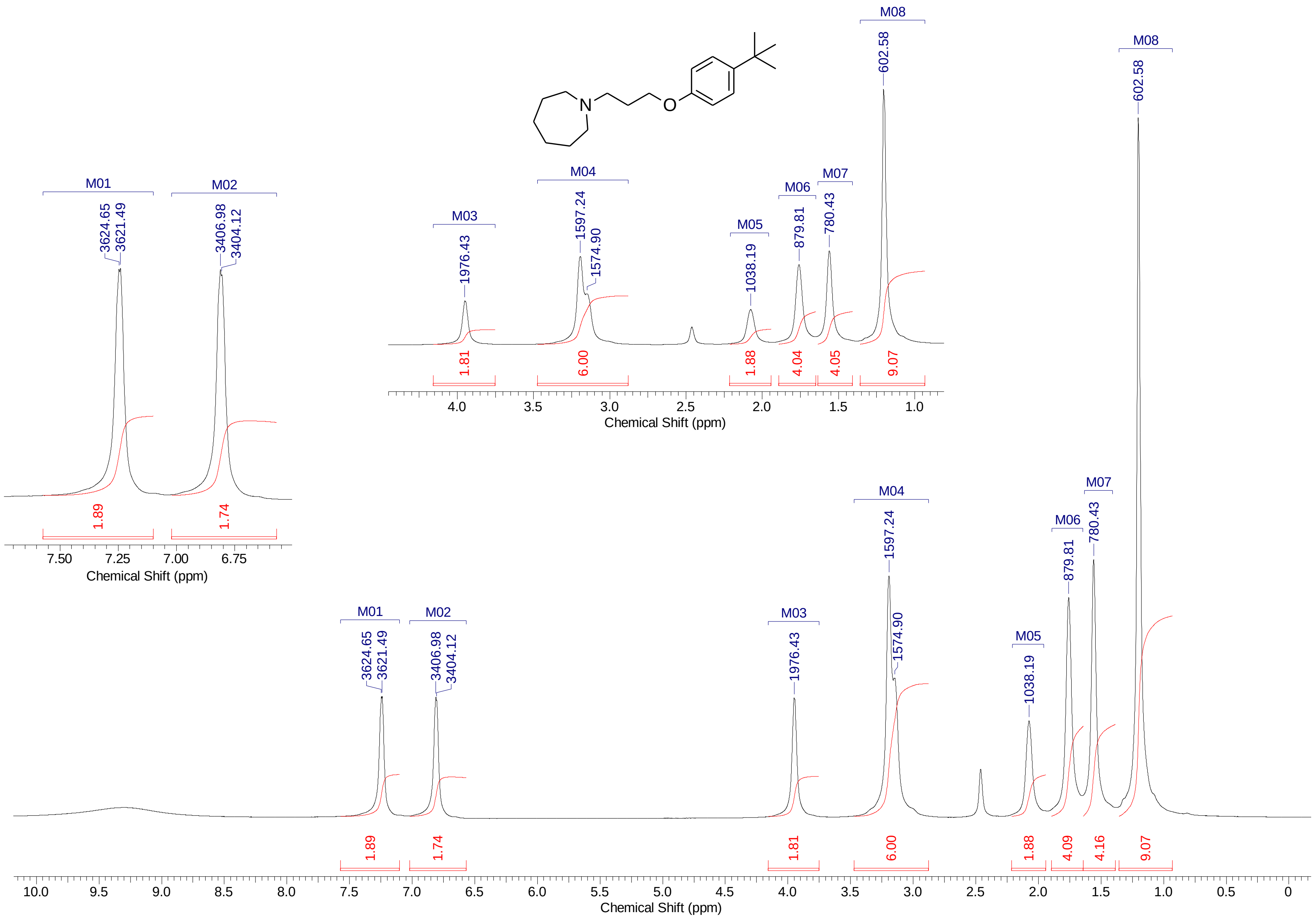

Fig S47. ^{1}H NMR spectrum of **28**

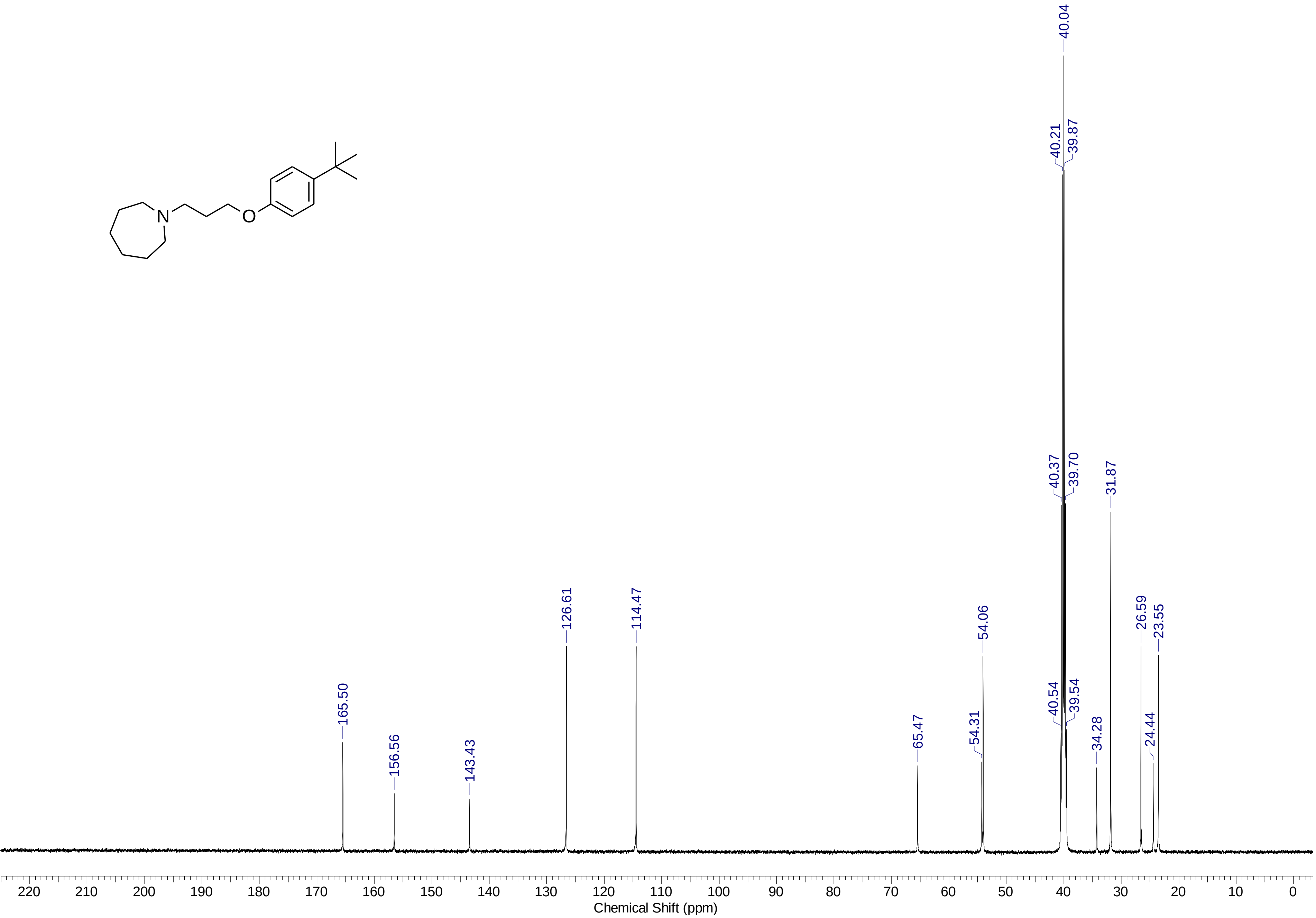

Fig S48. ^{13}C NMR spectrum of **28**

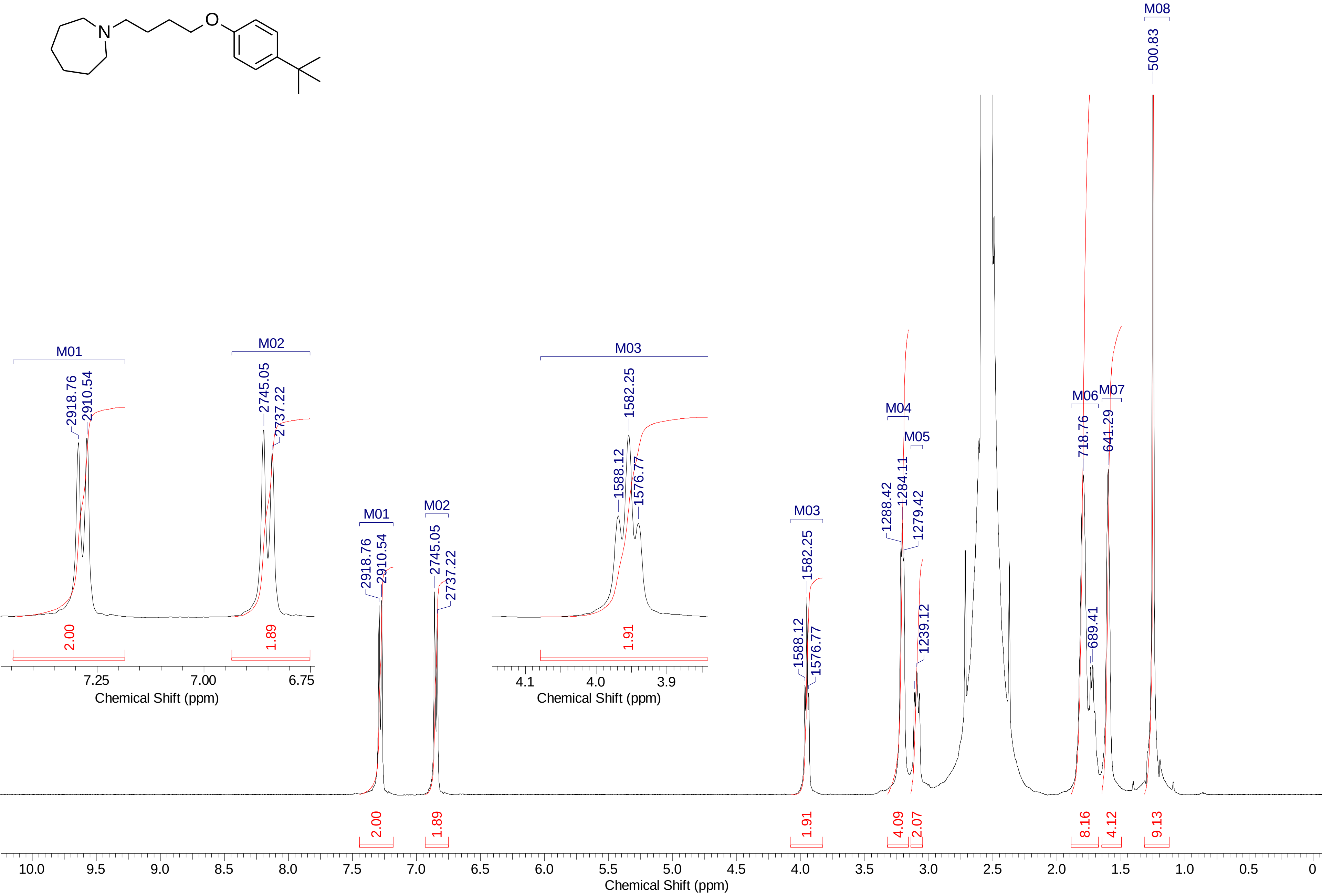

Fig S49. ^{1}H NMR spectrum of **29**

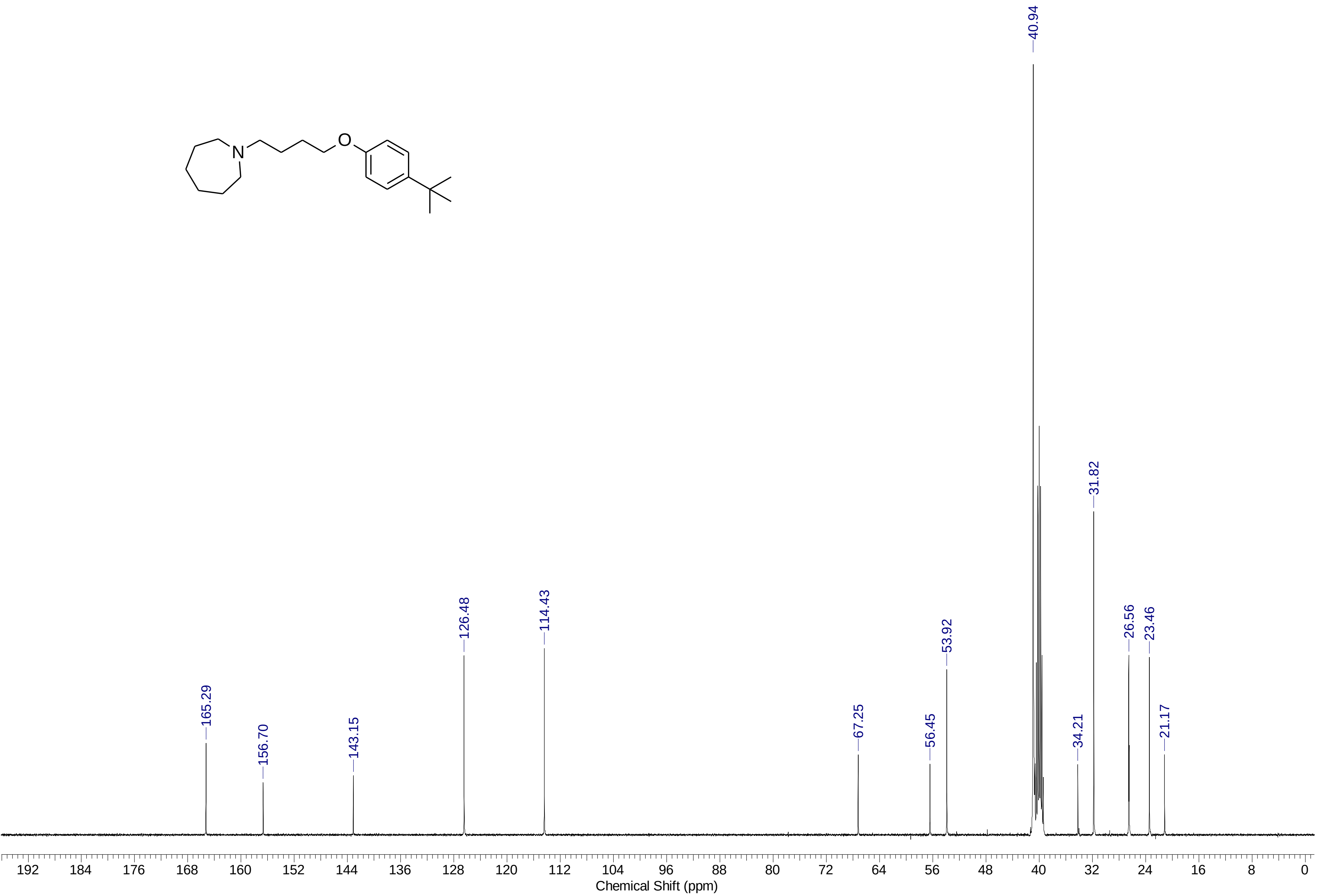

Fig S50. ^{13}C NMR spectrum of **29**

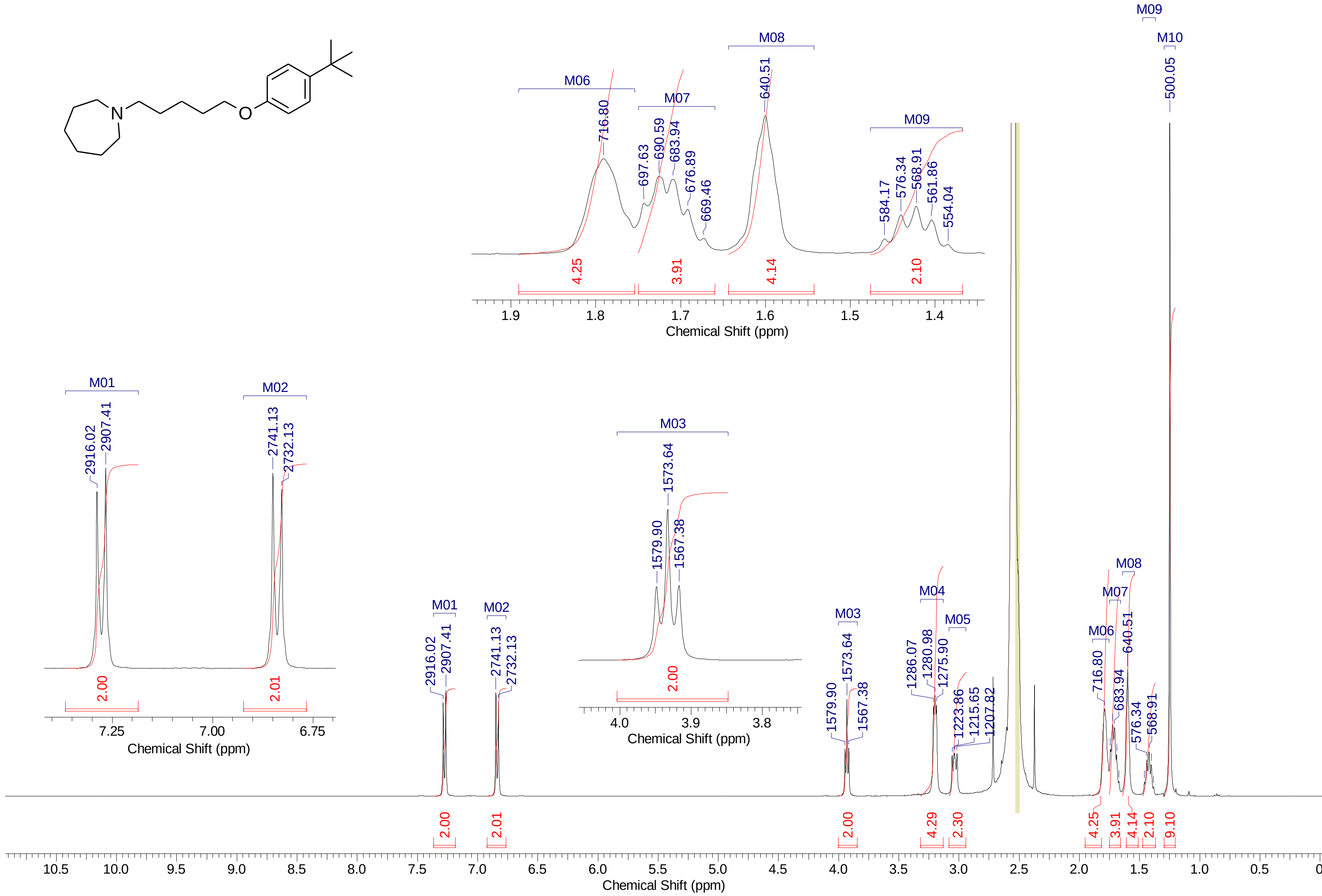

Fig S51. ^{1}H NMR spectrum of **30**

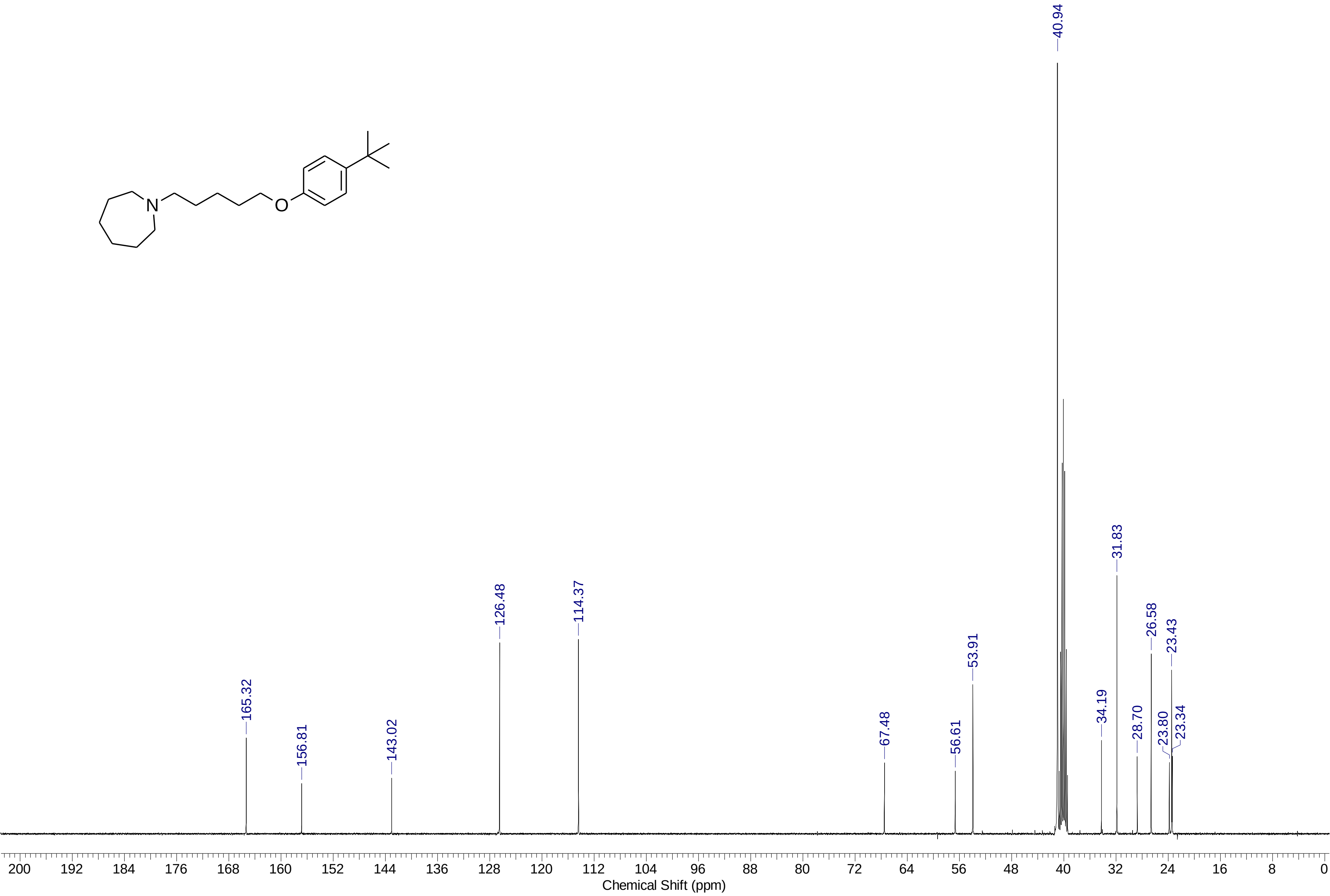

Fig S52. ^{13}C NMR spectrum of **30**

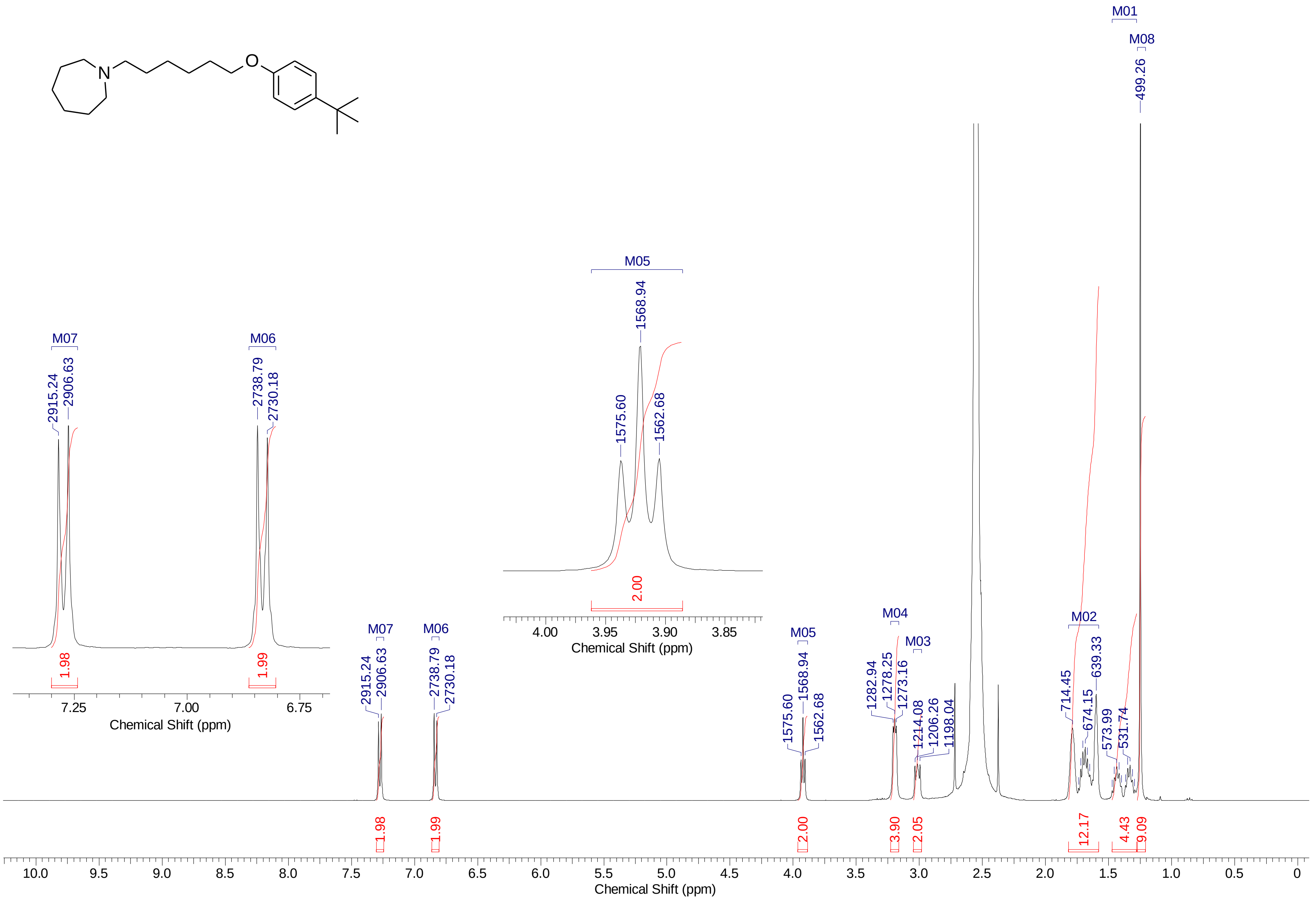

Fig S51. ^{1}H NMR spectrum of **31**

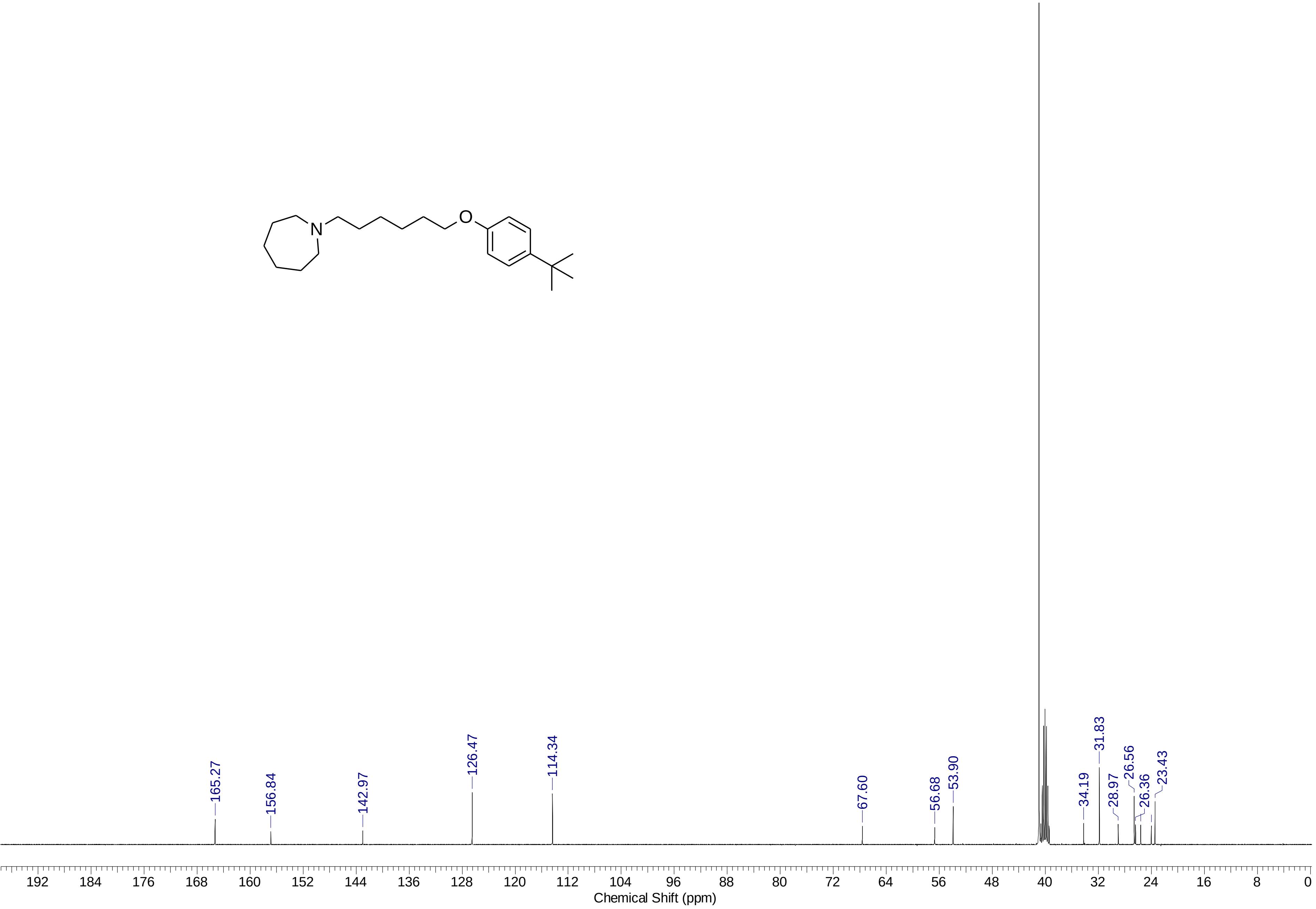

Fig S52. ^{13}C NMR spectrum of **31**
