# Supplementary material for: Dual Target Ligands with 4-tert-Butylphenoxy Scaffold as Histamine H3 Receptor Antagonists and Monoamine Oxidase B Inhibitors
Source: Int J Mol Sci. 2020 May 12;21(10):3411. doi: 10.3390/ijms21103411 (PMC7279487; doi:10.3390/ijms21103411)
Supplement: Supplementary file 1 [file ijms-21-03411-s001.zip › ijms-792322-Supplementary Materials/Supplementary Materials S2.pdf]

Supplementary Materials S2

Dual target ligands with 4-*tert*-butylphenoxy scaffold as histamine H_3 receptor antagonists and monoamine oxidase B inhibitors

Dorota Łażewska[1*], Agnieszka Olejarz-Maciej[1], David Reiner[2], Maria Kaleta[1], Gniewomir Latacz[1], Małgorzata Zygmunt[3], Agata Doroz-Płonka[1], Tadeusz Karcz[1], Annika Frank[2], Holger Stark[2] and Katarzyna Kieć-Kononowicz[1*]

[1] Department of Technology and Biotechnology of Drugs, Jagiellonian University Medical College, 9 Medyczna Str, 30-688 Kraków, Poland; agnieszka.olejarz@uj.edu.pl (A.O.-M.); maria.kaleta@uj.edu.pl (M.K.); glatacz@cm-uj.krakow.pl (G.L.); a.doroz-plonka@uj.edu.pl (A.D.-P.); t.karcz@uj.edu.pl (T.K.); mfkonono@cyf-kr.edu.pl (K.K.-K.)

[2] Institute of Pharmaceutical and Medicinal Chemistry, Heinrich Heine University Düsseldorf, Universitaets str. 1, 40225 Duesseldorf, Germany; david.reiner@hhu.de (D.R.); a.frank@hhu.de (A.F.); stark@hhu.de (H.S.)

[3] Department of Pharmacodynamics, Jagiellonian University Medical College, 9 Medyczna Str, 30-688, Kraków, Poland; malgorzata.zygmunt@uj.edu.pl (M.Z.)

* Correspondence: dlazewska@cm-uj.krakow.pl (D.Ł.); mfkonono@cyf-kr.edu.pl (K.K.-K.)

hMAO B Kinetic studies

The α values for different modes of reversible inhibition as well as their diagnostic signature on double-reciprocal plot are shown in Table S1. The α value for compound **9** was calculated by GraphPad Prism software from the nonlinear regression curves using following equation for mixed-model of inhibition:

$$v = V_{max}*[S] / [S]*(1+ [I]/ \alpha K_i) + K_M*(1+ [I]/K_i) \quad (1)$$

v – velocity of enzyme reaction. V_{max} - maximum velocity (at infinite substrate concentration); K_i – dissociation constant for Enzyme-Inhibitor complex (in the enzymology literature other symbols can be used such as: K_i, K_{ii} , K_{EI} etc.); αK_i – dissociation constant for Enzyme-Substrate-Inhibitor complex (in the literature also under the symbols: K_i', K_{is} and K_{ES}); K_M – Michaelis-Menten constant; [I]– concentration of the inhibitor; [S] – concentration of the substrate [Ref S1].

Table S1. Relation between inhibition modality, α value, and diagnostic signature on the double-reciprocal plots (i.e Lineweaver-Burk plot) [S2].

α	Inhibition modality	Diagnostic signature on double-reciprocal plot
$\alpha = 1$	**noncompetitive** (inhibitor binds to free enzyme and enzyme-substrate complex with equal affinity)	lines converge at the x-axis
$\alpha > 1$	**mixed mode inhibitor** (inhibitor can bind to free enzyme and enzyme-substrate complex unequally), inhibitor's affinity is higher for free enzyme	lines converge to the left of the y-axis and above the x-axis
$\alpha < 1$	**mixed mode inhibitor** (inhibitor can bind to free enzyme and enzyme-substrate complex unequally), inhibitor's affinity is higher for the enzyme-substrate complex)	lines converge to the left of the y-axis and below the x-axis
$\alpha \to \infty$	**competitive** (inhibitor and substrate compete for the same site of binding)	lines converge at the y-axis
$\alpha \to 0$ and $\alpha > 0$	**uncompetitive** (inhibitor binds only to the enzyme-substrate complex)	parallel lines

References:

S1. Copeland, R.A. Enzymes: A Practical Introduction to Structure, Mechanism, and Data Analysis.; 2nd ed.; Willey-VCH: New York / Chichester / Weinheim / Brisbane / Singapore / Toronto, 2000; ISBN 0-471-22063-9.

S2. Copeland, R.A. Evaluation of Enzyme Inhibitors in Drug Discovery. A Guide for Medicinal Chemists and Pharmacologists, John Wiley & Sons, Inc., Hoboken, New Jersey, **2005.**
